# Comprehensive genetic analysis of the human lipidome identifies loci associated with lipid homeostasis with links to coronary artery disease

**DOI:** 10.1038/s41467-022-30875-7

**Published:** 2022-06-06

**Authors:** Gemma Cadby, Corey Giles, Phillip E. Melton, Kevin Huynh, Natalie A. Mellett, Thy Duong, Anh Nguyen, Michelle Cinel, Alex Smith, Gavriel Olshansky, Tingting Wang, Marta Brozynska, Mike Inouye, Nina S. McCarthy, Amir Ariff, Joseph Hung, Jennie Hui, John Beilby, Marie-Pierre Dubé, Gerald F. Watts, Sonia Shah, Naomi R. Wray, Wei Ling Florence Lim, Pratishtha Chatterjee, Ian Martins, Simon M. Laws, Tenielle Porter, Michael Vacher, Ashley I. Bush, Christopher C. Rowe, Victor L. Villemagne, David Ames, Colin L. Masters, Kevin Taddei, Matthias Arnold, Gabi Kastenmüller, Kwangsik Nho, Andrew J. Saykin, Xianlin Han, Rima Kaddurah-Daouk, Ralph N. Martins, John Blangero, Peter J. Meikle, Eric K. Moses

**Affiliations:** 1 School of Population and Global Health, University of Western Australia, Crawley, WA Australia; 2 Baker Heart and Diabetes Institute, Melbourne, VIC Australia; 3 Baker Department of Cardiometabolic Health, University of Melbourne, Melbourne, VIC Australia; 4 Menzies Research Institute, University of Tasmania, Hobart, TAS Australia; 5 School of Biomedical Sciences, University of Western Australia, Crawley, WA Australia; 6 School of Women’s and Children’s Health, University of New South Wales, Sydney, NSW Australia; 7 School of Medicine, The University of Western Australia, Crawley, WA Australia; 8 Department of Cardiovascular Medicine, Sir Charles Gairdner Hospital, Perth, WA Australia; 9 Busselton Population Medical Research Institute Inc., Perth, WA Australia; 10 PathWest Laboratory Medicine WA, Perth, WA Australia; 11 Université de Montréal Beaulieu-Saucier Pharmacogenomics Centre, Montreal Heart Institute, Montreal, QC Canada; 12 Lipid Disorders Clinic, Department of Cardiology, Royal Perth Hospital, Perth, WA Australia; 13 Institute for Molecular Biosciences, University of Queensland, Brisbane, QLD Australia; 14 Queensland Brain Institute, University of Queensland, Brisbane, QLD Australia; 15 School of Medical and Health Sciences, Edith Cowan University, Joondalup, WA Australia; 16 Cooperative research Centre (CRC) for Mental Health, Joondalup, WA Australia; 17 Department of Biomedical Sciences, Macquarie University, North Ryde, NSW Australia; 18 KaRa Institute of Neurological Disease, Sydney, Macquarie Park, NSW Australia; 19 Centre for Precision Health, Edith Cowan University, Joondalup, WA Australia; 20 Collaborative Genomics Group, School of Medical and Health Sciences, Edith Cowan University, Joondalup, WA Australia; 21 Curtin Health Innovation Research Institute, Curtin University, Perth, WA Australia; 22 The Australian e-Health Research Centre, Health and Biosecurity, CSIRO, Floreat, WA Australia; 23 The Florey Department of Neuroscience and Mental Health, The University of Melbourne, Melbourne, VIC Australia; 24 Department of Molecular Imaging and Therapy, Austin Health, Heidelberg, VIC Australia; 25 Department of Medicine, Austin Health, The University of Melbourne, Heidelberg, VIC Australia; 26 National Ageing Research Institute, Parkville, VIC Australia; 27 University of Melbourne Academic Unit for Psychiatry of Old Age, St George’s Hospital, Kew, VIC Australia; 28 Department of Psychiatry and Behavioral Sciences, Duke University, Durham, NC USA; 29 Institute of Computational Biology, Helmholtz Zentrum München, German Research Center for Environmental Health, Neuherberg, Germany; 30 Department of Radiology and Imaging Sciences, Indiana University School of Medicine, Indianapolis, IN USA; 31 Center for Computational Biology and Bioinformatics, Indiana University School of Medicine, Indianapolis, IN USA; 32 Indiana Alzheimer’s Disease Research Center, Indiana University School of Medicine, Indianapolis, IN USA; 33 Department of Medical and Molecular Genetics, Indiana University School of Medicine, Indianapolis, IN USA; 34 Barshop Institute for Longevity and Aging Studies, University of Texas Health Science Center at San Antonio, San Antonio, TX USA; 35 Duke Institute of Brain Sciences, Duke University, Durham, NC USA; 36 Department of Medicine, Duke University, Durham, NC USA; 37 South Texas Diabetes and Obesity Institute, The University of Texas Rio Grande Valley, Brownsville, TX USA; 38 Monash University, Melbourne, VIC Australia

**Keywords:** Lipidomics, Mass spectrometry, Cardiovascular diseases, Genome-wide association studies

## Abstract

We integrated lipidomics and genomics to unravel the genetic architecture of lipid metabolism and identify genetic variants associated with lipid species putatively in the mechanistic pathway for coronary artery disease (CAD). We quantified 596 lipid species in serum from 4,492 individuals from the Busselton Health Study. The discovery GWAS identified 3,361 independent lipid-loci associations, involving 667 genomic regions (479 previously unreported), with validation in two independent cohorts. A meta-analysis revealed an additional 70 independent genomic regions associated with lipid species. We identified 134 lipid endophenotypes for CAD associated with 186 genomic loci. Associations between independent lipid-loci with coronary atherosclerosis were assessed in ∼456,000 individuals from the UK Biobank. Of the 53 lipid-loci that showed evidence of association (*P* < 1 × 10^−3^), 43 loci were associated with at least one lipid endophenotype. These findings illustrate the value of integrative biology to investigate the aetiology of atherosclerosis and CAD, with implications for other complex diseases.

## Abstract (web summary)

Dysregulation of lipid metabolism is associated with coronary artery disease (CAD). Here, the authors perform GWAS of the serum lipidome to identify variants associated with lipid species that are putatively in the mechanistic pathway to CAD.

## Introduction

Lipids comprise thousands of individual species, spanning many classes and subclasses. Genome-wide association studies (GWAS) of lipid species can provide novel insights into human physiology, inborn errors of metabolism and mechanisms for complex traits and diseases. Dyslipidaemia, a broad term for disordered lipid and lipoprotein, is a major risk factor for atherosclerotic cardiovascular disease and a therapeutic target for the primary and secondary prevention of coronary artery disease (CAD)^1,2^. Defined by elevated low-density lipoprotein (LDL) cholesterol and triglycerides with decreased high-density lipoprotein (HDL) cholesterol —these ‘clinical lipid’ measures provide only a partial view of the complex lipoprotein structures and their metabolism. Lipidomic technologies can now measure hundreds of individual molecular lipid species that make up the human lipidome, providing a more complete snapshot of the underlying lipid metabolism occurring within an individual.

Genome-wide association studies have uncovered thousands of genetic variants linked to traditional clinical lipids (LDL-cholesterol, HDL-cholesterol, triglycerides)^3,4^. Genes implicated at these loci show functional links between lipid levels and CAD^5^. The human lipidome is heritable and predictive of CAD, furthering our understanding of the biology of CAD^6^. The individual lipid species that make up the lipidome are biologically simpler measures that may reside closer to the causal action of genes, making them valuable endophenotypes for gene identification. Genetic interrogation of the human lipidome may therefore reveal further genetic variants that play a role in lipid metabolism and CAD.

Compared with other complex traits, relatively few genomic loci have been associated with lipid species in GWAS of the human serum/plasma lipidome^7–17^, although these studies have generally interrogated a restricted subset of lipid species. The serum lipidome is complex and consists of many isobaric and isomeric species that share elemental composition but are structurally distinct. Existing lipidomic studies often employ techniques that provide poor resolution of these species, limiting their biological interpretation. We have recently expanded our lipidomic platform to better characterise isomeric lipid species, now measuring 596 lipids from 33 classes^18^. Our methodology focuses on the precise measurement of a broad number of lipid and lipid-like compounds, utilising extensive chromatographic separation.

Here, we report a GWAS of 596 targeted lipid species (across 33 lipid classes) in an Australian population-based cohort of 4492 individuals, validation of significant loci in two independent cohorts and a meta-analysis of all results. Using robust procedures, we disentangle the genetic effects of lipid species from lipoproteins. Integration of multiple datasets, including expression quantitative trait loci (eQTL), methylation QTL (meQTL), and protein QTL (pQTL), and in-depth analysis of significant loci highlights putative susceptibility genes for CAD. We demonstrate robust associations between lipid species and CAD using genetic correlations, polygenic risk scores and phenotypic associations. Many lipid-associated loci show pleiotropy with CAD in co-localisation analysis. Assessment of loci with coronary atherosclerosis in 456,486 UK Biobank participants reveals genetic associations, independent of clinical lipid measures.

## Results

### Lipidomic profiling

We measured 596 individual lipid species within 33 lipid classes, covering the major glycerophospholipid, sphingolipid, glycerolipid, sterol, and fatty acyl classes in serum and plasma samples from three independent cohorts (Supplementary Table 1, Supplementary Data 1, 2). Assay performance was monitored using pooled plasma quality control samples, enabling the determination of coefficient of variation (%CV) values for each lipid class and species. In the Busselton Health Study (BHS) discovery cohort, the median %CV was 8.6% with 570 (95.6%) lipid species showing a %CV less than 20%. All lipids were measured in every individual, with the exception of three values which were below the limit of detection. The lipidomic analysis of the Australian Imaging, Biomarker, and Lifestyle (AIBL) and Alzheimer’s Disease Neuroimaging Initiative (ADNI) validation cohorts showed similar assay performance^19^.

### Discovery of genome-wide association study of the human serum lipidome

**Fig. 1:**
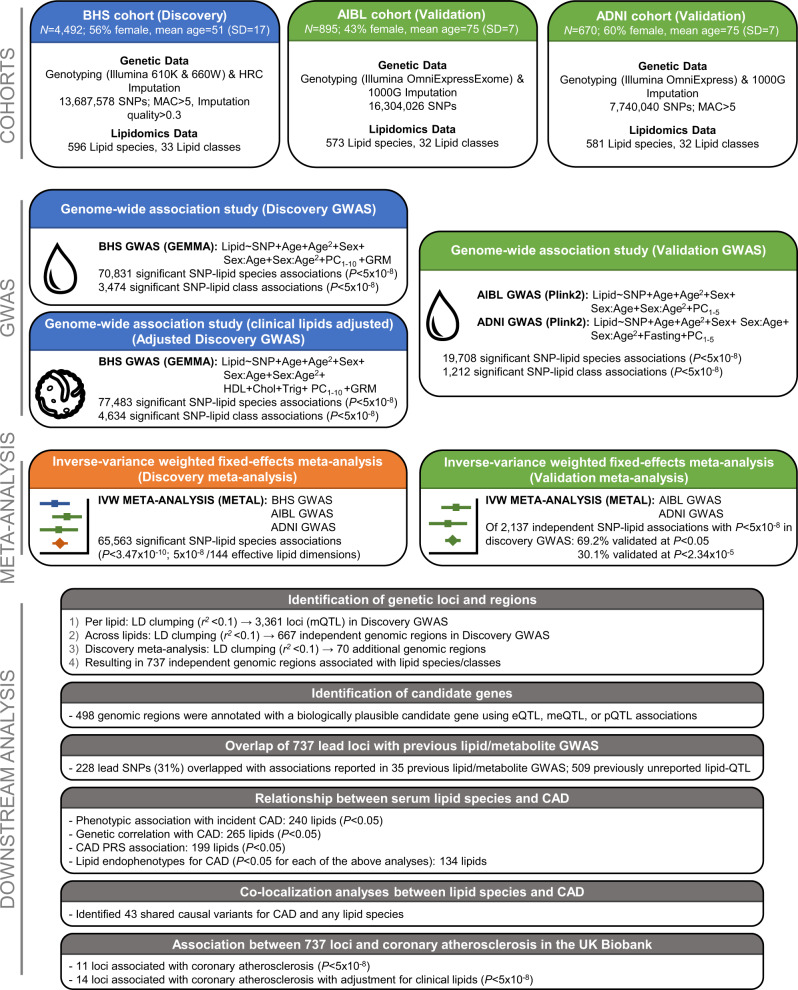
Study design for the genetic analysis of the human lipidome. Representation of genome-wide association studies (GWAS) of the lipidome in the BHS discovery cohort (blue boxes), ADNI and AIBL validation cohorts (green boxes), discovery meta-analysis (orange box), and downstream analyses (grey boxes). ADNI Alzheimer’s Disease Neuroimaging Initiative, AIBL Australian Imaging, Biomarker & Lifestyle Flagship Study of Ageing, BHS Busselton Health Study, CAD coronary artery disease, Chol cholesterol, eQTL expression quantitative trait loci, GRM genetic relatedness matrix, GWAS genome-wide association study, IVW inverse-variance weighted, LD linkage disequilibrium, MAC minor allele count, meQTL methylation quantitative trait loci, mQTL metabolite quantitative trait loci, PC principal component, PRS polygenic risk score, pQTL protein quantitative trait loci, SD standard deviation, SNP single nucleotide polymorphism, Trig triglycerides.

We performed a GWAS of the human serum lipidome (Fig. 1) in the BHS discovery cohort (4492 individuals of European ancestry) followed by validation against a meta-analysis of the two validation cohorts (ADNI and AIBL; 670 and 895 individuals of European ancestry, respectively). We further performed a discovery meta-analysis of all three studies. All summary-level statistics are available at our PheWeb^20^ data portal (https://metabolomics.baker.edu.au/).

**Fig. 2:**
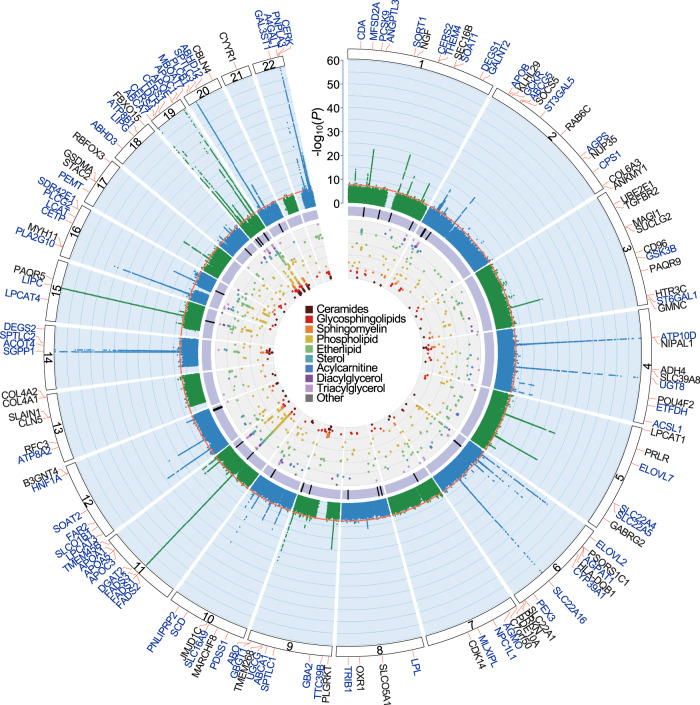
Circular presentation of loci associated with circulating lipid species identified in our Discovery GWAS. The −log_10_(*P*) for genetic association with lipid species are arranged by chromosomal position, indicated by alternating blue and green points. Association *P*-values are truncated at *P* < 1 × 10^−60^. Genome-wide significance (*P* < 5 × 10^−8^) is indicated by the red line. For details about significant associations, see Supplementary Data 2, 3. Genes identified in our candidate gene analysis are highlighted in blue, otherwise the closest gene is indicated in black. The purple band indicates lipid-loci that co-localise with coronary artery disease (CAD) or show association with CAD after adjusting for clinical lipids. The inner circle shows a Fuji plot of SNP-lipid associations, coloured by broad lipid category. Colour keys representing broad lipid categories are indicated in the plot centre. Chromosomes are indicated by numbered panels 1–22.

Within the discovery GWAS, 70,831 genome-wide significant SNP-lipid species and 3474 SNP-lipid class associations were identified (*P* < 5.0 × 10^−8^; Fig. 2). All lipid classes and 543 (of 596; 91.1%) lipid species had at least one significant association (Supplementary Data 3, 4). All significantly associated SNPs were in Hardy-Weinberg Equilibrium (HWE; all *P* ≥ 1.53 × 10^−4^) and were relatively common (minor allele frequency; MAF < 0.01: 4%; MAF > 0.05: 91%, Supplementary Data 5). LD-clumping identified 2279 independent SNP-lipid species associations, and 132 independent SNP-lipid class associations at a genome-wide significance (*P* < 5.0 × 10^−8^; *r*^*2*^ < 0.1; Fig. 2; Supplementary Data 6).

Each SNP was associated with between 1 and 222 lipids (Supplementary Fig. 1). SNPs associated with a large number of lipids were in regions known to be involved in lipid regulation, including *FADS1/FADS2/FADS3, APOE*, and *LIPC*. The most significant associations were observed between PC(18:0_20:4) and rs174564 (*FADS2*; *P* = 4.63 × 10^−220^) and between Cer(d19:1/22:0) and the intergenic SNP rs364585 (flanking *SPTLC3*; *P* = 7.81 × 10^−185^). In fact, the most significant 26 SNP-lipid species associations were with SNPs in these two regions.

The median genomic inflation factors were 1.01 (range: 0.99–1.03), and 1.02 (range: 1.00–1.03) for lipid species and class analyses, respectively. SNP-based heritability estimates were moderately correlated (*r* = 0.45) with lambda estimates, for each of the lipid species and classes (Supplementary Fig. 2a), as expected^21^.

### SNP-lipid species associations are largely independent of clinical lipid measures

**Fig. 3:**
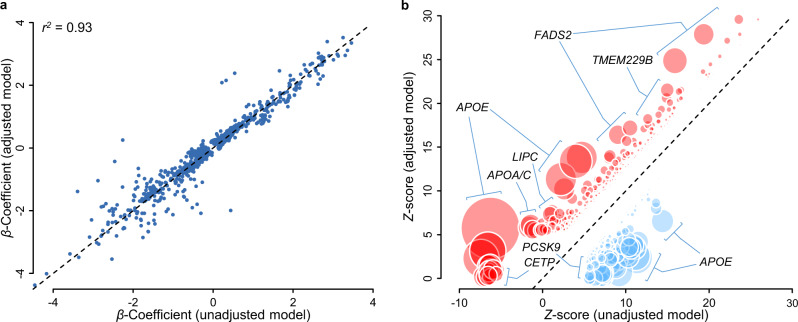
Comparison of estimated lipidomic effect sizes between clinical lipid adjusted and unadjusted models. **a** Beta coefficients for independent unadjusted SNP-lipid associations (*x*-axis) are plotted against clinical lipid-adjusted SNP-lipid associations (*y*-axis). **b** *Z*-scores for unadjusted SNP-lipid associations (*x*-axis) are plotted against clinical lipid-adjusted SNP-lipid associations (*y*-axis). *Z*-scores for SNP associations reaching genome-wide significance (*P* < 5 × 10^−8^) in either the clinical lipid adjusted or unadjusted models. Variant effect signs are fixed so adjusted associations are positive. Variants showing greater (positive) associations in clinical lipid-adjusted analysis are shown in red, and variants showing reduced associations are shown in blue. Circle diameter is proportional of −log_10_(*P*) *t*-test of effect differences.

We performed an additional GWAS, adjusting for clinical lipids (total cholesterol, HDL-cholesterol, triglycerides), to identify SNP-lipid species associations independent of clinical lipid traits (Adjusted Discovery GWAS). The median genomic inflation factors were 1.01 (range: 0.99–1.03), and 1.01 (range: 1.00–1.03) for lipid species and classes, respectively; with heritability estimates moderately correlated (*r* = 0.51) with lambda estimates, for each of the lipid species and classes (Supplementary Fig. 2b). Adjustment for clinical lipids identified 2424 independent SNP-lipid species associations, and 124 independent SNP-lipid class associations (Supplementary Data 6). There were 1545 SNP-lipid species and 72 SNP-lipid class associations that were significant in both the unadjusted and the adjusted analyses, with an *r*^*2*^ between beta coefficients of 0.93 (Fig. 3). Adjustment for clinical lipids identified an additional 879 significant SNP-lipid species associations, for 387 lipid species. However, 726 SNP-lipid species associations previously associated in the unadjusted analysis, fell below our significance threshold. Approximately 24% of these lipid species are members of the cholesteryl ester (*n* = 93), and phosphatidylcholine (*n* = 81) classes (Supplementary Data 6). We also identified an additional 52 significant SNP-lipid class associations, particularly for trihexosylceramide (6 associations) and hexosylceramide (6 associations) classes. However, 60 SNP-lipid class associations fell below our significance threshold, with the classes diacylglycerol, G_M3_ ganglioside, lysophosphatidylcholine, lysoalkenylphosphatidylethanolamine, phosphatidylcholine, alkylphosphatidylethanolamine, alkenylphosphatidylethanolamine, phosphatidylserine, sphingomyelin, and triacylglycerol no longer associated (*P* < 5.0 × 10^−8^) with any genetic variants.

Results from multi-trait conditional and joint (mtCOJO; Supplementary Data 3, 4) analyses using clinical lipid traits (total cholesterol, HDL-cholesterol, triglycerides) GWAS results from the UK Biobank, to minimise the risk of pleiotropy/collider bias introduced by heritable covariates, were largely consistent with those of the clinical lipid-adjusted analysis (*r*^*2*^ of beta coefficients = 0.91, Supplementary Fig. 3). A comparison of the clinical lipid-adjusted *Z*-scores and mtCOJO *Z*-scores identified three gene regions (*APOE*, *FADS1*/*FADS2*/*FADS3*, *TMEM229B*/*PLEKHH1*) with substantial differences (*P* < 1.0 × 10^−4^) indicating the possibility of biased effect measures for the adjusted analyses in these regions. Overall, results were overwhelmingly consistent between mtCOJO and clinical lipid-adjusted analyses.

Conditional analysis (sequentially conditioning on the lead SNP) identified 386 secondary signals (across both unadjusted and clinical lipid-adjusted analyses), associated with 163 lipid species/classes (Supplementary Data 7). Two gene regions, *LIPC* and *ATP10D*, each contained five independent signals (*P*_CONDITIONAL_ < 5.0 × 10^−8^). The *LIPC* genomic region was strongly associated with phosphatidylethanolamine species and class, while *ATP10D* was associated with hexosylceramide species and class. The *SPTLC3* region harboured four independent signals, strongly associated with sphingolipids containing a d19:1 sphingoid base.

### Associations validated in independent cohorts

For each lipid, significantly associated SNPs were linkage disequilibrium (LD)-clumped to remove variants in LD (*r*^*2*^ > 0.1). We assessed whether the 2411 independent lipid species/class associations identified in the BHS discovery cohort (unadjusted) were validated within a combined ADNI and AIBL validation cohort meta-analysis (Validation meta-analysis). There were 273 SNP-lipid associations not available for validation in the meta-analysis, either due to lipids not available in the ADNI and AIBL cohorts; missing SNPs (and proxies) on the imputation panel; or monomorphic/very-low-frequency MAF in ADNI/AIBL. Therefore, we attempted to validate the remaining 2137 significant SNP-lipid associations. We considered a SNP-lipid association to be validated if (i) the SNP was significantly associated (*P* < 5 × 10^−8^) in the unadjusted BHS discovery GWAS; (ii) the direction of effect was concordant between the validation meta-analysis and the BHS discovery analysis; and (iii) the association was nominally significant (*P* < 0.05; less conservative) or reached the Bonferroni significance threshold (*P* < 2.34 × 10^−5^) in the validation meta-analysis. We identified 1474 (69.2%) SNP-lipid associations that reached nominal significance (*P* < 0.05), and 644 (30.1%) reaching Bonferroni-corrected significance (Supplementary Data 8). Almost all associations (>99%) had the same direction of effect, with a very strong correlation between validation meta-analysis and significant (*P* < 5 × 10^−8^) discovery effect sizes (*r*^*2*^ = 0.53 overall, and *r*^*2*^ = 0.80 for SNPs with MAF > 0.05 in the BHS; Supplementary Fig. 4).

### Discovery meta-analysis

At a stringent significance threshold of *P* < 3.47 × 10^−10^ (5 × 10^−8^/144 effective lipid dimensions), the meta-analysis of all three studies identified 65,563 significant SNP-lipid associations (Supplementary Data 9), involving 499 lipid species/classes and 7600 SNPs. We identified 5658 new associations not observed in the BHS discovery GWAS alone, involving 352 lipids and 2914 SNPs. The majority of these (*n* = 5543; 98%) showed some evidence of association in the BHS discovery GWAS (5 × 10^−8^ < *P* < 5 × 10^−4^). However, 89 associations were not nominally significant (*P* > 0.05) in the BHS discovery GWAS, indicating that the effects observed in the meta-analysis were largely due to the AIBL and ADNI samples.

### Defining independent loci and genes controlling lipid homeostasis

For each lipid, significantly associated SNPs were LD-clumped to remove variants in LD (*r*^*2*^ > 0.1). Lead variants from the BHS discovery GWAS (adjusted and unadjusted) and conditional analyses, were clumped if the index SNPs were in linkage disequilibrium (*r*^*2*^ > 0.1). We identified 3361 independent loci-lipid associations, involving 610 lipid species/classes, each associated with between 1 and 30 independent SNPs. To identify genomic regions associated with lipid metabolism, a single dataset was produced by identifying the smallest *P*-value for each SNP across all lipids and analyses. LD-clumping of this dataset resulted in 667 independent genomic regions (Supplementary Data 10; filtered by column ‘Lead SNP in BHS GWAS’). This procedure was repeated, including SNP-lipid associations passing our discovery meta-analysis significance threshold (*P* < 3.47 × 10^−10^), resulting in 682 independent genomic regions (Supplementary Data 10; filtered by column ‘Lead SNP in Discovery-Meta analysis’), 612 of which overlap with those identified in BHS alone (737 in total). The variants within a genomic region and the lipids associated with those variants are collectively termed a genetically influenced lipotype.

### Identification of candidate genes within loci

**Fig. 4:**
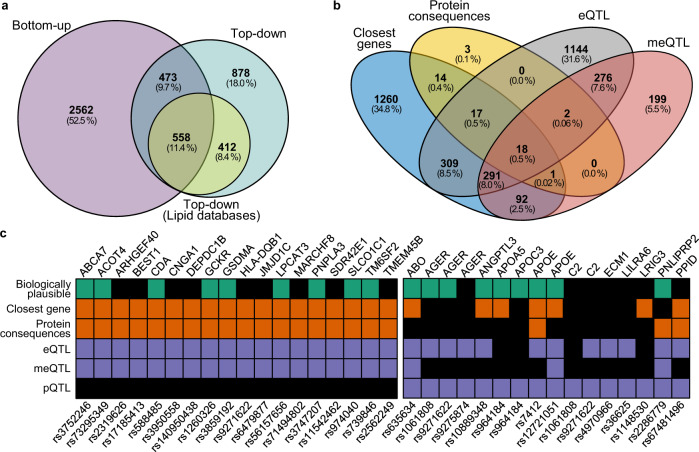
Identification of putative causal genes using genetic prioritisation and knowledge-based approaches. Assignment of putative causal genes was performed using the ProGeM framework, incorporating genetic-based prioritisation (bottom-up), and biological knowledge-based approaches (top-down). **a** Venn diagram showing the number of loci with annotations for candidate genes using the distinct approaches and the overlap. Top-down annotations were divided into lipid-specific databases and generic databases. **b** Venn diagram of distinct genes identified in genetic-based prioritisation analysis. **c** Summary of putative causal genes with overlapping annotations for closest gene, protein consequences, eQTL and meQTL (left). Summary of putative causal SNP-gene pairs for which pQTL evidence was identified (right). eQTL expression quantitative trait loci, meQTL methylation quantitative trait loci, pQTL protein quantitative trait loci.

Using the prioritisation of candidate causal Genes at Molecular QTLs (ProGeM) framework^22^ to prioritise candidate causal genes, biologically plausible genes were identified in 573 of the 737 genomic regions (Supplementary Data 10-12), with an overlap of 498 genomic regions between genetic-based (bottom-up) and biological knowledge (top-down) based approaches. A total of 2321 SNP-gene pairs were identified, where the gene has previously been implicated in the regulation of metabolism or a molecular phenotype (Fig. 4a). Of these genes, 970 (41.8%) are present in lipid-metabolism-specific databases.

A total of 62 SNPs were annotated as either missense (*n* = 59), stop gain (*n* = 2), structural interaction (*n* = 1), start loss (*n* = 1), or splice donor (*n* = 1) mutations. Of these, three were annotated as having a putative ‘high’ impact, and the remaining as ‘moderate’ impact. These SNPs are linked to 55 protein products (Fig. 4b).

Comparing our lead SNPs and proxies against previously published eQTL associations, 2058 SNP-gene pairs were identified (Fig. 4b). Published meQTL associations revealed 879 SNP-gene pairs, 587 (66.8%) of which replicated eQTL associations. In contrast to eQTL and meQTL, the overlap of published pQTL associations was much less evident, with only 16 SNP-gene pairs identified (Fig. 4c). In total, 18 SNP-gene pairs were identified with evidence from the closest gene, protein consequences, eQTL and meQTL. The overlap of top-down and bottom-up candidates supported the annotation of 1031 SNP-gene pairs.

### Most SNP-lipid species associations have not been previously reported

For each of the 737 lead variants, we assessed whether they (or their proxies) had been previously reported as being associated with any lipid or metabolite. From 35 previous metabolomic/lipidomic studies (Supplementary Table 2), 228 lead variants (31%) had been reported as associating with a lipid or metabolite, resulting in 509 unreported genetically influenced lipotypes (Supplementary Data 13).

### Genetically influenced lipotypes overlap with coronary artery disease and cardiovascular disease-related loci

We looked at the overlap between 10 hard cardiovascular disease (CVD) endpoints from the GWAS Catalog and the lead SNP (or proxy) from each of the 737 regions, identifying a total of 23 lead SNPs, or their proxies, associated (*P* < 5 × 10^−8^) with 10 hard CVD endpoints (Supplementary Data 14). The most frequently overlapping GWAS Catalog hard CVD endpoints were CAD (*n* = 14 SNPs), CVD (*n* = 10 SNPs), coronary artery calcification (*n* = 8 SNPs), and myocardial infarction (*n* = 8 SNPs). Three additional lead SNPs were associated with CAD in the CARDIoGRAMplusC4D and UK Biobank meta-analysis. Eighty-four lead SNPs were associated with 101 CVD-related traits, including chronic kidney disease (*n* = 18,) C-reactive protein (*n* = 14), metabolic syndrome (*n* = 12), body mass index (*n* = 8), and systolic blood pressure (*n* = 4). As expected, lead SNPs frequently overlapped with 186 lipid-related traits, with 99 lead SNPs or proxies observed in the GWAS Catalog.

### Serum lipid species/classes are phenotypically and genetically associated with coronary artery disease

**Fig. 5:**
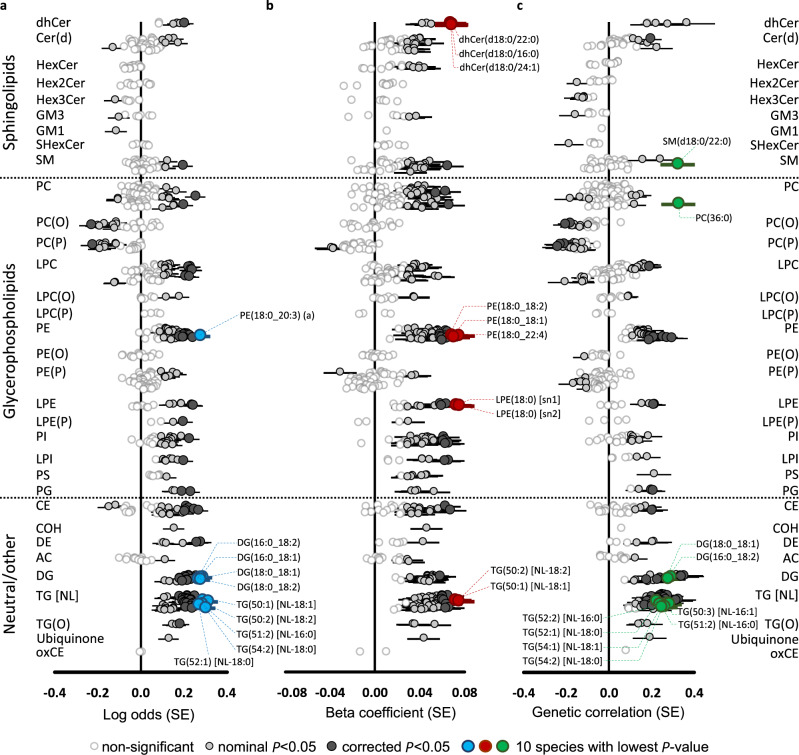
Genetic and phenotypic associations of the lipidome with coronary artery disease. Forest plots of lipid-coronary artery disease; circles represent effect sizes and horizontal bars represent ±standard errors. **a** Phenotypic associations (logistic regression; two-sided) between lipid species and incident coronary artery disease in the BHS cohort (551 cases and 3703 controls), adjusted for age, sex, and the first 10 genomic principal components. **b** Association of lipid species with polygenic risk for coronary artery disease. Individuals in the discovery cohort (*n* = 4492) were assessed for risk using the metaGRS polygenic score, consisting of ∼1.7 million genetic variants. Linear regressions (two-sided) were performed to test the association between an individual’s polygenic score and lipid species concentrations, adjusting for age, sex, and the 10 first principal components. **c** Genetic correlations of lipid species (*n* = 4492) against coronary artery disease (meta-analysis of CARDIoGRAMplusC4D and UK Biobank; 122,733 cases and 424,528 controls), performed with Linkage Disequilibrium Score Regression (LDSC; v1.0.1). Nominally significant and Benjamini–Hochberg corrected significance is indicated by light- and dark-grey circles, respectively. The 10 most significant lipid species are highlighted in blue, red, or green.

Using nominal significance (*P* < 0.05), we identified 243 lipid species/classes phenotypically associated with incident CAD in the BHS (Fig. 5a; Supplementary Data 15), with 88% in the positive direction. The strongest association was between TG(50:2) [NL-18:2] and incident CAD (0.311 ± 0.046, *P* = 1.74 × 10^−11^, FDR *q* = 1.09 × 10^−8^). Overall, the most strongly associated lipid species were those in the triacylglycerol, diacylglycerol, phosphatidylethanolamine, and cholesteryl ester classes.

We identified 265 lipid species/classes that showed a nominally significant (*P* < 0.05) association with the CAD polygenic risk score^23^ in the BHS (Fig. 5b; Supplementary Data 15). These were positive associations except for lipids in the alkenyl-phosphatidylcholine and alkenyl-phosphatidylethanolamine classes. The strongest association was observed for LPE(18:0) [sn2] (0.075 ± 0.014, *P* = 8.9 × 10^−8^, FDR *q* = 5.59 × 10^−5^).

Next, we estimated the genetic correlation between lipid species/classes and CAD. Using linkage disequilibrium score regression, we identified nominally significant genetic correlations (*P* < 0.05) between 199 lipid species/classes and CAD, with 50 of these negatively correlated (Fig. 5c; Supplementary Data 15). The strongest genetic correlations were between TG(51:2) [NL-16:0] (0.275 ± 0.058, *P* = 2.22 × 10^−6^, FDR *q* = 8.94 × 10^−4^) and CAD.

Overall, using a significance threshold of *P* < 0.05, we identified 134 lipid species/classes that were significantly associated in each of the three analyses—association with incident CVD (phenotypic), CAD polygenic risk (PRS), and genetic correlation. Importantly, these lipid species/classes showed concordant directions of effects in all three analyses, defining these lipid species/classes as lipid endophenotypes for CAD.

### Co-localisation analysis identified shared causal variants for coronary artery disease

**Fig. 6:**
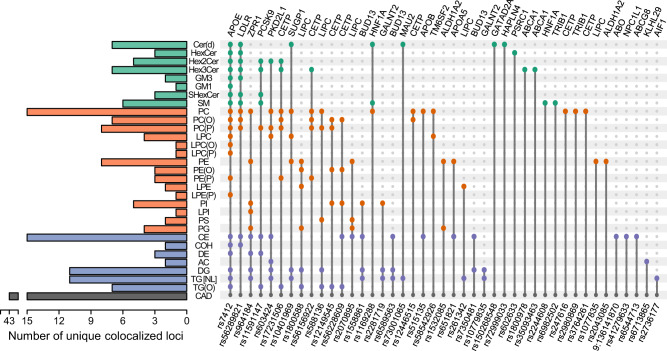
Co-localisation of lipid-loci with coronary artery disease. Summary of lipid classes which contain at least one lipid specie that co-localises with coronary artery disease. Colours indicate broad lipid categories—green, sphingolipids; orange, phospholipids; blue, neutral lipids/others. Indicated variants were identified as the most likely causal variant for each of the identified co-localisation analysis. Genetic variants are ordered according to the number of co-localisations across lipid classes. Evidence of co-localisation included H3 + H4 > 0.8 and H4/H3 > 10.

We performed pairwise co-localisation analysis, within each QTL, between lipid species and CAD to assess whether they share common variants (Supplementary Data 16). We identified evidence of 43 shared variants for CAD and any lipid species (Table 1; Supplementary Note 1; Fig. 6). The strongest evidence was between CE(18:1) and CAD at the *APOE* rs7412 loci (H3 + H4 = 1.00; H4/H3 = 1.17 × 10^11^). There was strong evidence for the sharing of this variant between CAD and 184 lipid species from 23 lipid classes (with and without clinical lipid adjustment). There was also strong evidence for rs603424, near a likely candidate *SCD* (Stearoyl-CoA desaturase), and 24 lipid species/classes (0.936 < H3 + H4 < 0.998; 16 < H4/H3 < 1.8 × 10^3^).

**Table 1:** Genomic regions showing co-localisation with lipid species and coronary artery disease.

#	rsID	Position^a^	EA/ OA	Co-localised lipid classes	Number of lipids co-localised	Strongest co-localisation	Minimum CAD *P*-value in region	Nearby genes^b^
1	rs11591147	1:55505647	G/T	CE, DE, Hex2Cer, Hex3Cer, PC(P), SHexCer, SM, TG(O)	32	CE(18:1)	1.86 × 10^−22^	PCSK9, USP24, BSND
2	rs602633	1:109821511	G/T	HexCer	2	HexCer(d18:1/24:1)	3.63 × 10^−58^	PSRC1, CELSR2, MYBPHL
3	rs2281719	1:230297659	C/T	DG, PI, TG [NL]	5	DG(18:0_18:1)	6.41 × 10^−07^	GALNT2, PGBD5, COG2
4	rs10779835	1:230299949	C/T	DG, TG [NL]	4	TG(54:2) [NL-18:0]	6.41 × 10^−07^	GALNT2, PGBD5, COG2
5	rs515135	2:21286057	C/T	CE, PC	4	PC(16:0_18:0)	5.74 × 10^−17^	APOB, TDRD15, LDAH
6	rs6713865	2:23899807	A/G	AC	2	AC(16:0)	2.86 × 10^−05^	KLHL29, ATAD2B, UBXN2A
7	rs6544713	2:44073881	C/T	CE	6	CE(20:1)	1.84 × 10^−18^	ABCG8, ABCG5, DYNC2LI1
8	rs2736177	6:31586094	C/T	TG [NL]	2	TG(50:2) [NL-18:2]	4.86 × 10^−09^	AIF1, PRRC2A, BAG6
9	rs41279633	7:44580876	G/T	CE	1	CE(18:0)	1.72 × 10^−06^	NPC1L1, DDX56, TMED4
10	rs6982502	8:126479362	C/T	SM	1	SM(d18:0/22:0)	7.67 × 10^−23^	TRIB1, NSMCE2, WASHC5
11	rs2980869	8:126488250	C/T	PC	1	PC(36:0)	7.67 × 10^−23^	TRIB1, NSMCE2, WASHC5
12	rs35093463	9:107586238	A/C	Hex3Cer	2	Hex3Cer(d18:1/22:0)	4.00 × 10^−07^	ABCA1, NIPSNAP3B, NIPSNAP3A
13	rs1800978	9:107665978	C/G	Hex3Cer	1	Hex3Cer(d18:1/24:1)	4.00 × 10^−07^	ABCA1, NIPSNAP3B, NIPSNAP3A
14	9:136141870	9:136141870	C/T	CE	1	CE(18:0)	2.03 × 10^−14^	ABO, SURF6, OBP2B
15	rs603424	10:102075479	A/G	AC, CE, DG, Hex2Cer, LPC, PC, PC(P), TG [NL]	24	LPC(16:1) [sn2]	7.41 × 10^−07^	PKD2L1, BLOC1S2, SCD
16	rs7350481	11:116586283	C/T	CE, DG	2	DG(18:1_18:2)	5.64 × 10^−07^	BUD13, ZPR1, APOA5
17	rs6589563	11:116590787	A/G	CE, DG, TG [NL]	4	DG(18:0_18:1)	5.64 × 10^−07^	BUD13, ZPR1, APOA5
18	rs1558861	11:116607437	C/T	CE, DG, PI, TG [NL]	25	TG(54:4) [NL-18:2]	5.64 × 10^−07^	BUD13, ZPR1, APOA5
19	rs964184	11:116648917	C/G	CE, DE, DG, LPI, PC, PE, PG, PI, TG [NL]	64	TG(54:2) [NL-18:0]	7.03 × 10^−13^	ZPR1, BUD13, APOA5
20	rs651821	11:116662579	C/T	CE, PE	3	CE(22:0)	7.03 × 10^−13^	APOA5, ZPR1, BUD13
21	rs1169288	12:121416650	A/C	Cer(d), PC, SM	6	PC(36:0)	1.26 × 10^−18^	HNF1A, C12orf43, OASL
22	rs2244608	12:121416988	A/G	SM	1	SM(d18:0/22:0)	1.26 × 10^−18^	HNF1A, C12orf43, OASL
23	rs2043085	15:58680954	C/T	PE	1	PE(18:0_18:1)	7.24 × 10^−06^	ALDH1A2, LIPC, AQP9
24	rs1532085	15:58683366	A/G	PE, PG	16	PE(18:1_18:2)	7.24 × 10^−06^	ALDH1A2, LIPC, ADAM10
25	rs1077835	15:58723426	A/G	PE	7	PE(15-MHDA_22:6)	7.24 × 10^−06^	ALDH1A2, LIPC, ADAM10
26	rs1800588	15:58723675	C/T	DG, LPE, PE, PE(O), PG, TG(O)	19	LPE(20:4) [sn1]	7.24 × 10^−06^	ALDH1A2, LIPC, ADAM10
27	rs2070895	15:58723939	A/G	CE, PE, PG, PS	16	PG(34:2)	7.24 × 10^−06^	ALDH1A2, LIPC, ADAM10
28	rs588136	15:58730498	C/T	DG, PC, PC(P), PS, TG(O)	10	Total PC	7.24 × 10^−06^	ALDH1A2, LIPC, ADAM10
29	rs261342	15:58731153	C/G	LPE, TG [NL]	3	LPE(20:4) [sn1]	7.24 × 10^−06^	ALDH1A2, LIPC, ADAM10
30	rs12446515	16:56987015	C/T	PC, PC(O)	3	PC(16:0_16:0)	1.19 × 10^−09^	CETP, HERPUD1, NLRC5
31	rs56156922	16:56987369	C/T	Hex3Cer, PC, PC(O), PC(P), PE(P)	22	PC(P-16:0/16:1)	1.19 × 10^−09^	CETP, HERPUD1, NLRC5
32	rs56228609	16:56987765	C/T	CE, PC(O), PE(O), PI, TG(O)	6	CE(18:0)	1.19 × 10^−09^	CETP, HERPUD1, NLRC5
33	rs247616	16:56989590	C/T	PC	1	PC(16:0_18:3) (a)	1.19 × 10^−09^	CETP, HERPUD1, NLRC5
34	rs12149545	16:56993161	A/G	PC(O), PC(P), PE(O), PI, TG(O)	11	TG(O-50:1) [NL-16:0]	1.19 × 10^−09^	CETP, HERPUD1, NLRC5
35	rs3764261	16:56993324	A/C	PC	1	PC(18:2_18:2)	1.19 × 10^−09^	CETP, HERPUD1, NLRC5
36	rs17231506	16:56994528	C/T	Hex2Cer, Hex3Cer, PC, PC(O), PC(P), PE(P), TG(O)	40	TG(O-50:1) [NL-16:0]	1.19 × 10^−09^	CETP, HERPUD1, NLRC5
37	rs56289821	19:11188247	A/G	CE, Cer(d), COH, GM3, Hex2Cer, Hex3Cer, HexCer, PC, PC(O), PC(P), SHexCer, SM	60	SM(35:2) (b)	1.93 × 10^−36^	LDLR, SMARCA4, SPC24
38	rs72999033	19:19366632	C/T	Cer(d)	1	Cer(d16:1/24:1)	3.18 × 10^−07^	HAPLN4, NCAN, TM6SF2
39	rs58542926	19:19379549	C/T	LPC, PC	2	LPC(20:3) [sn1]	3.18 × 10^−07^	TM6SF2, HAPLN4, SUGP1
40	rs10401969	19:19407718	C/T	Cer(d), DG, LPC, PC, PE, TG [NL]	38	DG(18:1_20:4)	3.18 × 10^−07^	SUGP1, TM6SF2, MAU2
41	rs73001065	19:19460541	C/G	Cer(d), TG [NL]	3	Cer(d18:1/24:0)	3.18 × 10^−07^	MAU2, SUGP1, GATAD2A
42	rs150268548	19:19494483	A/G	Cer(d)	3	Total Cer	3.18 × 10^−07^	GATAD2A, MAU2, SUGP1
43	rs7412	19:45412079	C/T	CE, Cer(d), COH, DE, DG, GM1, GM3, Hex2Cer, Hex3Cer, HexCer, LPC, LPC(O), LPC(P), LPE(P), PC, PC(O), PC(P), PE(P), SHexCer, SM, TG [NL], TG(O)	184	CE(16:0)	2.14 × 10^−35^	APOE, TOMM40, APOC1

Co-localisation analyses performed using coronary artery disease in UK Biobank and CARDIoGRAMplusC4D. Minimum CAD *P*-values were obtained from the meta-analysis performed in van der Harst & Verweij 2018.

*CAD* coronary artery disease, *EA* effect allele, *OA* other allele.

^a^Genomic position based on Genome Reference Consortium Human Build 37 (GRCh37).

^b^Closest three protein coding genes to causal variant.

### Genetically influenced lipotypes were associated with coronary atherosclerosis in the UK Biobank

To further define pleiotropic effects between lipid species and CAD, we performed association analysis of 737 lead SNPs and coronary atherosclerosis in 456,486 participants of the UK Biobank (Supplementary Data 17). Eleven of the lipid-associated SNPs had genome-wide significant (*P* < 5 × 10^−8^) associations with coronary atherosclerosis. Adjustment for clinical lipids (total cholesterol, HDL-cholesterol, triglycerides) increased this number to 17; however, adjustment for clinical lipids using mtCOJO, which is free of the bias introduced by heritable covariates, resulted in only 14 associations with coronary atherosclerosis. Importantly, 11 of these associations were sub-genome-wide significant in the initial analysis, suggesting the presence of strong pleiotropy in these regions. After comparing effect estimates between the standard GWAS and mtCOJO clinical lipid-adjusted analysis, eight lead SNPs (with *P* < 5 × 10^−8^ in the standard GWAS) showed the opposite directions of associations. These regions contain prototypical lipid/lipoprotein regulating genes, such as *APOE*, *CETP*, *LDLR*, and *PCSK9*. Interestingly, for all lead SNPs with marginal association with coronary atherosclerosis (*P* < 1.0 × 10^−3^; with and without conditioning on clinical lipids), 43 (81%) were associated with lipid endophenotypes for CAD.

## Discussion

By integrative analysis of the human lipidome and CAD phenotypes, we have identified candidate risk genes for CAD, providing evidence for the role of these lipid species in the development of CAD. Our high-resolution genome-wide association analyses of the human lipidome have identified 737 independent genomic regions associated with lipid metabolism, of which 509 represent genetic loci not previously associated with lipid metabolism. This is a substantial increase over previous studies with similar or larger sample sizes^7,10,24^. Our expanded lipidomic platform utilises extensive chromatographic separation to increase the diversity of measured lipid species and distinguish lipid isomers and isotopes over those measured in previous studies. Combined with the extended pedigree study design of the BHS, we identify many rare/low-frequency variants with large effect sizes.

The majority (69.2%) of the 2137 SNP-lipid associations identified in our discovery GWAS were validated in a meta-analysis of two independent cohorts. Adjustment for clinical lipids (both as standard covariates and mtCOJO analysis), confirmed that the majority of SNP-lipid associations observed were not acting directly through clinical lipids (i.e. associations were not the result of mediated pleiotropy). Discovery meta-analysis of all three studies identified an additional 5658 SNP-lipid associations (from 122 loci)—involving 352 lipid species—that were not identified in the BHS discovery GWAS alone. Overall, nearly all lipid species (95%) had at least one genome-wide significant SNP association, highlighting the genetic contribution to lipid metabolism and homeostasis.

We identified 134 lipid species/classes showing consistent and significant associations with CAD when assessed with genetic correlation, phenotypic association, and PRS association. These lipids are potential endophenotypes for CAD, which can facilitate the identification of susceptibility genes. Of those loci associated with this subset of lipids, we identified 32 regions with evidence of shared genetic effects (co-localisation) with lipids and CAD. We assessed the association of lipid-loci with coronary atherosclerosis in ~456,000 individuals of the UK Biobank, considering the independence of clinical lipid traits. A total of 53 loci showed evidence of association (*P* < 1 × 10^−3^) in at least one analysis. Of these, 43 loci were associated with at least one of the 134 lipid species identified above.

Our lipidomic profiling provided improved resolution and precision in the measurement of lipid species. Prior studies examined lipid phenotypes that were mixtures of similar, but distinct species; lacked structural characterisation of lipid species, or were contaminated through isotopic overlap. Many of the associations between lipid species and prototypical lipid regulating genes observed in our study—such as *FADS1/FADS2*, *APOE*, and *LDLR*—have been reported in earlier GWAS^7–15,17,24^. With our expanded lipidomic profile, we have built on these earlier studies, identifying many new loci associated with lipid species and classes. Previous studies, containing mis-annotation of lipid species, report associations between SNPs in the *FADS* region and sphingomyelin species as containing a mono-unsaturated (16:1, 18:1, or 20:1) n-acyl chain^8,12^. Here, we show the associations of sphingomyelins with SNPs in the *FADS* gene region are disproportional with species containing the d18:2 sphingoid base. This is supported by recent experimental evidence, suggesting FADS3 is a ceramide-specific desaturase, targeting the sphingoid bases^25,26^. Early dogma suggested the dominant isoform of sphingomyelins was d18:1 leading to the aforementioned annotations (i.e. SM(d18:1/16:1)). However, chromatographic separation and characterisation identify the predominant species as SM(d18:2/16:0)^18^. While these associations are not novel per se, the additional specificity of our lipidomics methodology extends across all lipid species and classes, leading to greater confidence in defining true relationships.

We also observed strong associations between specific sphingolipid isoforms and variants in the *SPTLC3* gene region. Serine palmitoyltransferase long chain base subunits (SPTLC) are a series of enzymes responsible for the de novo synthesis of sphingolipids through condensation of serine with palmitoyl-CoA. Three mammalian isoforms have been identified (SPTLC1-3), which form a heterodimer in situ, of which SPTLC1 is requisite for function^27^. The subunit SPTLC3 was discovered more recently and was thought to facilitate the synthesis of shorter-chain sphingolipids^28^. However, we identify strong associations of SNPs in the *SPTLC3* gene region with atypical sphingolipids, containing a d19:1 sphingoid base (Supplementary Data 4). This supports the recent report that SPTLC3 has broader substrate specificity, with capacity to metabolise branched isomers of palmitate (anteiso-branched-C16)^27^ leading to the synthesis of d19:1 sphingoid bases. The atypical structure of these sphingolipids has previously led to mis-annotation resulting in reported associations of the *SPTLC3* gene with hydroxylated sphingomyelins^10,13,14^, when hydroxylated sphingomyelins in the n-acyl chain are unlikely to exist in human plasma^29^.

**Fig. 7:**
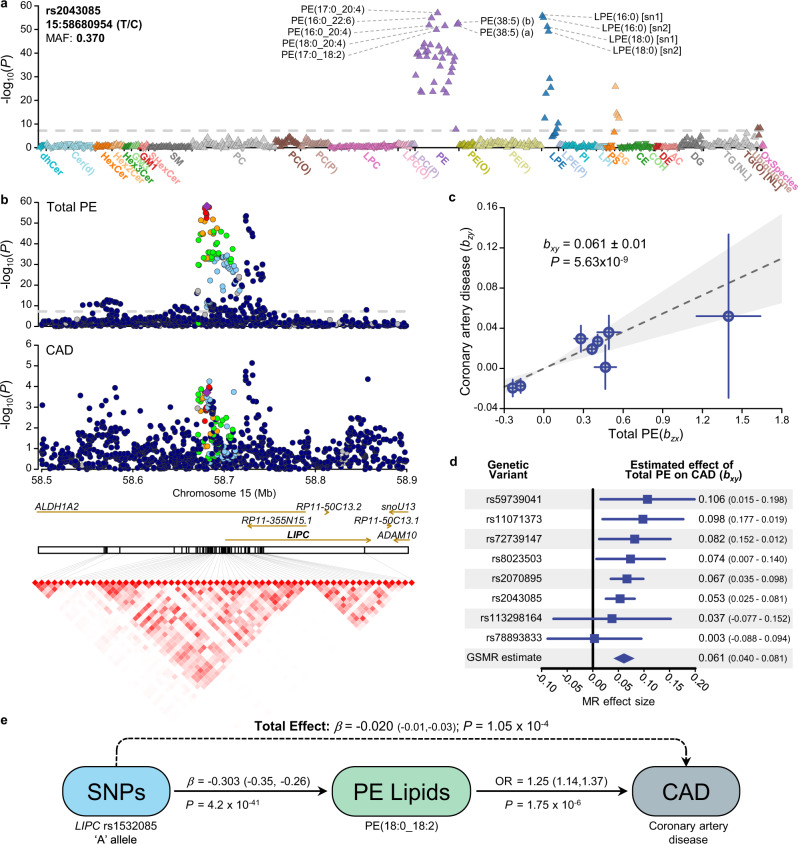
Genetic analysis of the LIPC gene region and circulating levels of phosphatidylethanolamine. **a** Lipid-wide association with the genetic variant, rs2043085, in the BHS cohort (*n* = 4492). Symbol colour is used to distinguish lipid classes. The symbol orientation indicates the effect sign, inverted triangles indicate negative associations, while regular triangles indicate positive associations. The dashed line indicates genome-wide significance (*P* < 5 × 10^−8^). **b** Regional association plots for Total PE and coronary artery disease (van der Harst & Verweij 2018), focusing on the LIPC region. Variants are coloured based on LD with the lead variant, rs2043085. Linkage disequilibrium plot showing correlation between variants following clumping (*r*^*2*^ > 0.8; *P* < 5 × 10^−8^). Variant correlations were obtained from 10,000 unrelated individuals from the UK Biobank. **c** Plot of genetic instrument effect sizes against Total PE (*n* = 4492) and coronary artery disease (*n* = 547,261). Variants were selected based on association with Total PE from within the LIPC region. Eight approximately independent variants were left following clumping (*r*^*2*^ > 0.05; *P* < 5 × 10^−8^). Generalised Summary-data based Mendelian Randomisation (GSMR) was used to estimate effect of Total PE on coronary artery disease, accounting for the variant correlations and uncertainty in both *b*_*zx*_ and *b*_*zy*_. Data are presented as mean ± SE. **d** Forest plot of single variant tests and GSMR estimates from panel c. Data presented as mean ± 95% confidence interval. **e** Diagram of mediated pleiotropy, showing effect sizes estimated across multiple datasets. Exposure modifying variant effect sizes were estimated in the BHS cohort, as well as odds ratio of phosphatidylethanolamine lipid species against incident cardiovascular disease. Total effect represents the sum of genetics effects on coronary artery disease, whether mediated through phosphatidylethanolamine or not. Coronary artery disease effect size was obtained from van der Harst & Verweij 2018. Source data are provided as a Source Data file. MAF minor allele frequency, MR Mendelian randomisation, OR odds ratio, PE phosphatidylethanolamine, SNP single nucleotide polymorphism.

Many genes associated with CAD risk were identified as also associated with lipid species and classes, including *HMGCR*, *PCSK9,* and *LDLR* (Table 1), thereby providing new avenues for investigation into mechanistic pathways. We also provide new evidence to support potential roles for genes not reaching genome-wide significance and identify possible mechanisms linking these genes to CAD; we identified strong associations between ten independent signals in the *LIPC/ALDH1A2/AQP9* gene region with phosphatidylethanolamine, lysophosphatidylethanolamine, and phosphatidylglycerol lipid species independent of clinical lipids. Two lead variants were associated with functional consequences, including a start loss for gene *ALDH1A2* and a missense variant for gene *LIPC*. The *LIPC* gene on chromosome 15 encodes hepatic lipase, which is functionally described as a triglyceride lipase and as possessing phospholipase A1 activity (hydrolyses sn-1 fatty acid from phospholipids). The role of hepatic lipase in lipoprotein remodelling is complex, being intimately involved in HDL-, IDL-, and chylomicron remnant-metabolism^30^. Consequently, the role of hepatic lipase in cardiovascular disease risk has been controversial, with both pro- and anti-atherogenic mechanisms identified^30,31^. These mechanisms are often viewed through the lens of lipoprotein kinetics. However, the associations of variants in the *LIPC* gene region with phosphatidylethanolamine species are independent of lipoprotein metabolism (Supplementary Data 3, 4)—notionally as these lipids are direct substrates for hepatic lipase. Interestingly, the strength of association of *LIPC* variants with coronary atherosclerosis is considerably increased when conditioned on clinical lipids (both standard adjustment and mtCOJO analyses; Fig. 7c, Supplementary Data 17) further supporting a direct mechanistic link. Phenotypically, phosphatidylethanolamine species are associated with incident CAD (Supplementary Data 15), with a direction of effect concordant with the SNP associations (Fig. 7a). Visual comparison of regional association plots and SNP effect scatter plot supports consistent effects (Figs. 7b, d). We selected independent SNPs (*r*^*2*^ < 0.05) in the *LIPC* gene region associated with the phosphatidylethanolamine class and assessed the similarity of effects with CAD (Fig. 7d). Inverse-variance weighted meta-analysis of SNP effects using Generalised Summary-data-based Mendelian Randomisation (GSMR) support strong pleiotropy consistent with a causal relationship (Fig. 7e).

Angiopoietin-like 3 (ANGPTL3) has been implicated in CAD risk, with a deficiency being associated with cardioprotective effects^32–35^. ANGPTL3 acts as an inhibitor to two other lipases, lipoprotein lipase (LPL)—a rate-limiting enzyme in the clearance of triglyceride-rich lipoproteins—and the phospholipase endothelial lipase (LIPG)^36^. Indeed, loss of function mutations in the *ANGPTL3* gene has been linked to hypolipidemia^34^. Most previous research has focused on the lipoprotein modulating effects of ANGPTL3 through LPL. However, a recent Mendelian Randomization analysis, using NMR lipoprotein profiling, revealed a divergence in the metabolic effects of genetic variants in ANGPTL3 and LPL^37^. We recently identified a rare frameshift deletion (rs398122988) associated with decreased ANGPTL3 protein levels in extended Mexican American families^38^; the variant was also associated with a ~1.3 standard deviation decrease in phosphatidylinositol species. In this study, we validate this observation, with SNPs in the *ANGPTL3* gene region associated with a decrease in phosphatidylinositol species, again these associations persisted even after adjustment for clinical lipids (total cholesterol, HDL-cholesterol, triglycerides). Interestingly, we also observe associations of phosphatidylinositol species with SNPs in the *LIPG* region, suggesting a larger metabolic effect of the ANGPTL3-LIPG pathway, at least in fasting subjects. Commonly, phosphatidylinositol species have been studied for their intracellular messaging roles following phosphorylation of the inositol ring by kinases, including PI-3-kinase, which lead to downstream cardio-metabolic effects^39^. However, the role of phosphatidylinositol species in CVD risk is still largely unknown. We have previously observed the change in the ratio of phosphatidylinositol to phosphatidylcholine species as a predictor of CVD risk reduction from statin treatment^40^. Further work is now required to unravel the role of phosphatidylinositol in mediating the effect of these genes on CVD risk.

Limitations to the study warrant mention. First, our samples were restricted to individuals with European ancestry, complicating generalisability to individuals of non-European ancestry. Previous studies^24,41,42^ have shown conservation of lipid-metabolism genetics across different ancestries; however, future studies in non-European ancestry individuals are required. Second, adjustment for many combinations of lipid-lowering medications and doses is not practical. As a majority of lipid-lowering medications were statins and the assumption that medication dose was titrated, a single lipid species/class correction was applied to all individuals taking these medications. However, as only 2% of the BHS discovery cohort were taking lipid-lowering medications, the putative impact is unlikely to be large. A larger proportion of the two validation samples were taking lipid-lowering medications (ADNI: 49%; AIBL: 22%). Nonetheless, a substantial number of our associations were validated; therefore, the single adjustment was also unlikely to have greatly affected our results. Third, we did not have access to an independent validation sample for our discovery meta-analysis. We consider the discovery meta-analysis to be exploratory, with the potential to provide evidence of associations that can be followed up in future studies. Finally, lipidomic profiling was performed on serum in the discovery BHS and validation ADNI cohorts, whereas the validation study AIBL was plasma. While the absolute concentration of some blood metabolites may differ between plasma and serum, measurements are generally highly correlated between matrices^43^. We have previously shown lipid associations are consistent between serum and plasma^19^.

In summary, using our expanded lipidomic profiling platform, we have investigated the largest number of targeted lipid species in a GWAS, and have reported significant genetic associations with lipid species that have not previously been reported in any genetic association studies to date. Our strategy to use lipid species as endophenotypes in the search for CVD genes is the tip of the iceberg. We have previously reported phenotypic associations of lipid species with other complex traits, including diabetes^44^, Alzheimer’s disease^19^, and atrial fibrillation^45^; we believe the same integrative genomics approach may now be used to elucidate the mechanistic underpinnings of lipid metabolism in these and other complex diseases. These data now represent a valuable resource for the future exploration of the genetic analysis of the lipidome to identify lipid metabolic pathways and regulatory genes associated with complex disease and identify new therapeutic targets. To this end we provide all summary statistics and an online searchable resource of association plots of lipid species and classes with genetic variants and regional association plots with individual lipid species and classes (https://metabolomics.baker.edu.au/).

## Methods

### Study populations

Participants in the discovery cohort (*n* = 4492) were all participants of the 1994/95 survey of the long-running epidemiological study, the BHS, for whom genome-wide SNP data, extensive longitudinal phenotype data, and blood serum were available. The BHS is a community-based study in Western Australia that includes both related and unrelated individuals (predominantly of European ancestry) and has been described in more detail elsewhere^46–48^. Informed consent was obtained from all participants and the 1994/95 health survey was approved by the University of Western Australia Human Research Ethics Committee (UWA HREC). The current study was also approved by UWA HREC (RA/4/1/7894) and the Western Australian Department of Health HREC (RGS03656).

The two validation cohorts used in this study were the AIBL study^49^ and the ADNI study^50^; both of which were established to discover biomarkers, health and lifestyle factors for the development, early detection, and tracking of Alzheimer’s disease. The AIBL study is a longitudinal study which recruited 1112 individuals aged over 60 years within Australia. Time points for blood/data collection were every 18 months from baseline. For each individual, lipidomic data obtained from the earliest blood collection was used. At baseline, 768 individuals were characterised as cognitively normal, 133 with mild cognitive impairment and 211 with Alzheimer’s disease. The ADNI study is a longitudinal study, starting in 2004 and recruited 800 individuals at baseline, from sites across the United States of America and Canada. Serum samples obtained at baseline were analysed. Study data analysed here were obtained from the ADNI database, which is available online (http://adni.loni.usc.edu/). For the lipidomics analysis, the AIBL study was deemed low risk (The Alfred Ethics Committee; Project 183/19), and the ADNI study was deemed ‘research not involving human subjects’ (Duke Institute review board; ID:Pro00053208).

### Lipidomic profiling

Targeted lipidomic profiling was performed using liquid chromatography coupled electrospray ionisation-tandem mass spectrometry from fasting blood serum (BHS discovery), fasting blood plasma (AIBL validation), and a combination of fasting and non-fasting blood serum (ADNI validation; 90% fasting, 10% non-fasting). We quantified 596 lipid species (from 33 lipid classes) in the BHS discovery cohort, 573 lipid species (from 32 lipid classes) in the validation AIBL cohort, and 581 lipid species (from 32 lipid classes) in the validation ADNI cohort. Due to strict quality control, lipid species may be removed from a dataset and typically represent very low abundant species and/or those requiring near-optimal chromatographic separation. All lipid classes were consistent across the studies, except for the Oxidised sterol ester which was only available in the discovery BHS cohort. Overall, 596 lipid species were quantified; 570 of which were quantified within all three cohorts; five lipid species were present only within BHS and ADNI; and 21 lipid species were present only in the BHS cohort (Supplementary Data 1, 2).

Lipidomic profiling of each cohort was performed using the standardised methodology described by Huynh et al.^18^. Lipidomic profiling has been described previously for BHS^6^ and ADNI/AIBL^19^. Briefly, 10 μL of serum/plasma was spiked with an internal standard mix (Supplementary Data 1) and lipid species were isolated using a single-phase butanol:methanol (1:1; BuOH:MeOH) extraction^51^. Analysis of serum/plasma extracts was performed on an Agilent 6490 QqQ mass spectrometer with an Agilent 1290 series HPLC, as previously described. Mass spectrometry settings and transitions for each lipid class are shown in Supplementary Data 1. A total of 497 transitions, representing 596 lipid species (BHS discovery), 573 lipid species (AIBL validation), and 581 lipid species (ADNI validation), were measured using dynamic multiple reaction monitoring (dMRM), where data was collected during a retention time window specific to each lipid species. Raw mass spectrometry data were analysed using MassHunter Quant B08 (Agilent Technologies).

### Data integration and cleaning

Lipid concentrations were calculated by relating the area under the chromatographic peak, for each lipid species, to the corresponding internal standard. Correction factors were applied to adjust for differences in response factors, where these were known^18^. In-house pipelines were used for quality control and filtering of lipid concentrations. Across the entire BHS dataset, only three missing values were evident. Lipids below the limit of detection (missing values) were imputed to half the minimum observed value. To remove technical batch variation, the lipid data in each analytical batch (approximately 486 samples per batch) was aligned to the median value in pooled plasma quality control samples included in each analytical run. Unwanted variation in the discovery cohort was identified using a modified remove unwanted variation-2 (RUV-2) approach^52^. In brief, lipid data were residualised in a linear mixed model, against age, sex, body mass index (BMI), clinical lipids and the genetic relatedness matrix (described below) as the random effects. Principal component analysis was performed on the residualised data. The first two components showed clear trends along with samples in collection order. Therefore, variation associated with these first two principal components was removed from the original dataset. Lipid class totals were generated by summing the concentration of the individual species within each class. Validation cohorts were processed in a similar manner.

### Phenotypic variables

Details of the BHS data collection have been published previously^53^. Serum cholesterol and triglycerides were calculated by standard enzymatic methods on a Hitachi 747 (Roche Diagnostics, Sydney, Australia) from fasting blood collected in 1994/95. HDL-cholesterol was determined on a serum supernatant after polyethylene glycol precipitation using an enzymatic cholesterol assay and LDL-cholesterol was estimated using the Friedewald formula^54^. Height and weight (used to calculate BMI) were collected from participants at the time of the interview (1994/95). The use of lipid-lowering medication was recorded at the time of the interview (1994/95). Diagnosis of incident CAD was defined as either hospitalisation or death due to CAD (ICD9: 410-414; ICD10: I20-I25) after the blood collection date (and until June 2015). Hospitalisations and deaths were identified from the Western Australian Department of Health Hospital Morbidity Data Collection and Death Registrations.

### Medication usage adjustment

For individuals taking lipid-lowering medication (BHS, *n* = 108; AIBL, *n* = 198; ADNI, *n* = 328), lipid species and clinical lipid concentrations were adjusted using previously identified effects of lipid-lowering medication. Changes in lipid species and clinical lipids following one year of statin use were calculated from a placebo randomised controlled trial (LIPID study; *n* = 4991)^40^. To calculate correction factors^55^, lipid measures were centred and scaled by the mean and standard deviation of baseline measures (prior to statin usage), and the change in lipid abundance was calculated and regressed on age, sex, BMI, and statin usage. Statin usage beta coefficients (effect of the lipid-lowering medication) were added to standardised lipid species concentrations of the individuals taking lipid-lowering medication in the current study. For lipid species present in both this study and the LIPID study (overlap of 314 lipid species), species-specific correction factors were calculated. For those lipid species not measured in the LIPID study (*n* = 282), class-specific correction factors were used in place of species-specific correction factors i.e. a ceramide-specific correction factor (average beta coefficient of overlapping ceramide species) was used for ceramide species not measured in the LIPID study. Due to the large proportion of ADNI participants taking lipid-lowering medication, we performed a sensitivity analysis, comparing the above correction against residualising lipid concentrations adjusting for medication usage as a covariate (Supplementary Note 2).

### Genotyping and imputation

For the BHS discovery cohort, genotyping was performed on the Illumina Human 610 K Quad-Bead Chip (Illumina Inc., San Diego, CA, USA) at the Centre National de Genotypage in Paris, France (*n* = 1468), and on the Illumina 660 W Quad Array Bead Chip (Illumina Inc., San Diego, CA, USA) at the PathWest Laboratory Medicine WA (Nedlands, WA, Australia (*n* = 3428). Complete linkage clustering based on pairwise identity by state distance in PLINK^56^ showed no batch effects, therefore the batches were merged. Standard genotype data quality control was performed as described previously^48^. Briefly, individuals were excluded if: >3% of SNP data were missing (*n* = 11), reported sex did not match genotyped sex (*n* = 48), duplicates (*n* = 123), missing phenotype data (*n* = 11), or >5 standard deviations above/below mean heterozygosity (*n* = 28). Individuals with non-European ancestry (*n* = 4) were also excluded. To prepare genotype data for imputation, SNPs were excluded if: call rates <95%, minor allele count <10, deviations from HWE (*P* < 5.0 × 10^−4^), no matching Haplotype Reference Consortium (HRC) reference panel SNP, palindromic (A/T, G/C) SNPs with MAF greater than 0.4 from the HRC (*n* = 5), and SNPs with >0.2 MAF difference compared to HRC (*n* = 150). After quality control, SNP data was available for 513,634 SNPs. Imputation was performed to the HRC reference panel using the Michigan Imputation Server^57^. Following imputation, 39,117,105 SNPs were available for analysis. We excluded variants if the number of copies of the minor allele <5 or if imputation quality (*r*^*2*^) <0.3. This resulted in 13,887,524 variants available for analysis.

Genotyping in ADNI was performed on the Human 610-Quad BeadChip (Illumina, Inc., San Diego, CA). Following standard quality control procedures performed in Plink^56^ (minimum SNP and individual call rate >95%, MAF > 0.05, HWE test *P* > 1 × 10^−6^), the sample was imputed to the 1000 Genomes Phase 3 reference panel using Impute2^58^, with pre-phasing using ShapeIT^59^.

Genotyping in AIBL was performed on the Infinium OmniExpressExome array (Illumina, Inc., San Diego, CA)^60^. Quality control procedures were performed in Plink^56^. After removing individuals with ambiguous sex, Plink was used to remove individuals with call rate <0.90; SNPs were removed if call rate <0.95, HWE test *P* < 1.0 × 10^−4^, or MAF < 0.05. SNPs were flipped to the positive strand before imputation to the 1000 Genomes Phase 3 reference panel using the Michigan Imputation Server^57^ (using Minimac 4). Both the AIBL and ADNI validation cohorts were restricted to individuals of non-Hispanic European ancestry, based on projection onto the 1000 Genomes reference panel.

### Genetic relatedness matrix

The discovery sample, BHS, used in this study consisted of related and unrelated individuals; therefore, all analyses included a genetic relatedness matrix. Twenty-two genetic relatedness matrices were calculated. First, a hard-call set of imputed SNPs was created in Plink (i.e. SNP genotypes were called if SNP imputation quality *r*^*2*^ > 0.8 and if genotype probability >0.9). The *HLA* region on chromosome 6 was also excluded. SNPs were then pruned in Plink using ‘indep-pairwise 500 50 0.3’ [window of size 500, moving 50 SNPs along each time, removing variants with *r*^*2*^ > 0.3] to create a set of 486,553 independent SNPs. Twenty-two genetic relatedness matrices were created (using the option ‘gk 1’ which specifies a centred relatedness matrix), with each omitting one chromosome, in GEMMA^61^.

### Statistical analysis

Genome-wide association analyses for the 596 lipid species and 33 lipid classes in the discovery cohort were performed using imputed genotype dosages in linear mixed models, as implemented in GEMMA^61^. To avoid proximal contamination, analyses were performed using genetic relatedness matrices implementing a leave-one-chromosome out scheme. Analyses were performed using rank-based inverse normal transformed residuals, after adjustment by age, sex, age^2^, age*sex, age^2^*sex, and the first 10 principal components (generated from Eigenstrat)^62,63^.

Validation cohorts, ADNI and AIBL, were analysed using an additive linear model, as implemented in Plink^56^. Analyses were performed using rank-based inverse normal transformed residuals, after adjustment by age, sex, age^2^, age*sex, age^2^*sex, study-specific covariates (including fasting status for ADNI) and a number of principal components deemed sufficient to capture population structure. Meta-analysis between all three studies was performed using an inverse-variance weighted fixed-effects model, as implemented in METAL^64^. Due to the correlation between lipid species, the effective number of tests was calculated as the number of principal components required to explain at least 95% variance of the lipidome (144 components).

Statistical significance was defined using the standard genome-wide significance (*P* < 5 × 10^−8^) in the BHS discovery analysis, *P* < 0.05 in AIBL/ADNI validation, and *P* < 3.47 × 10^−10^ in the three-study meta-analysis (5 × 10^−8^/144 lipid dimensions; Bonferroni correction using the effective number of tests). A more stringent threshold was used for the meta-analysis due to the lack of validation samples available.

For each lipid, significantly associated SNPs were LD-clumped (*r*^*2*^ > 0.1) using correlation measures obtained from 10,000 unrelated individuals from the UK Biobank, the 1000 Genomes, or the BHS. A singular dataset was created by retrieving the smallest *P*-value across all analyses. This dataset was LD-clumped (*r*^*2*^ > 0.1) to determine the number of independent genomic regions. For each locus, a regional association plot was produced using LocusZoom^65^.

### Detection of distinct association signals

Conditional analysis was performed to detect independent association signals at each genome-wide significant loci using GEMMA. For each lipid, we iteratively clumped regions within a 2 Mb window centred on the lead SNP until no more genome-wide significant associations were left. Regions with overlapping windows were merged. Conditional analysis was iteratively performed, including the lead variant as a covariate until no more conditionally independent signals (*P* < 5 × 10^−8^) remained.

### Assessment of effects of clinical lipid trait adjustment

Within the discovery cohort, to determine whether SNP-lipid associations were independent of clinical lipid traits (total cholesterol, HDL-cholesterol, triglycerides), all SNPs were tested with and without adjustment for clinical lipid traits. We compared loci effect sizes between analyses run with and without clinical lipid adjustment using a pooled standard deviation *t*-test (Supplementary Note 3). Bonferroni adjustment (0.05/number of loci) was used to identify loci which differed substantially following adjustment. As adjusting for heritable covariates can introduce collider bias^66^, we further validated these using multi-trait conditional and joint analysis (mtCOJO)^67^, conditioning on GWAS summary-level data for clinical lipids obtained from the UK Biobank^68^.

### Annotation

Proxies for lead SNPs were found by identifying those in high LD (*r*^*2*^ > 0.8) within the BHS dataset; in an unrelated subset of white, British individuals from the UK Biobank^69^; or in the 1000 Genomes. Lead SNPs and their proxies were annotated using SNPEff^70^. SNiPA database v3.3^71^ was used to retrieve the combined annotation dependent depletion (CADD) score. Expression QTL associations (cis-eQTL) were obtained from GTEx^72^ (release v8) and eQTLGen^73^ (release 2019-12-20). SNiPA metabolite QTL (mQTL) associations were supplemented with mQTL associations reported in PhenoScanner^74,75^ and recently published lipidomic GWAS^7,17^. SNiPA protein QTL (pQTL) associations were supplemented with cis-pQTL associations from ref. ^76^. Methylation QTL (meQTL) associations were obtained from ref. ^77^. A locus was defined as previously unreported if the lead SNP or its proxies have not been identified as an mQTL or lipid-related trait loci.

Putative causal genes, for each loci, were identified using a slightly modified approach to that previously described (ProGeM)^22^. For the bottom-up approach, the three closest protein coding genes (within a 1 Mb window) were identified, for each lead SNP. Genes were noted if a lead SNP or its proxies were annotated by SNPEff as missense, start loss, stop gain, or with an annotation impact as High. As performed by ProGeM, the top-down analysis reports genes within 500 kb of the lead SNP that are present in a curated database of known metabolic-related genes. A list of primary candidates was generated based on the overlap of top-down and bottom-up genes.

### Overlap of lead variants with cardiovascular disease-related loci

To assess whether our lead SNPs were previously associated with CVD-related traits, we performed a look-up within the GWAS Catalog v1.02 (release 2020-08-26)^78^ of 10 hard CVD endpoints, 72 CVD-related traits, and 141 lipid-related traits. We also performed a look-up against a meta-analysis of CAD between CARDIoGRAMplusC4D and UK Biobank^79^.

### Associations of lipid species with coronary artery disease and coronary artery disease polygenic risk

Within the discovery cohort, the association of lipid species with incident CAD was assessed using logistic regression, adjusting for age, sex, and the first 10 genomic principal components. Prevalent CAD cases were removed prior to analysis; defined as individuals hospitalised with CAD between the start of the Hospital Morbidity Data Collection (1970), and an individual’s serum collection date. Incident CAD events (CAD hospitalisations or death) were included up to the end of follow-up (July 2015). Results are displayed as log-odds ratios.

Polygenic risk for CAD was calculated for each individual in the discovery cohort using the metaGRS polygenic score, consisting of ~1.7 million genetic variants^23^. Linear regression in R was performed to test the association between an individual’s polygenic score and lipid species concentrations, adjusting for age, sex, and the 10 first principal components.

### Genetic correlations

Genetic correlations of lipid species against CAD were assessed using Linkage Disequilibrium Score Regression (v1.0.1)^80^. Regression weights and scores were obtained from 1000 Genomes European data, as previously described^81^. Summary statistics from all datasets were restricted to SNPs from the HapMap 3 panel, with 1000 Genomes European MAF greater than 5%. Where available, SNPs were filtered to an imputation quality *r*^*2*^ > 0.9. Similarly, SNPs were removed if the reported MAF deviated from 1000 Genomes European MAF by greater than 0.1. Summary statistics for CAD were obtained from the meta-analysis of CARDIoGRAMplusC4D and UK Biobank by van der Harst and Verweij^79^. Due to no overlapping samples between BHS and other summary results, the genetic covariance intercept was constrained to 0.

### Co-localisation analysis

Co-localisation between lipid species genome-wide significant loci and CAD was performed using the R package COLOC^82^. For each loci, all variants within a 400 kb window centred on the lead SNP were selected. Priors were kept at default settings. Evidence for shared variants was determined as the posterior probability of both traits containing causal variants in the region (H3 + H4 > 0.8) and a larger probability of a shared variant (H4/H3 > 10). Sensitivity analysis for regions with shared variants is shown in Supplementary Note 1.

### Association of loci with coronary atherosclerosis in the UK Biobank

Lead SNPs (or proxies) were tested for association with coronary atherosclerosis in the UK Biobank. In a subset of white, British individuals (*n* = 456,486), electronic health records (updated 14^th^ December 2020) were converted into PheCodes^83,84^ using the R package PheWAS^85^. Coronary atherosclerosis (phecode 411.4) was exported for genome-wide association analysis. FastGWA^86^ was used to assess the association of lipid-loci with these phenotypes, adjusting for age, sex, age^2^, age*sex, age^2^*sex, the first 20 principal components as provided by the UK Biobank, and the genetic relatedness matrix as the random effect. The analysis was repeated, additionally adjusting for clinical lipids (total cholesterol, HDL-cholesterol, triglycerides; measurements obtained from the first available blood collection). Individuals with missing values were excluded from the analysis. As clinical lipids are heritable, mtCOJO analysis was also performed using GWAS summary statistics obtained above.

### Reporting summary

Further information on research design is available in the Nature Research Reporting Summary linked to this article.

## Supplementary information

Supplementary Information

Description of Additional Supplementary Files

Supplementary Dataset 1

Supplementary Dataset 2

Supplementary Dataset 3

Supplementary Dataset 4

Supplementary Dataset 5

Supplementary Dataset 6

Supplementary Dataset 7

Supplementary Dataset 8

Supplementary Dataset 9

Supplementary Dataset 10

Supplementary Dataset 11

Supplementary Dataset 12

Supplementary Dataset 13

Supplementary Dataset 14

Supplementary Dataset 15

Supplementary Dataset 16

Supplementary Dataset 17

Reporting Summary

## Data Availability

Complete summary statistics of all lipid species and classes are available via the NHGRI-EBI GWAS Catalog (https://www.ebi.ac.uk/gwas), GCP ID: GCP000197; study accession nos. GCST90023981–GCST90025848. In addition, summary-level statistics are available at our data portal (https://metabolomics.baker.edu.au/). Source data are provided with this paper. Individual-level data for the BHS are available under restricted access for bona fide research; access can be obtained through applications to the Busselton Population Medical Research Institute (http://bpmri.org.au/research/database-access.html). Individual-level data for the ADNI and AIBL studies are available under restricted access for bona fide research; access can be obtained through applications to the LONI Image and Data Archive (http://adni.loni.usc.edu/data-samples/access-data/). Individual-level data for AIBL are also available through applications to the AIBL management committee (https://aibl.csiro.au/research/support/). Publicly available datasets used within the study are available via UK Biobank (http://www.ukbiobank.ac.uk/register-apply/), HRC (http://www.haplotype-reference-consortium.org/home), 1000 Genomes (https://www.internationalgenome.org/), SNiPA (https://snipa.helmholtz-muenchen.de/snipa3/), GTEx (https://gtexportal.org/home/), and eQTLGen (https://www.eqtlgen.org/). Source data are provided with this paper.

## Source data

Source Data

## Code availability

All software and bioinformatic tools used in the present study are publicly available.
