# Supplementary material for: Comprehensive genetic analysis of the human lipidome identifies loci associated with lipid homeostasis with links to coronary artery disease
Source: Nat Commun. 2022 Jun 6;13:3124. doi: 10.1038/s41467-022-30875-7 (PMC9170690; doi:10.1038/s41467-022-30875-7)
Supplement: Supplementary file 1 — Supplementary Information [file 41467_2022_30875_MOESM1_ESM.pdf]

Comprehensive genetic analysis of the human lipidome identifies loci controlling lipid homeostasis with links to coronary artery disease

Gemma Cadby[1,*], Corey Giles[2,3,*], Phillip E Melton[1,4], Kevin Huynh[2,3], Natalie A Mellett[2], Thy Duong[2], Anh Nguyen[2], Michelle Cinel[2], Alex Smith[2], Gavriel Olshansky[2,3], Tingting Wang[2,3], Marta Brozynska[2], Mike Inouye[2], Nina S McCarthy[5], Amir Ariff[6], Joseph Hung[7,8,9], Jennie Hui[9,10], John Beilby[9,10], Marie-Pierre Dubé[11], Gerald F Watts[7,12], Sonia Shah[13], Naomi R Wray[13,14], Wei Ling Florence Lim[15,16], Pratishtha Chatterjee[15,17,18], Ian Martins[15], Simon M Laws[19,20,21], Tenielle Porter[19,20,21], Michael Vacher[19,20,22], Ashley I Bush[23], Christopher C Rowe[23,24], Victor L Villemagne[24,25], David Ames[26,27], Colin L Masters[23], Kevin Taddei[15], Matthias Arnold[28,29], Gabi Kastenmüller[29], Kwangsik Nho[30,31,32], Andrew J Saykin[30,32,33], Xianlin Han[34], Rima Kaddurah-Daouk[28,35,36], Ralph N Martins[15,16,17,18], John Blangero[37], Peter J Meikle[2,3,38,**], Eric K Moses[4,5,**].

*These authors contributed equally; ** These authors jointly supervised this work

Supplementary Material

Supplementary Table 1	---	3
Supplementary Table 2	---	4
Supplementary Figure 1	---	5
Supplementary Figure 2	---	6
Supplementary Figure 3	---	7
Supplementary Figure 4	---	8
Supplementary Note 1	---	9
Supplementary Note 2	---	53
Supplementary Note 3	---	54
Supplementary References	---	55

Supplementary Table 1: Basic characteristic of BHS, ADNI, and AIBL cohorts

**Busselton Health Study (BHS)**	**Male**	**Female**	**Overall**
n (%)	1976 (44.0)	2516 (56.0)	4492
Age (Years)	50.9 (17.4)	50.8 (17.3)	50.8 (17.4)
BMI (kg/m^2)	26.5 (3.5)	25.6 (4.6)	26.0 (4.2)
Cholesterol (mmol/L)	5.52 (1.09)	5.61 (1.17)	5.57 (1.14)
HDL-C (mmol/L)	1.20 (0.32)	1.53 (0.40)	1.39 (0.40)
Triglycerides (mmol/L)	1.46 (1.10)	1.19 (0.73)	1.31 (0.92)
Lipid lowering medication use[a]	53 (2.7)	55 (2.2)	108 (2.4)
Lifetime CAD events[b]	425 (21.5)	364 (14.5)	789 (17.6)
Incident CAD (post collection)	275 (13.9)	276 (11.0)	551 (12.3)
**Alzheimer's Disease Neuroimaging Initiative (ADNI)**	**Male**	**Female**	**Overall**
n (%)	267 (39.9)	403 (60.1)	670
Age (Years)	74.7 (6.6)	75.8 (6.7)	75.3 (6.7)
BMI (kg/m^2)	25.2 (4.8)	26.5 (4.0)	26.0 (4.4)
Cholesterol (mmol/L)	5.20 (1.04)	4.50 (0.93)	4.78 (1.03)
HDL-C (mmol/L)	1.53 (0.48)	1.25 (0.38)	1.36 (0.44)
Triglycerides (mmol/L)	1.54 (1.32)	1.34 (0.76)	1.42 (1.02)
Lipid lowering medication use[c]	119 (44.6)	209 (51.9)	328 (49.0)
Non fasting status	23 (8.6)	39 (9.7)	62 (9.3)
**Australian Imaging, Biomarker & Lifestyle Study of Ageing (AIBL)**	**Male**	**Female**	**Overall**
n (%)	510 (57.0)	385 (43.0)	895
Age (Years)	74.4 (7.7)	75.1 (7.0)	74.7 (7.4)
BMI (kg/m^2)	26.0 (4.3)	26.3 (3.3)	26.2 (3.9)
Cholesterol (mmol/L)	5.64 (0.90)	5.01 (0.93)	5.37 (0.96)
HDL-C (mmol/L)	1.77 (0.39)	1.42 (0.33)	1.62 (0.41)
Triglycerides (mmol/L)	1.34 (0.58)	1.29 (0.58)	1.32 (0.58)
Lipid lowering medication use[d]	115 (22.5)	83 (21.7)	198 (22.1)

Continuous variables presented as mean (standard deviation); categorial variables presented as count (percentage)

[a] Lipid lowering medication in BHS: 85 Lipex/Zocor (simvastatin), 21 Lopid (gemfibrozil), 4 Pravachol (pravastatin), 3 Questran Lite (cholestyramine), 1 nicotonic acid.

[b] Coronary artery disease (CAD) events defined as either hospitalisation or death due to coronary artery disease (ICD9: 410-414; ICD: I20-I25)

[c] Lipid lowering medication in ADNI: 166 Lipitor (atorvastatin), 90 Lipex/Zocor (simvastatin), 40 Lovastatin, 20 Vytorin (ezetimibe/simvastatin), 12 miscellaneous.

[d] Lipid lowering medication in AIBL: 117 Lipitor (atorvastatin), 55 Lipex/Zocor (simvastatin), 13 Pravachol (pravastatin), 13 miscellaneous.

Supplementary Table 2: Associations between 737 lead SNPs with lipid species and metabolites in previous studies. We identified 35 previous studies that had reported one or more of our 737 lead SNPs or a proxy (r^2>0.8) (Supplementary Data 12); details below.

Author/database	PMID	Sample size	Method	# lipids/metabolites	Ethnicity	Reference
**Burkhardt, R.**	26401656	2,107	MS	96 AA, AC	Central European	1
**Chai, J.F.**	31628463	1,954	MS/MS	136 AA, AC	Chinese	2
**Chasman, D.I.**	19936222	17,296	NMR	17 lipoproteins	European	3
**Davis, J.**	29084231	8,372	NMR	68 lipid and lipoprotein subclasses	Finnish	4
**Demirkan, A.**	22359512	4,034	ESI-MS/MS	153 lipids	European	5
**Draisma, H.**	26068415	7,478+1182	ESI-FIA-MS/MS	129 metabolites	European	6
**Feofanova, E.**	29610217	1,552+1,872	GC-MS, LC-MS	102 circulating lipid-related metabolites	European, African-American	7
**Gieger, C.**	19043545	284	ESI-MS/MS	363 metabolites	Southern German	8
**Harshfield, E.**	34503513	5,662+13,814	DI-HRMS	360 lipids	Pakistani, European	9
**Hartiala, J.**	26822151	1,985/1,895	ESI-MS/MS	plasma betaine levels	European	10
**Hicks, A.A.**	19798445	4,400	ESI-MS/MS	33 sphingolipids, and 43 matched metabolite ratios	European	11
**Hong, M.G.**	23281178	402/489	UHPLC-MS	6,138 unique molecular features	Swedish	12
**Hu, Y.**	28298293	3,521+12,020	TLC-GC	Unclear number of Monounsaturated FA	Chinese, European	13
**Illig, T.**	20037589	1,809/422	HPLC-ESI-MS/MS	163 metabolic traits	European	14
**Inouye, M.**	22916037	1,905+4703	NMR	130 metabolite measures	European	15
**Kalsbeek, A.**	29652918	2,400	GC	22 FA and 15 FA ratios	Framingham Heart Study Offspring Cohort	16
**Kettunen, J.**	22286219	8,330	NMR	216 serum metabolic traits	Finnish	17
**Kettunen, J.**	27005778	24,925	NMR	123 metabolic traits	European	18
**Krumsiek, J.**	23093944	1,768	UHPLC-MS/MS, GC-MS/MS	517 metabolic traits (including 225 unknowns)	German	19
**Long, T.**	28263315	1,960	UHPLC–MS/MS	644 metabolites	European	20
**Lotta, L.**	27898682	16,596	MS/MS, GC-MS, UHPLC-MS/MS	Branched-chain AA	European	21
**Lotta, L.**	33414548	8,569 - 86,507	FIA--MS/MS, UHPLC-MS, NMR	174 metabolites	European	22
**Mittelstrass, K.**	21852955	3,381	HPLC-MS/MS	131 metabolites	South German	23
**Nicholson, G.**	21931564	211	NMR, FIA-ESI-MS/MS	526 metabolite peaks	European	24
**Raffler, J.**	26352407	3,861+1,691	NMR	15,379 targeted and untargeted metabolic traits	European	25
**Rhee, E.**	23823483	2,076	HPLC-ESI-MS/MS	217 metabolites	European	26
**Shin, S.**	24816252	7,824	HPLC-MS/MS, GC-MS/MS	486 metabolites (including 177 unknown)	European	27
**SNIPA**	25431330	-	-	-	Various	28
**Suhre, K.**	21886157	1,768+1,052	UHPLC-MS/MS, GC-MS/MS	276 metabolites	European	29
**Tabassum, R.**	31551469	2,181	DI-HRMS	141 lipids	European	30
**Teslovich, T.**	29481666	8,545	NMR	9 AA	Finnish	31
**Tukiainen, T.**	22156771	8,330	NMR	117 metabolites	Finnish	32
**Xie, W.**	23378610	957+341	Unclear	14 metabolites	European	33
**Yet, I.**	27073872	1,001	MS	648 metabolites	European	34
**Yu, B.**	27884205	1,872+1,552	GC-MS, LC-MS	70 AA	African American, European American	35

Mass-spectrometry, MS; Tandem mass-spectrometry, MS/MS; High-resolution mass-spectrometry, HRMS; Nuclear magnetic resonance, NMR; Electrospray ionization, ESI; Gas chromatography, GC; (Ultra-)High performance liquid chromatography, (U)HPLC; Thin-layer chromatography, TLC; Flow-injection/infusion analysis, FIA; Direct infusion, DI; Amino acids, AA; Acyl-carnitines, AC; Fatty acids, FA;

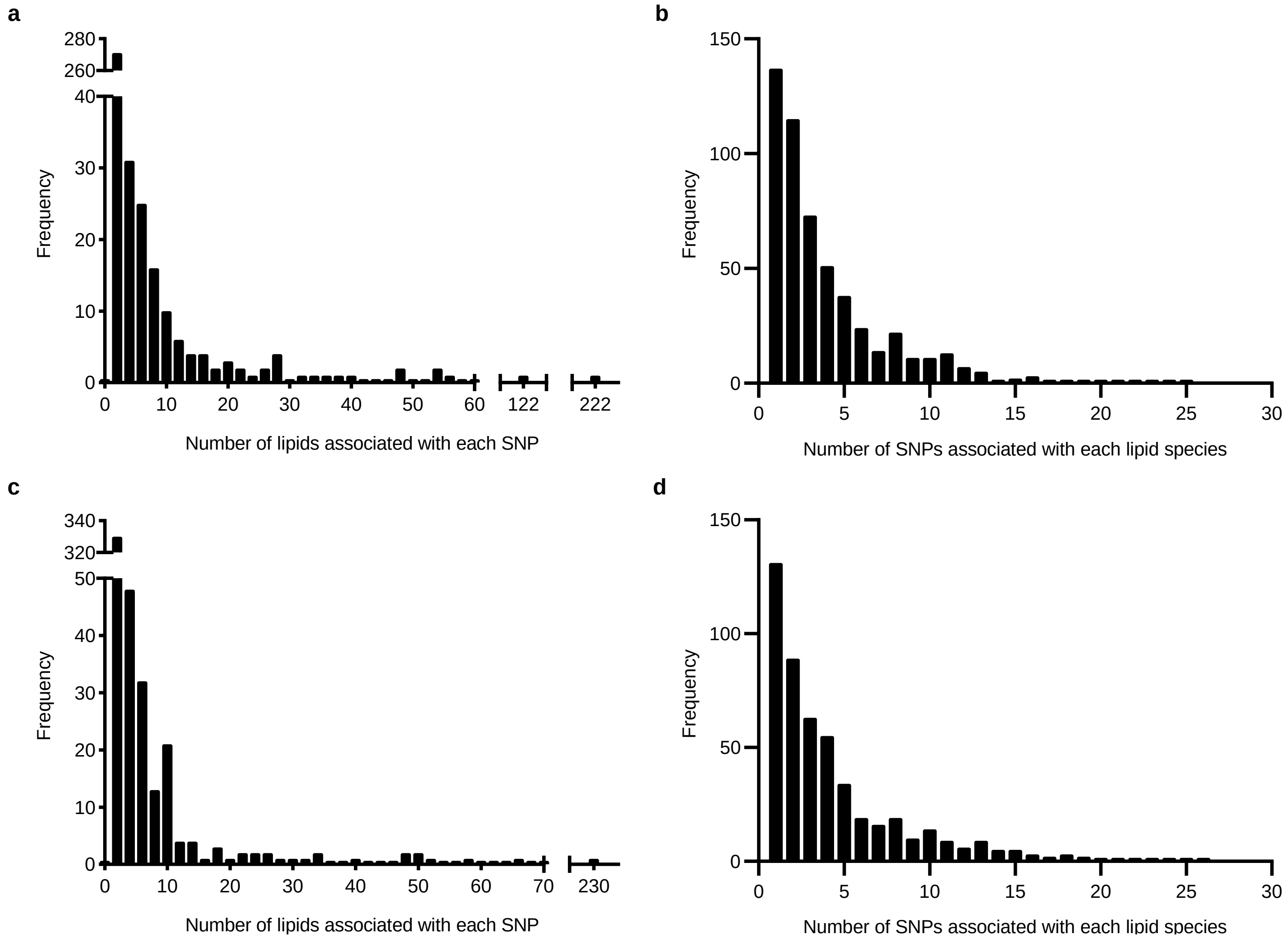

Supplementary Figure 1: Distribution of genome-wide significant associations for independent SNPs and lipid species. **a**, the number of lipid species associated with independent SNPs in the BHS discovery cohort. **b**, the number of independent SNPs associated with each lipid species in the BHS discovery cohort. **c**, the number of lipid species associated with independent SNPs in the clinical lipid adjusted discovery GWAS. **d**, the number of independent SNPs associated with each lipid species in the clinical lipid adjusted discovery GWAS.

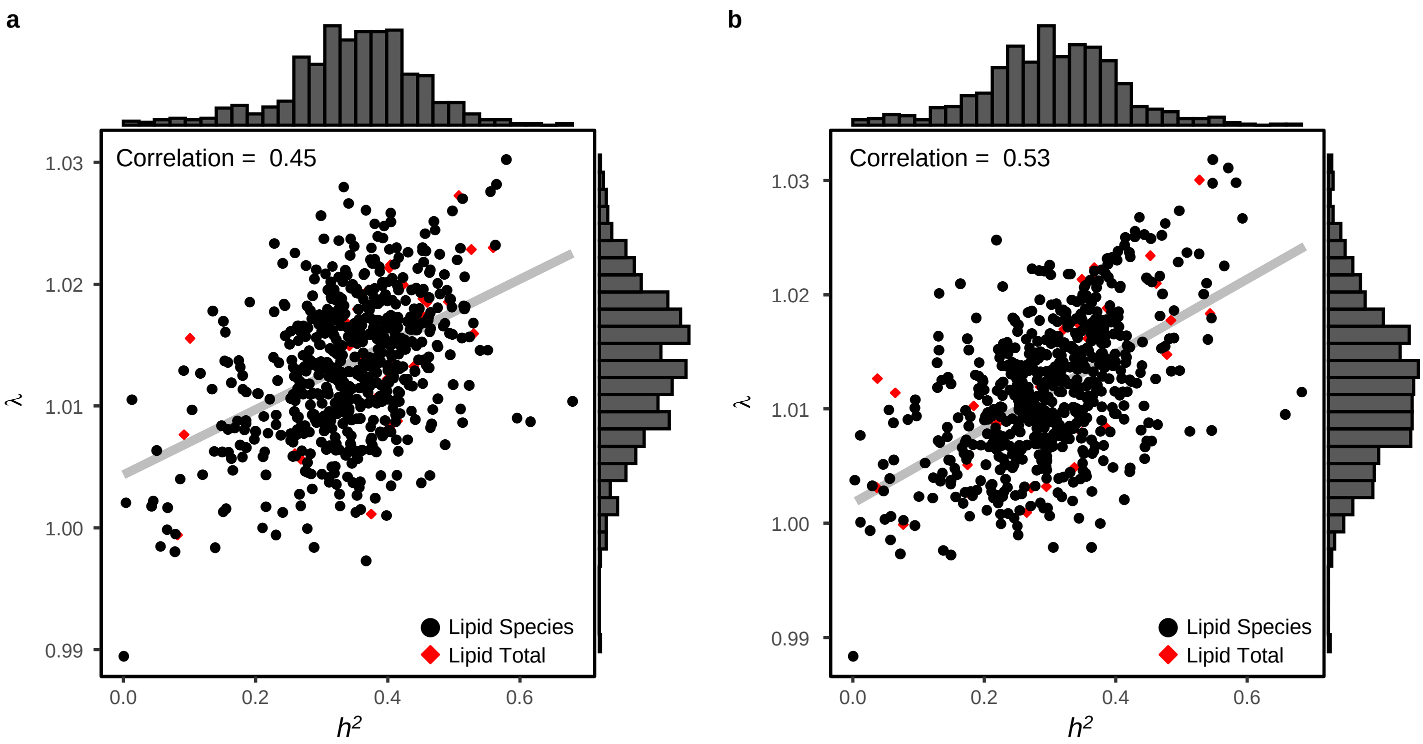

Supplementary Figure 2: Scatterplot of lipid heritabilities (*h^2*) vs GWAS genomic inflation factors (λ) for lipid species and classes. **a**, lipid heritability and genomic inflation factors for genome-wide association analysis in the BHS cohort. **b**, lipid heritability and genomic inflation factors for genome-wide association analysis, adjusting for clinical lipids, in the BHS cohort. Red diamonds indicate lipid classes and black circles indicate lipid species. The correlation between the heritabilities and genomic inflation factors are also shown, with a line of best fit. The right and top axes show histograms of the distribution of the genomic inflation factors from each GWAS, and heritability estimates, respectively. Heritability estimates were calculated in GCTA; using the genetic related matrix (GRM) and adjusted by age, sex, age^2, age*sex, age^2*sex.

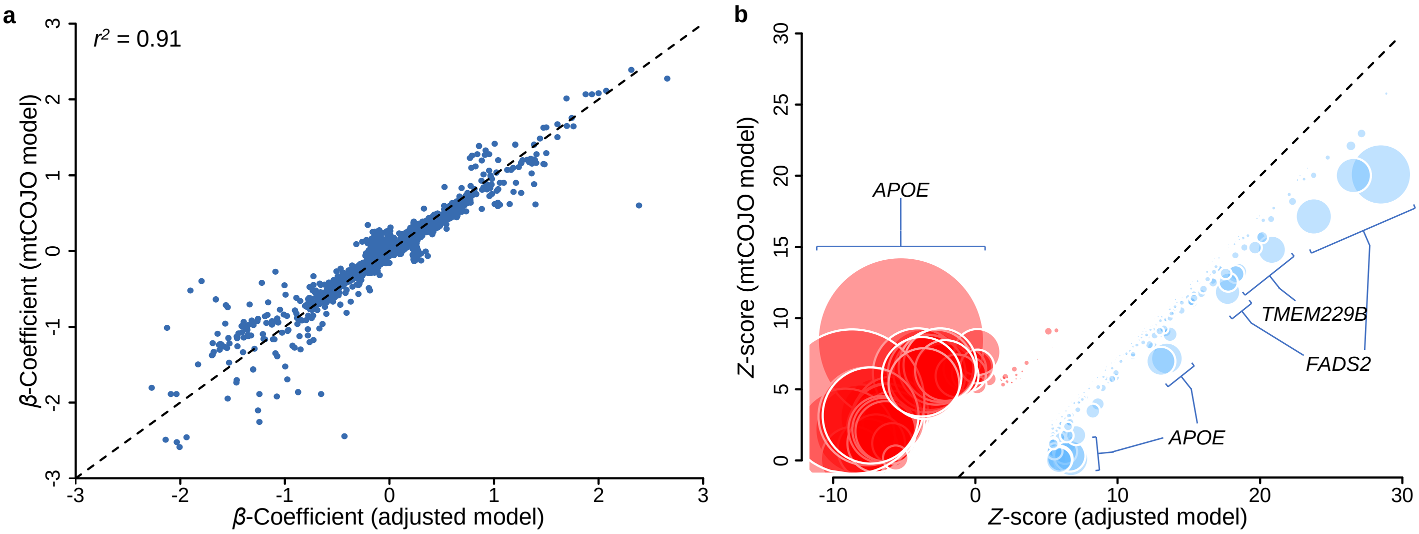

Supplementary Figure 3: Comparison of estimated lipidomic effect sizes between clinical lipid adjusted and mtCOJO adjusted models. **a**, Beta coefficients for clinical lipid adjusted SNP-lipid associations (x axis) are plotted against mtCOJO adjusted SNP-lipid associations (y axis). **b**, *Z*-scores (Beta coefficient divided by standard error) for clinical lipid adjusted SNP-lipid associations (x axis) are plotted against mtCOJO adjusted SNP-lipid associations (y axis). Variant effect signs are fixed so mtCOJO adjusted associations are positive. Variants showed greater (positive) associations in mtCOJO adjusted analysis are shown in red, and variants showing reduced associations are shown in blue. Circle diameter is proportional of $-\log_{10}(P)$ *t*-test of effect differences.

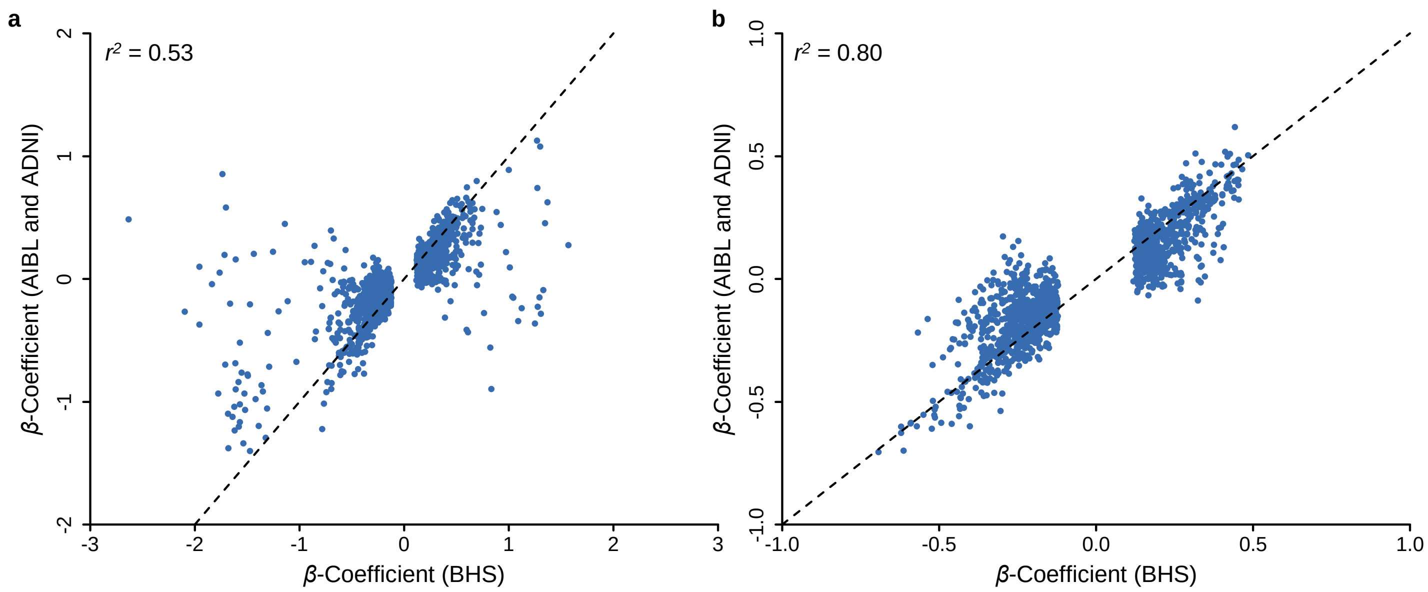

Supplementary Figure 4: Comparison of estimated lipidomic effect sizes between the BHS discovery GWAS and the validation meta-analysis (ADNI and AIBL). **a**, Beta coefficients were estimated from linear regression models for lipid species using the Busselton Health Study discovery GWAS (x-axis) and the ADNI and AIBL validation meta-analysis (y-axis). **b**, Beta coefficients for only common SNPs (MAF>=0.05) in the Busselton Health Study discovery GWAS (x-axis) and the ADNI and AIBL validation meta-analysis (y-axis). Only significantly associated SNPs ($P<5x10^{-8}$) in the Busselton Health Study discovery GWAS are shown.

Supplementary Note 1: Regional association plots for lipid loci that colocalize with coronary artery disease. We identified 43 putative shared causal variants from colocalization analysis of lipid loci (Supplementary Data 16). Supplementary Figures 5-47 show the regional association plot of coronary artery disease[36] and up to the top four lipid species that show evidence of colocalization (H3+H4 > 0.8; H4/H3 > 10). Regions were isolated from the 737 lead SNPs, with a window of 400 KB centred on the lead SNP. The indicated candidate SNP was the genetic variant with the largest posterior probability. Marker colours indicate linkage disequilibrium with the candidate SNP (obtained from the 1000 Genome European dataset). Colocalization sensitivity analysis was performed for the top colocalization, assessing the posterior probability of H0-H4 for different values of prior p_{12}. Shaded regions indicate the value of p_{12} which produces posterior probabilities of H3+H4 > 0.8 and H4/H3 > 10.

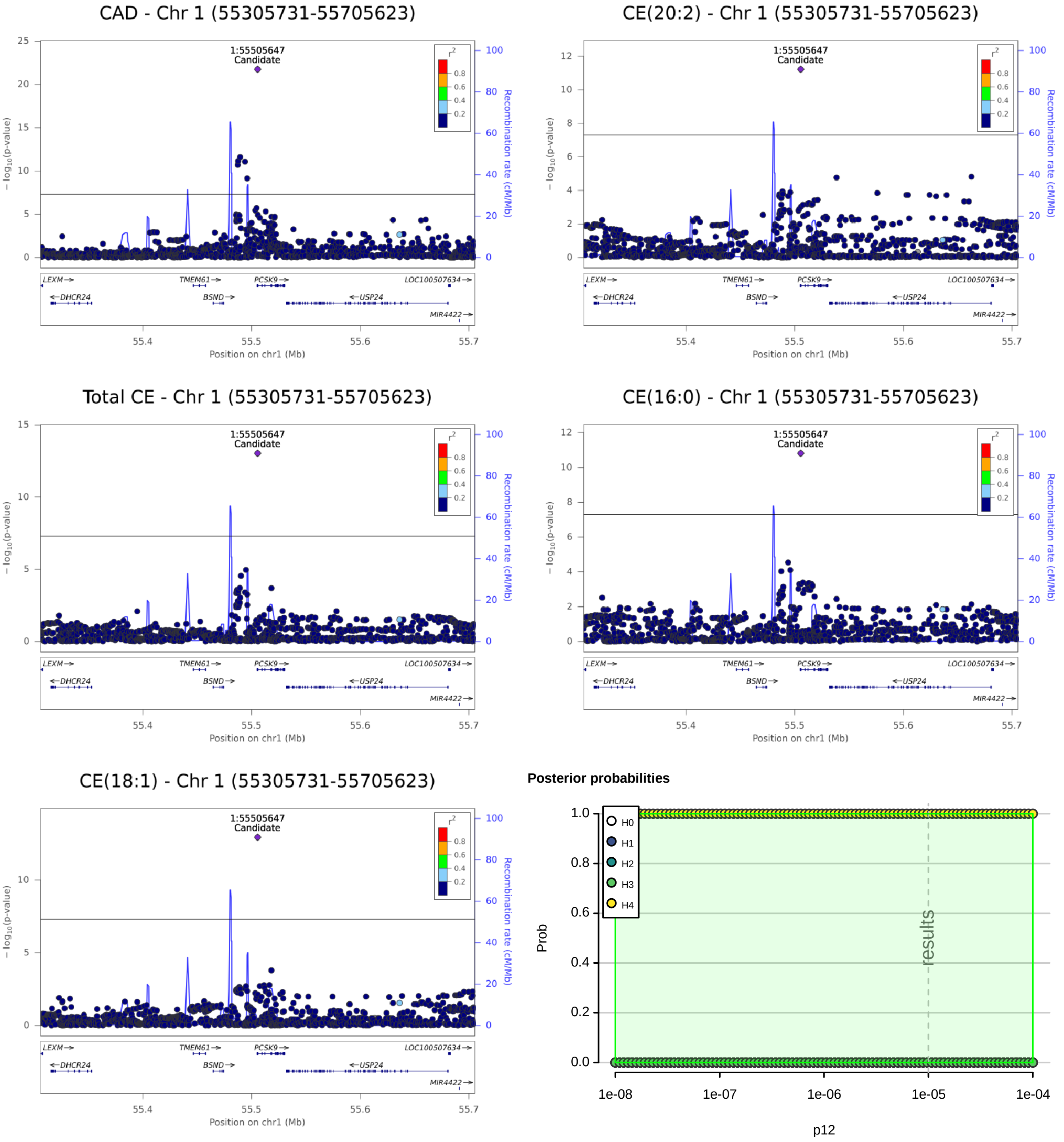

Supplementary Figure 5: Regional association plot of Chr1:55305731-55705623 and coronary artery disease and the top four lipid species that show evidence of colocalization (H3+H4 > 0.8; H4/H3 > 10), along with colocalization sensitivity analysis for the top colocalization, assessing the posterior probability of H0-H4 for different values of prior p_{12}.

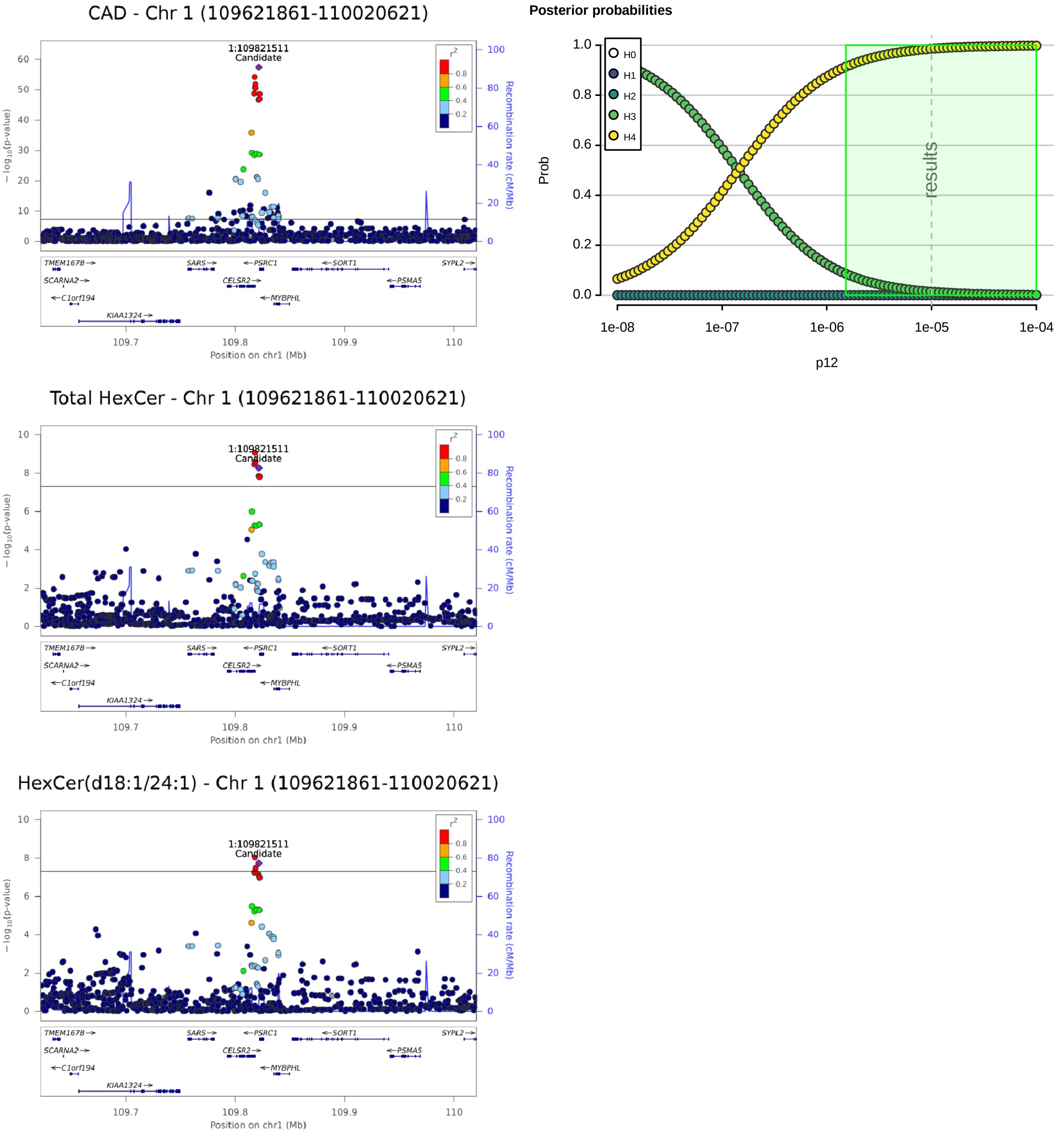

Supplementary Figure 6: Regional association plot of Chr1:109621861-110020621 and coronary artery disease and the top two lipid species that show evidence of colocalization (H3+H4 > 0.8; H4/H3 > 10), along with colocalization sensitivity analysis for the top colocalization, assessing the posterior probability of H0-H4 for different values of prior p_{12}.

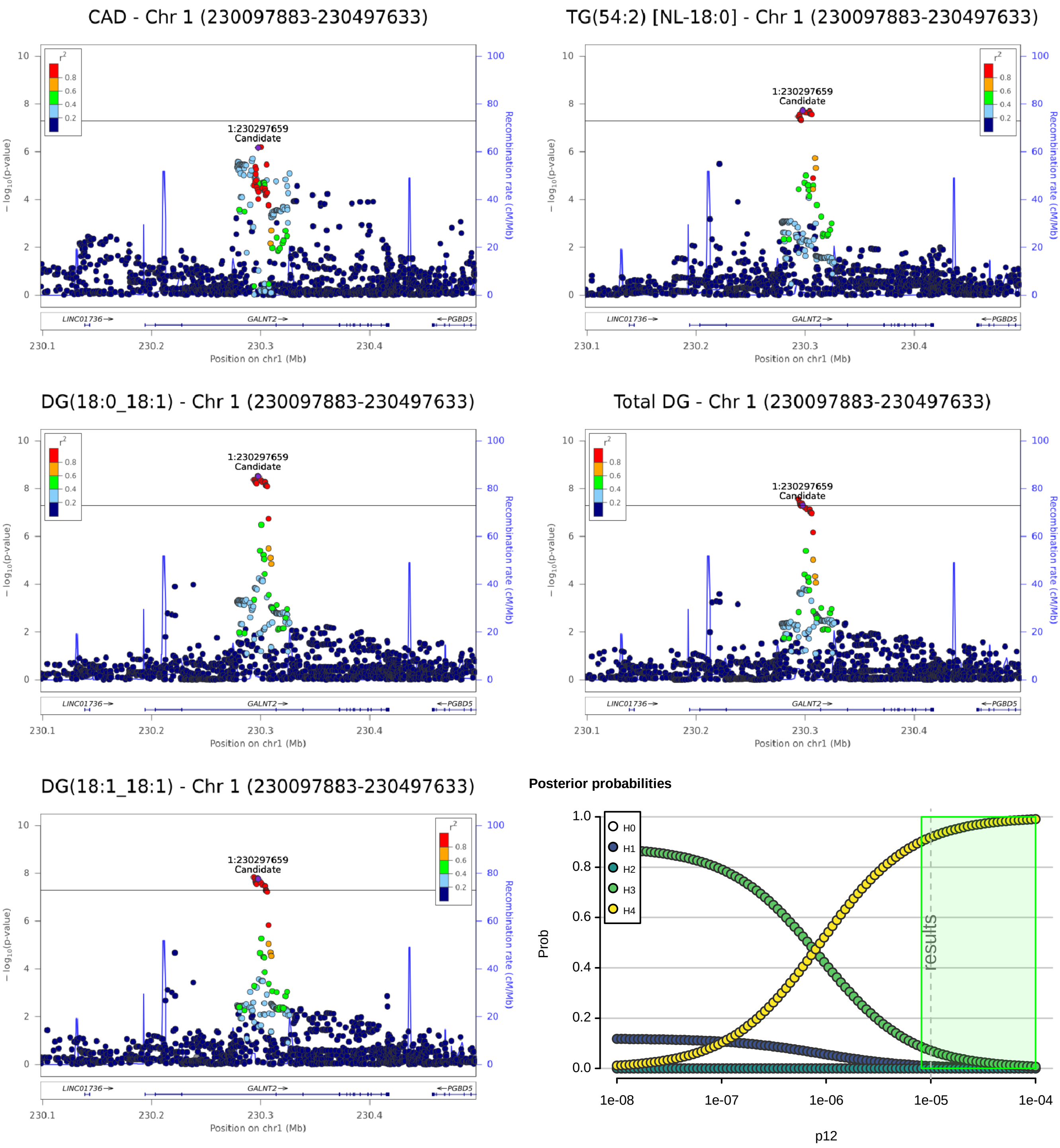

Supplementary Figure 7: Regional association plot of Chr1:230097883-230497633 and coronary artery disease and the top four lipid species that show evidence of colocalization (H3+H4 > 0.8; H4/H3 > 10), along with colocalization sensitivity analysis for the top colocalization, assessing the posterior probability of H0-H4 for different values of prior p_{12}.

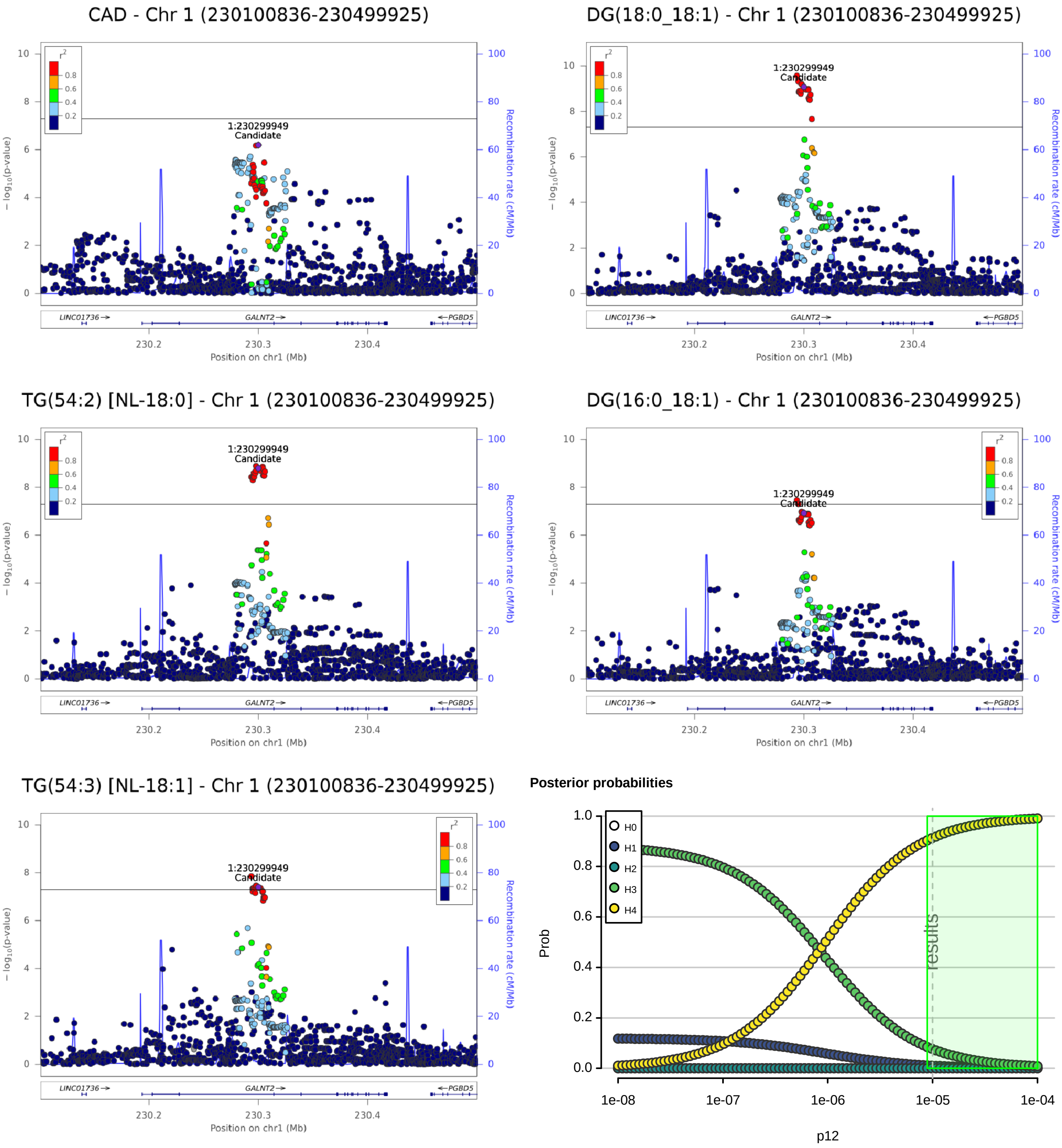

Supplementary Figure 8: Regional association plot of Chr1:230100836-230499925 and coronary artery disease and the top four lipid species that show evidence of colocalization (H3+H4 > 0.8; H4/H3 > 10), along with colocalization sensitivity analysis for the top colocalization, assessing the posterior probability of H0-H4 for different values of prior p_{12}.

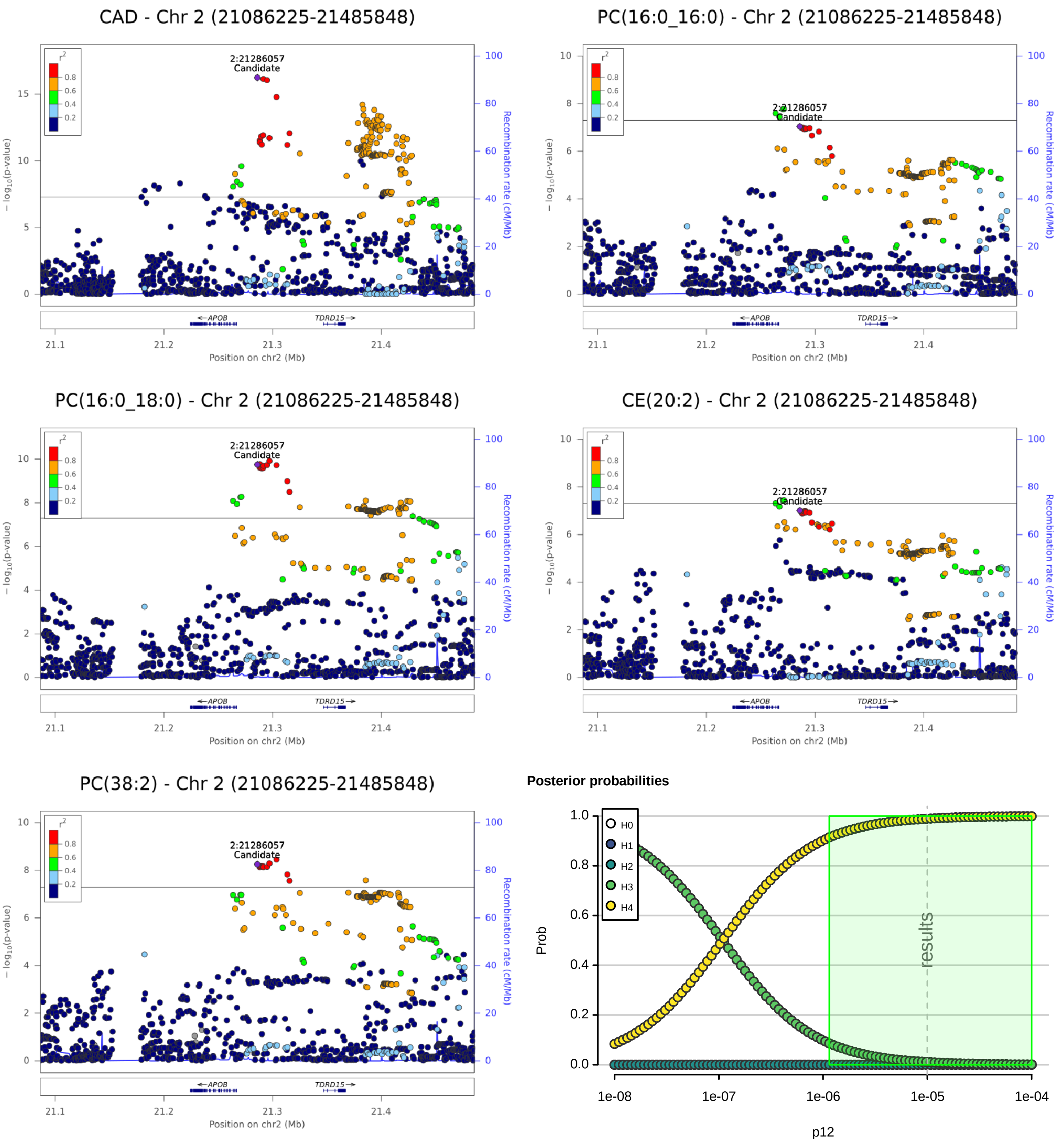

Supplementary Figure 9: Regional association plot of Chr2:21086225-21485848 and coronary artery disease and the top four lipid species that show evidence of colocalization (H3+H4 > 0.8; H4/H3 > 10), along with colocalization sensitivity analysis for the top colocalization, assessing the posterior probability of H0-H4 for different values of prior p_{12}.

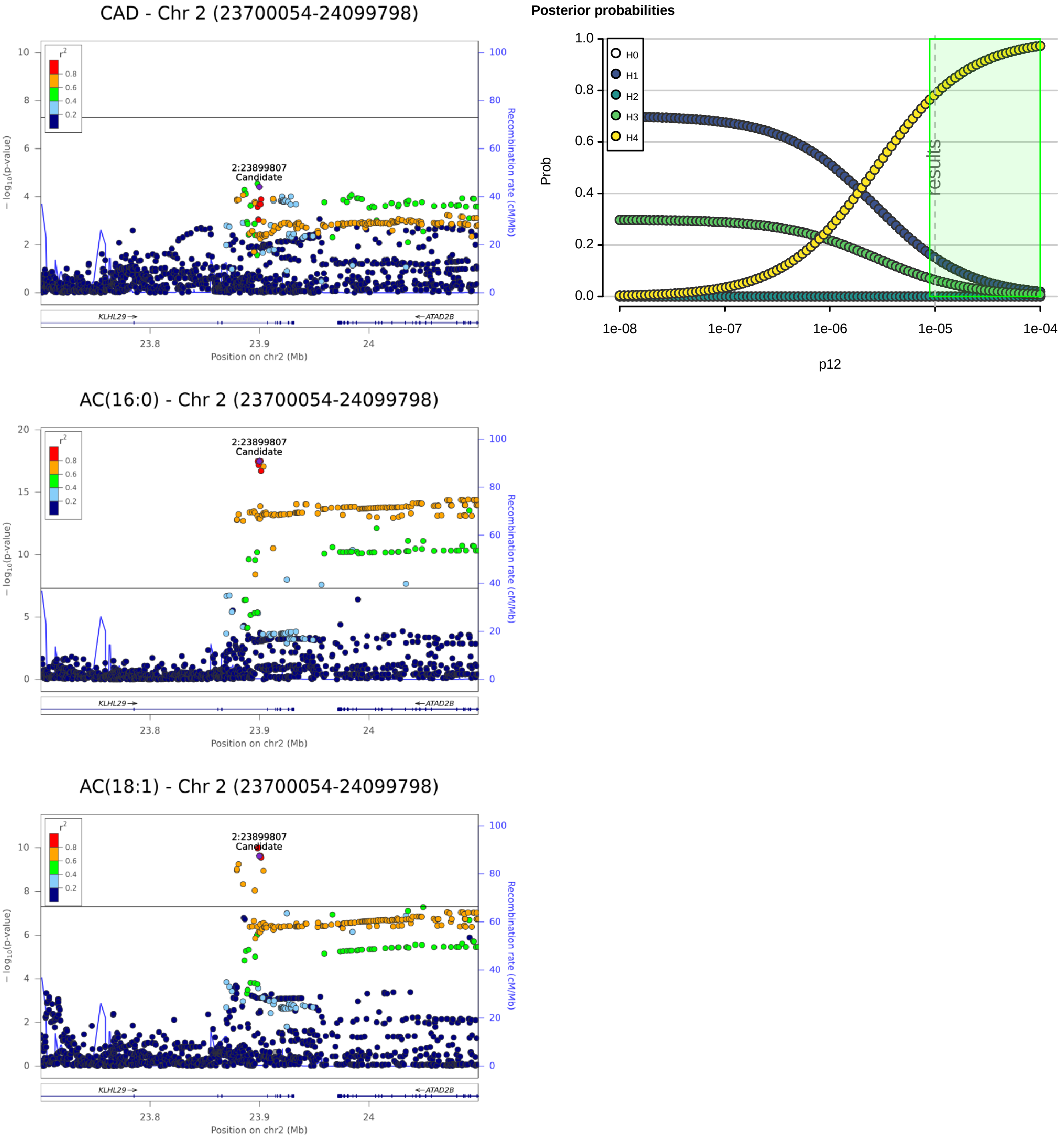

Supplementary Figure 10: Regional association plot of Chr2:23700054-24099798 and coronary artery disease and the top two lipid species that show evidence of colocalization (H3+H4 > 0.8; H4/H3 > 10), along with colocalization sensitivity analysis for the top colocalization, assessing the posterior probability of H0-H4 for different values of prior p_{12}.

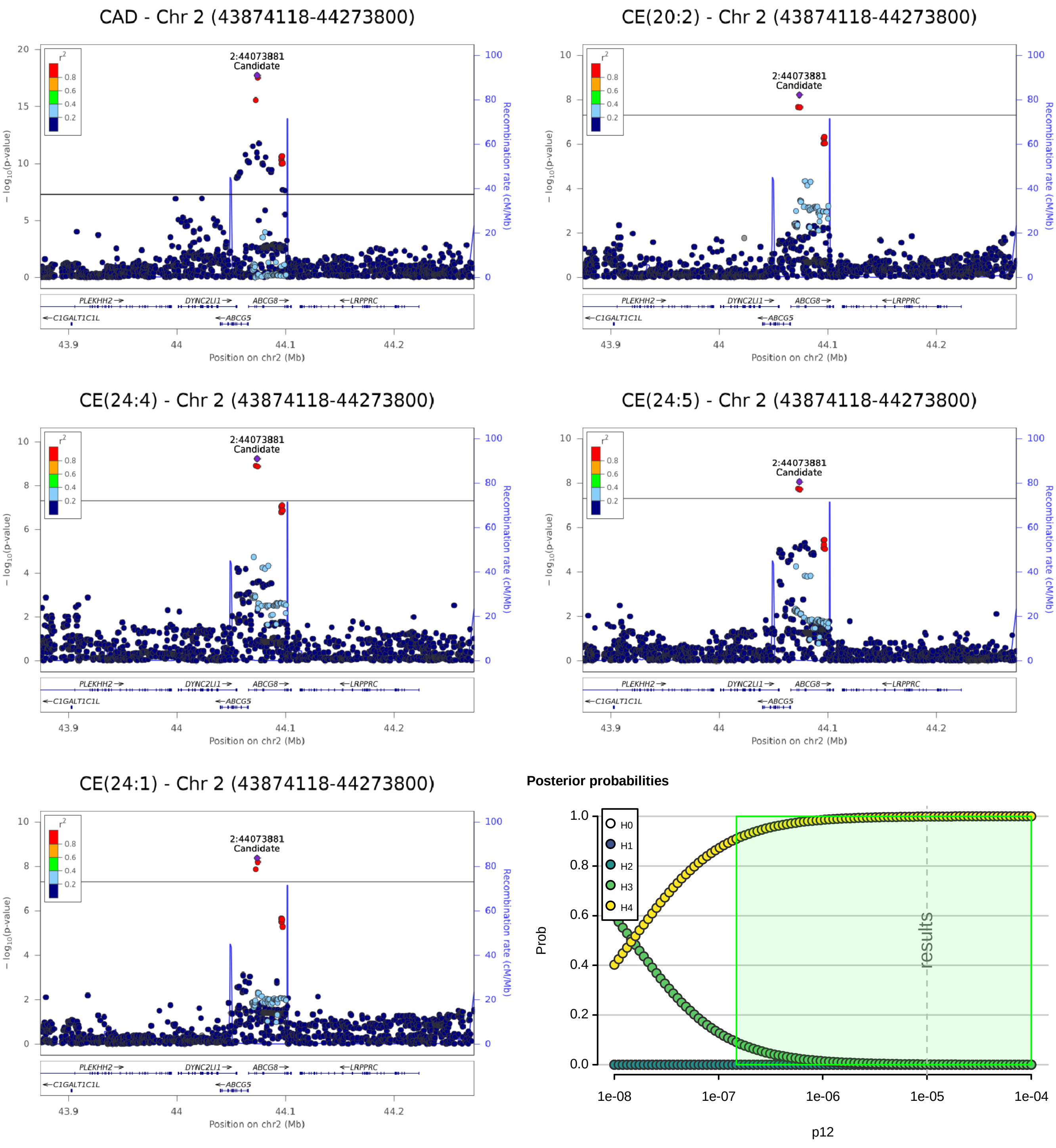

Supplementary Figure 11: Regional association plot of Chr2:43874118-44273800 and coronary artery disease and the top four lipid species that show evidence of colocalization (H3+H4 > 0.8; H4/H3 > 10), along with colocalization sensitivity analysis for the top colocalization, assessing the posterior probability of H0-H4 for different values of prior p_{12}.

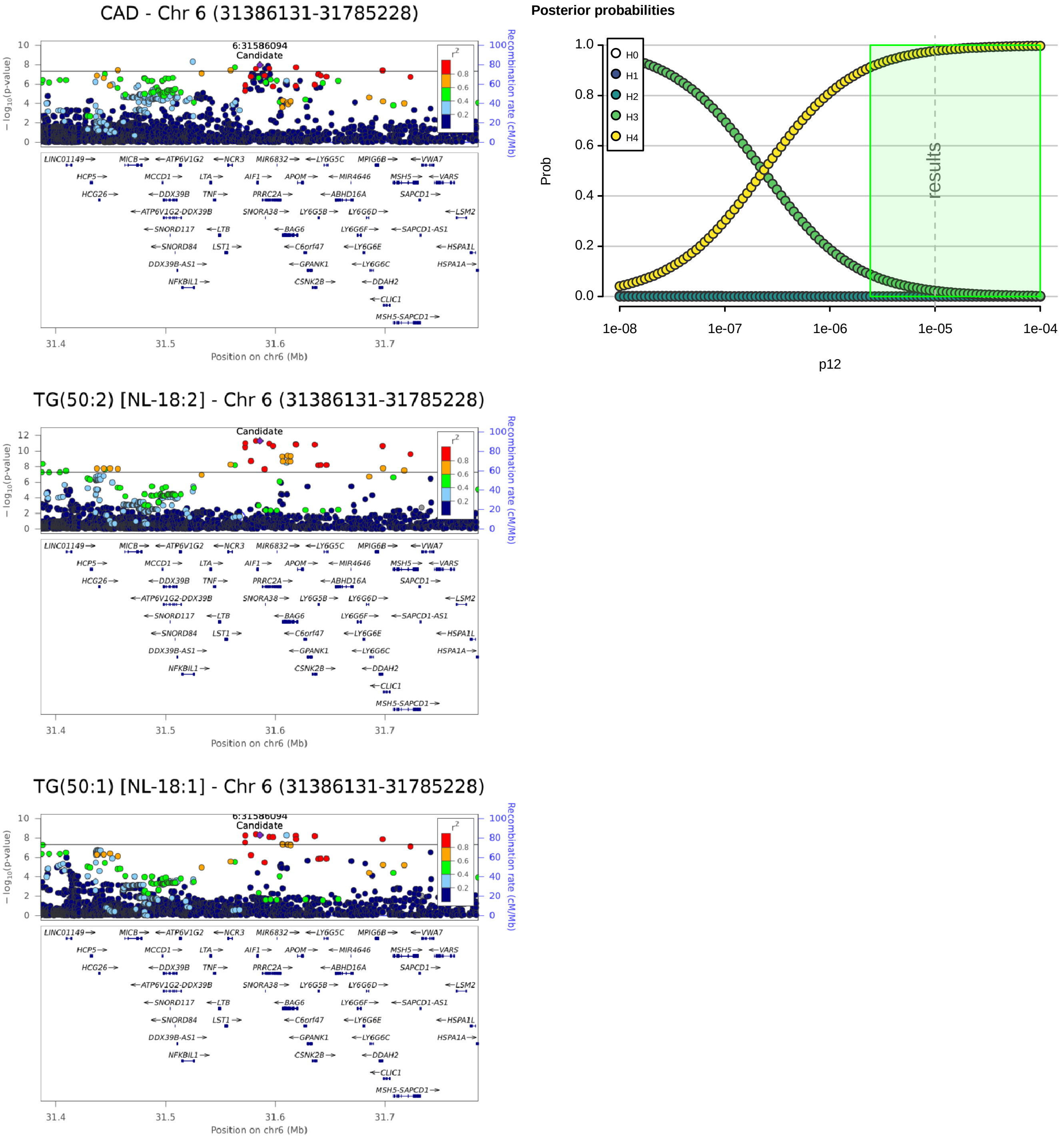

Supplementary Figure 12: Regional association plot of Chr6:31386131-31785228 and coronary artery disease and the top two lipid species that show evidence of colocalization (H3+H4 > 0.8; H4/H3 > 10), along with colocalization sensitivity analysis for the top colocalization, assessing the posterior probability of H0-H4 for different values of prior p_{12}.

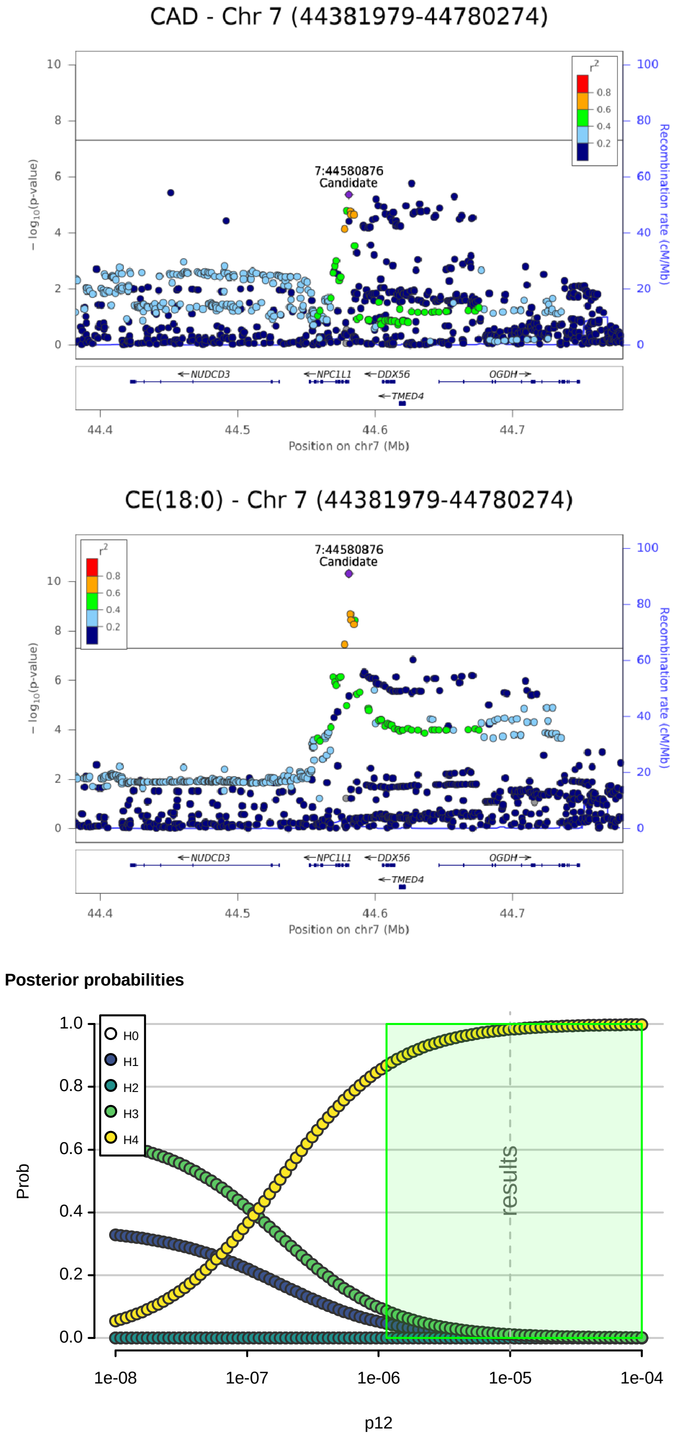

Supplementary Figure 13: Regional association plot of Chr7:44381979-44780274 and coronary artery disease and the top lipid species that shows evidence of colocalization (H3+H4 > 0.8; H4/H3 > 10), along with colocalization sensitivity analysis for the top colocalization, assessing the posterior probability of H0-H4 for different values of prior p_{12}.

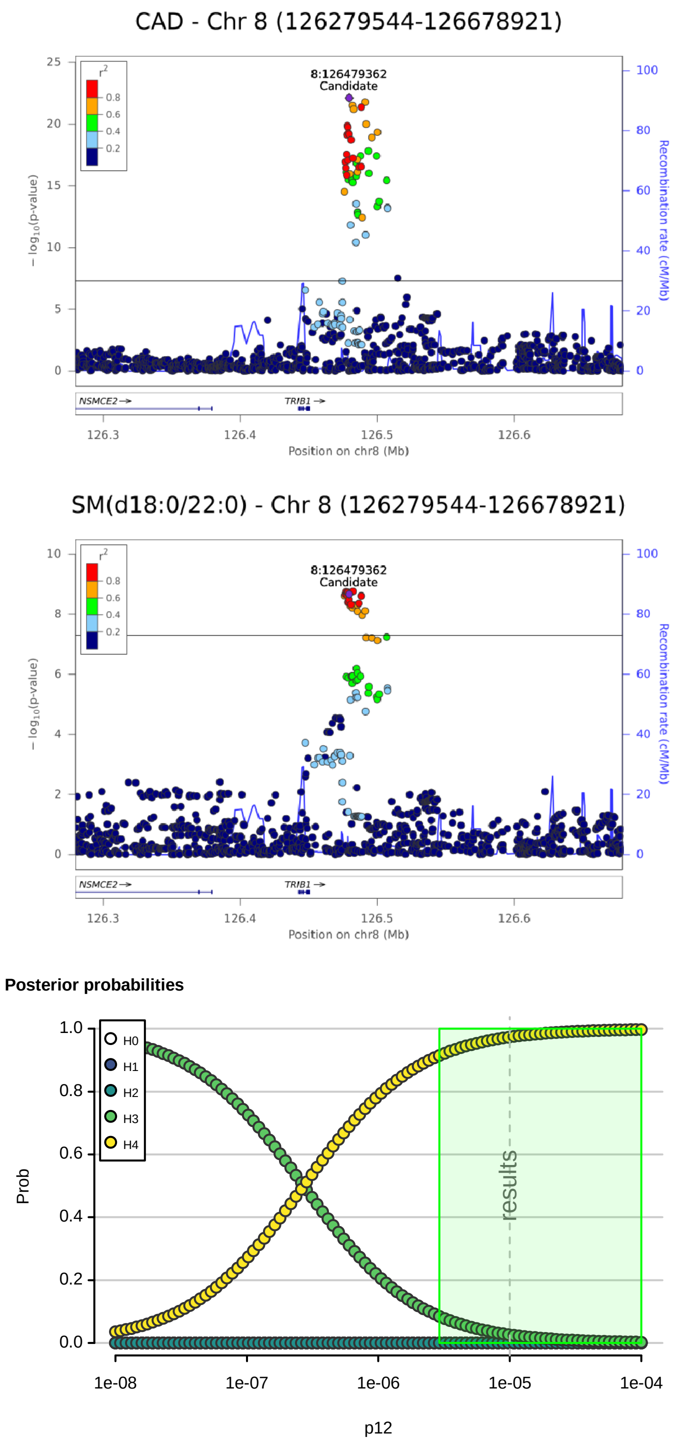

Supplementary Figure 14: Regional association plot of Chr8:126279544-126678921 and coronary artery disease and the top lipid species that shows evidence of colocalization (H3+H4 > 0.8; H4/H3 > 10), along with colocalization sensitivity analysis for the top colocalization, assessing the posterior probability of H0-H4 for different values of prior p_{12}.

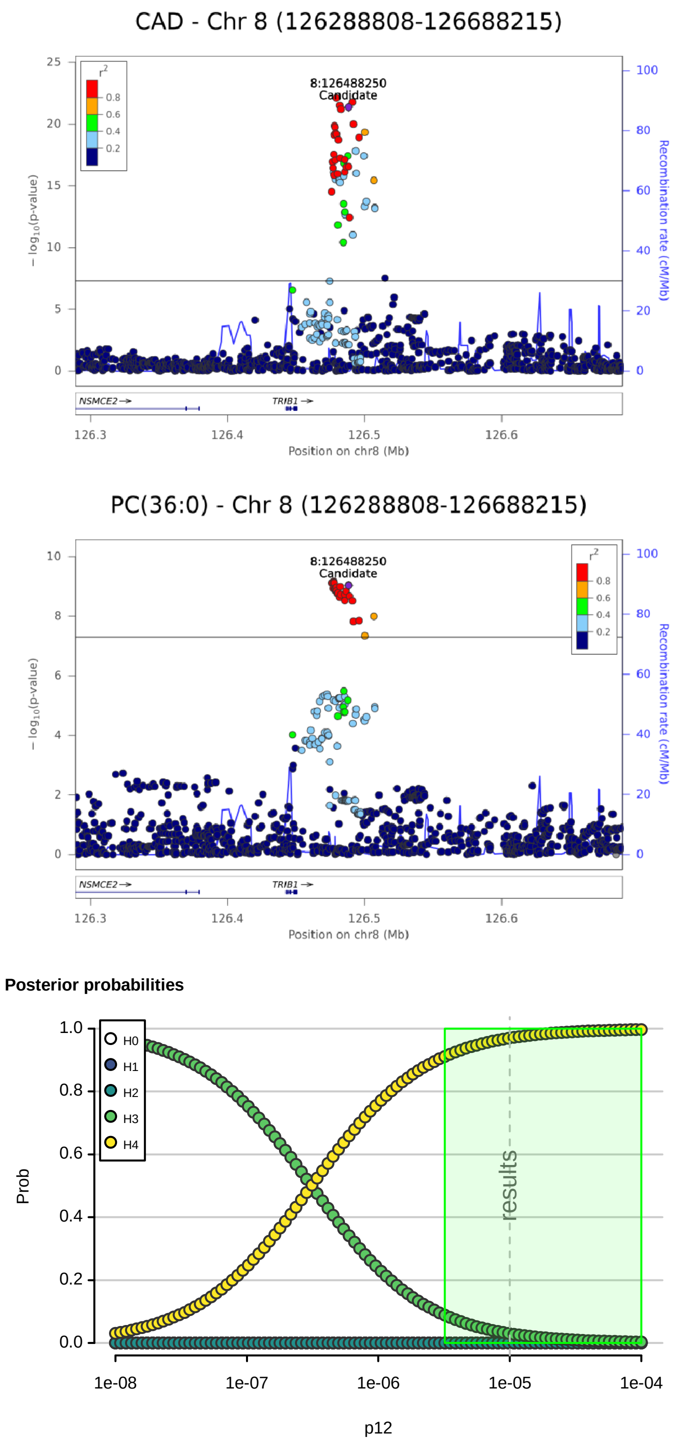

Supplementary Figure 15: Regional association plot of Chr8:126288808-126688215 and coronary artery disease and the top lipid species that shows evidence of colocalization (H3+H4 > 0.8; H4/H3 > 10), along with colocalization sensitivity analysis for the top colocalization, assessing the posterior probability of H0-H4 for different values of prior p_{12}.

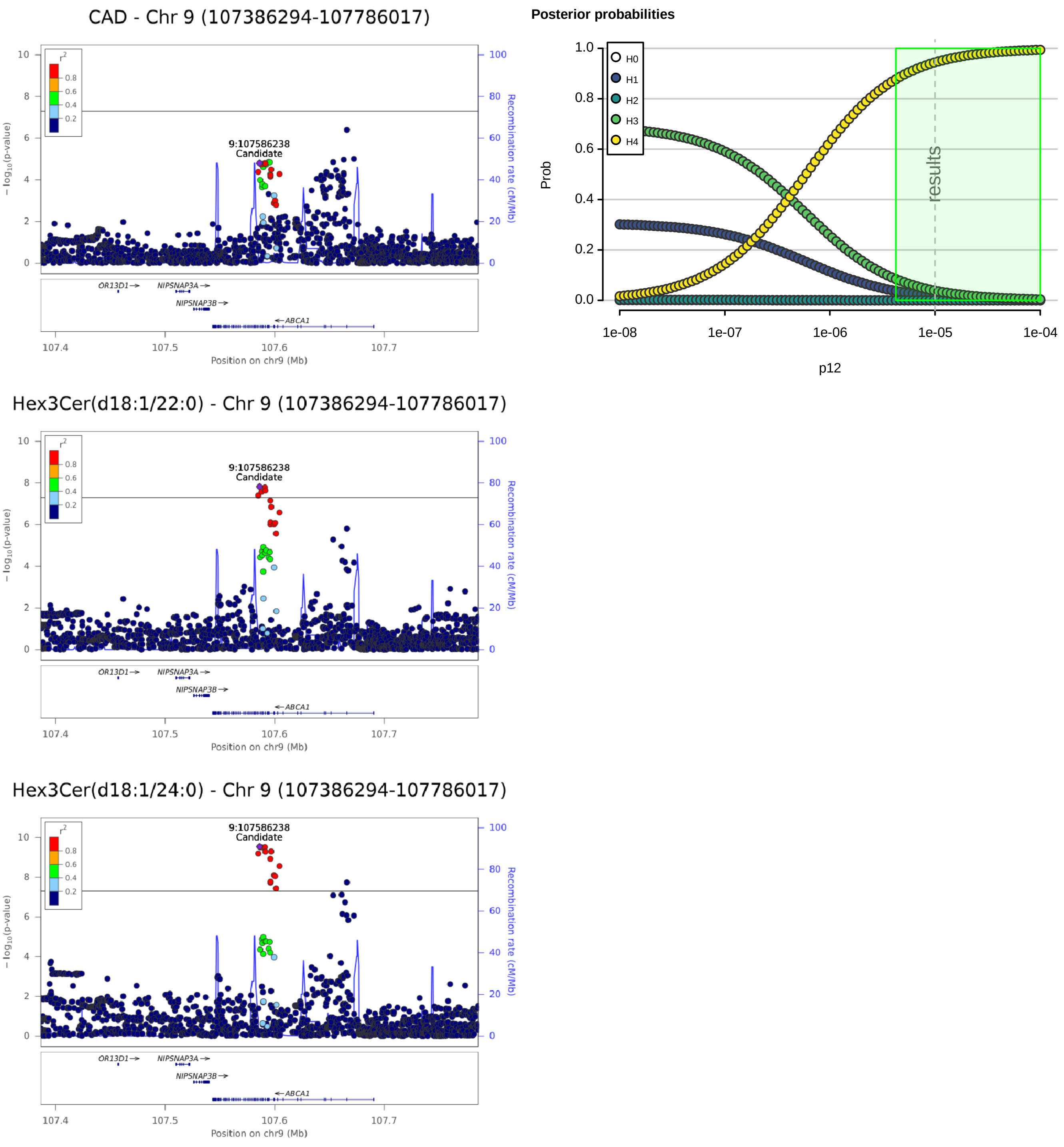

Supplementary Figure 16: Regional association plot of Chr9:107386294-107786017 and coronary artery disease and the top two lipid species that show evidence of colocalization (H3+H4 > 0.8; H4/H3 > 10), along with colocalization sensitivity analysis for the top colocalization, assessing the posterior probability of H0-H4 for different values of prior p_{12}.

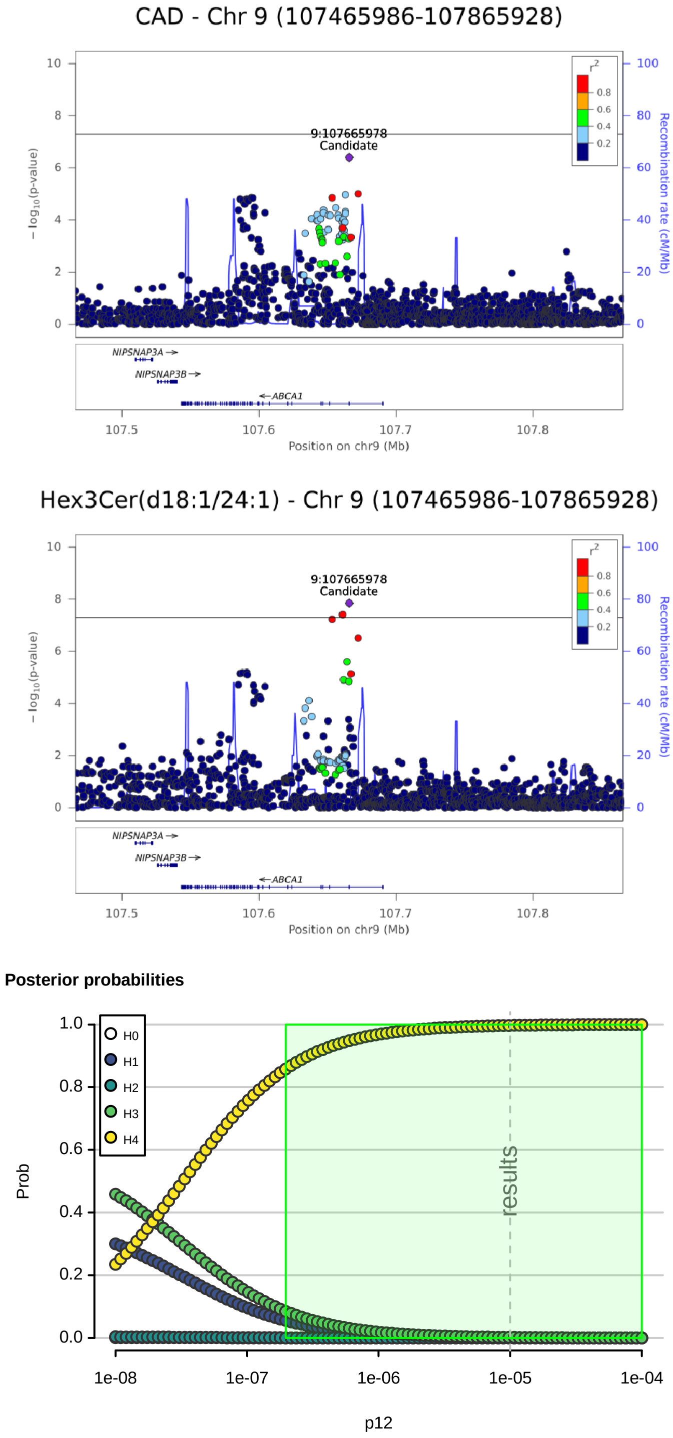

Supplementary Figure 17: Regional association plot of Chr9:107465986-107865928 and coronary artery disease and the top lipid species that shows evidence of colocalization (H3+H4 > 0.8; H4/H3 > 10), along with colocalization sensitivity analysis for the top colocalization, assessing the posterior probability of H0-H4 for different values of prior p_{12}.

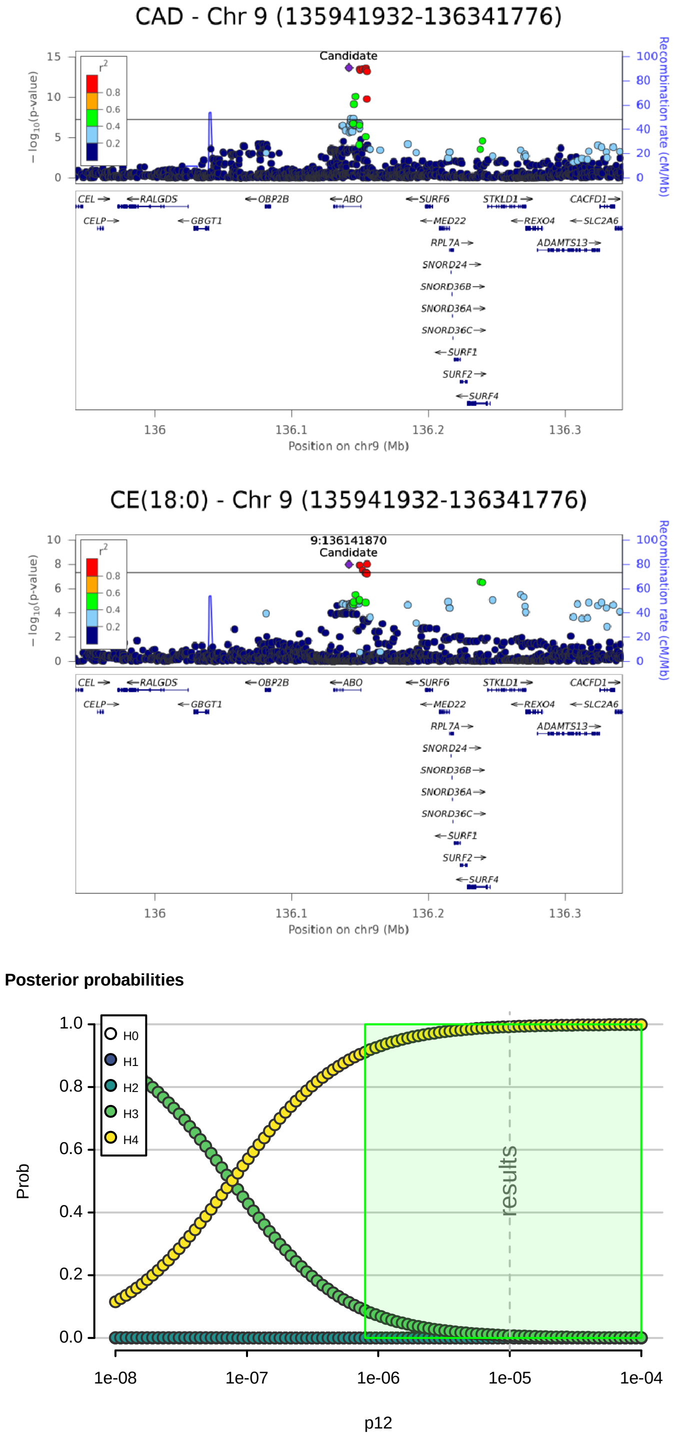

Supplementary Figure 18: Regional association plot of Chr9:135941932-136341776 and coronary artery disease and the top lipid species that shows evidence of colocalization (H3+H4 > 0.8; H4/H3 > 10), along with colocalization sensitivity analysis for the top colocalization, assessing the posterior probability of H0-H4 for different values of prior p_{12}.

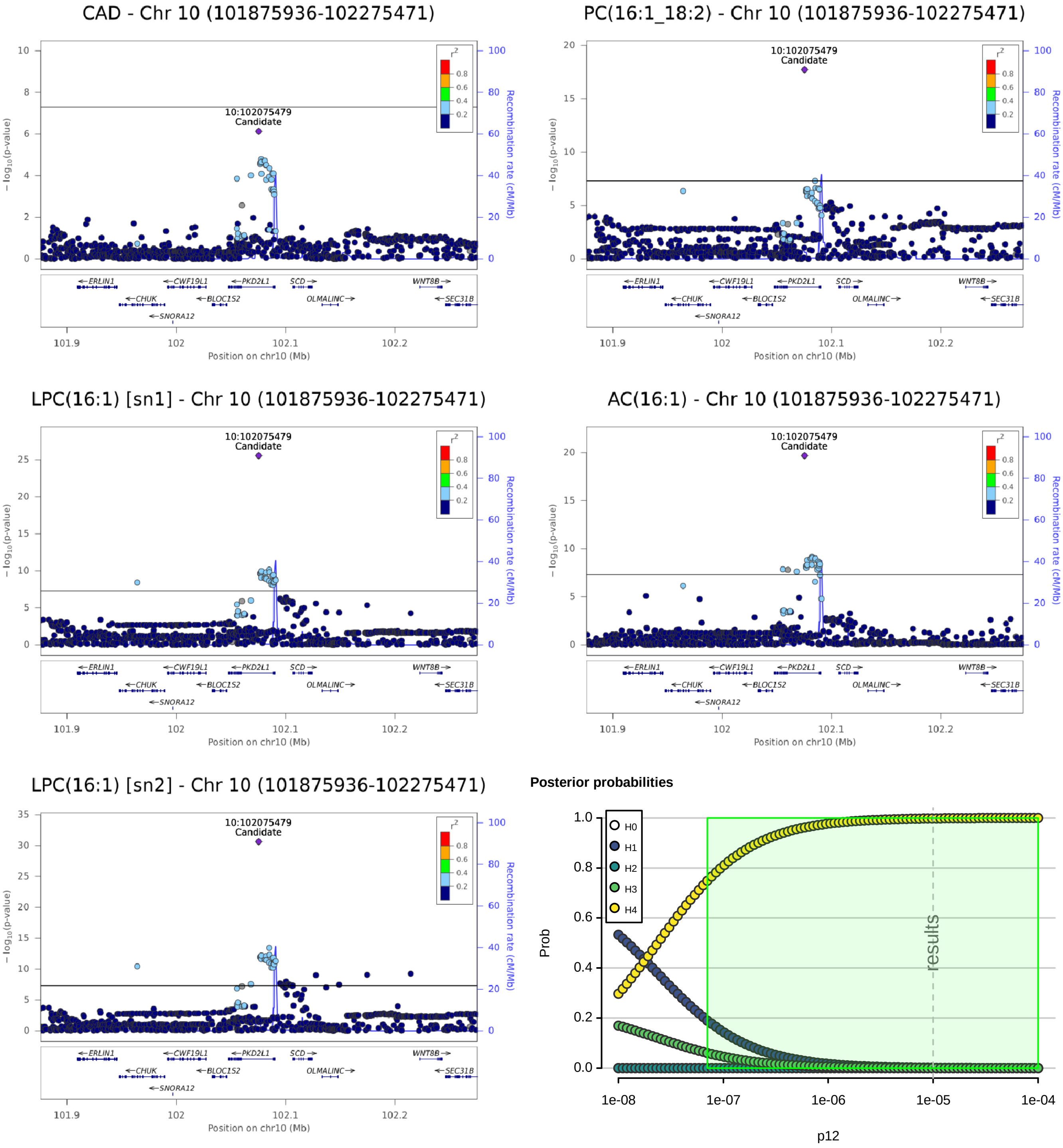

Supplementary Figure 19: Regional association plot of Chr10:101875936-102275471 and coronary artery disease and the top four lipid species that show evidence of colocalization (H3+H4 > 0.8; H4/H3 > 10), along with colocalization sensitivity analysis for the top colocalization, assessing the posterior probability of H0-H4 for different values of prior p_{12}.

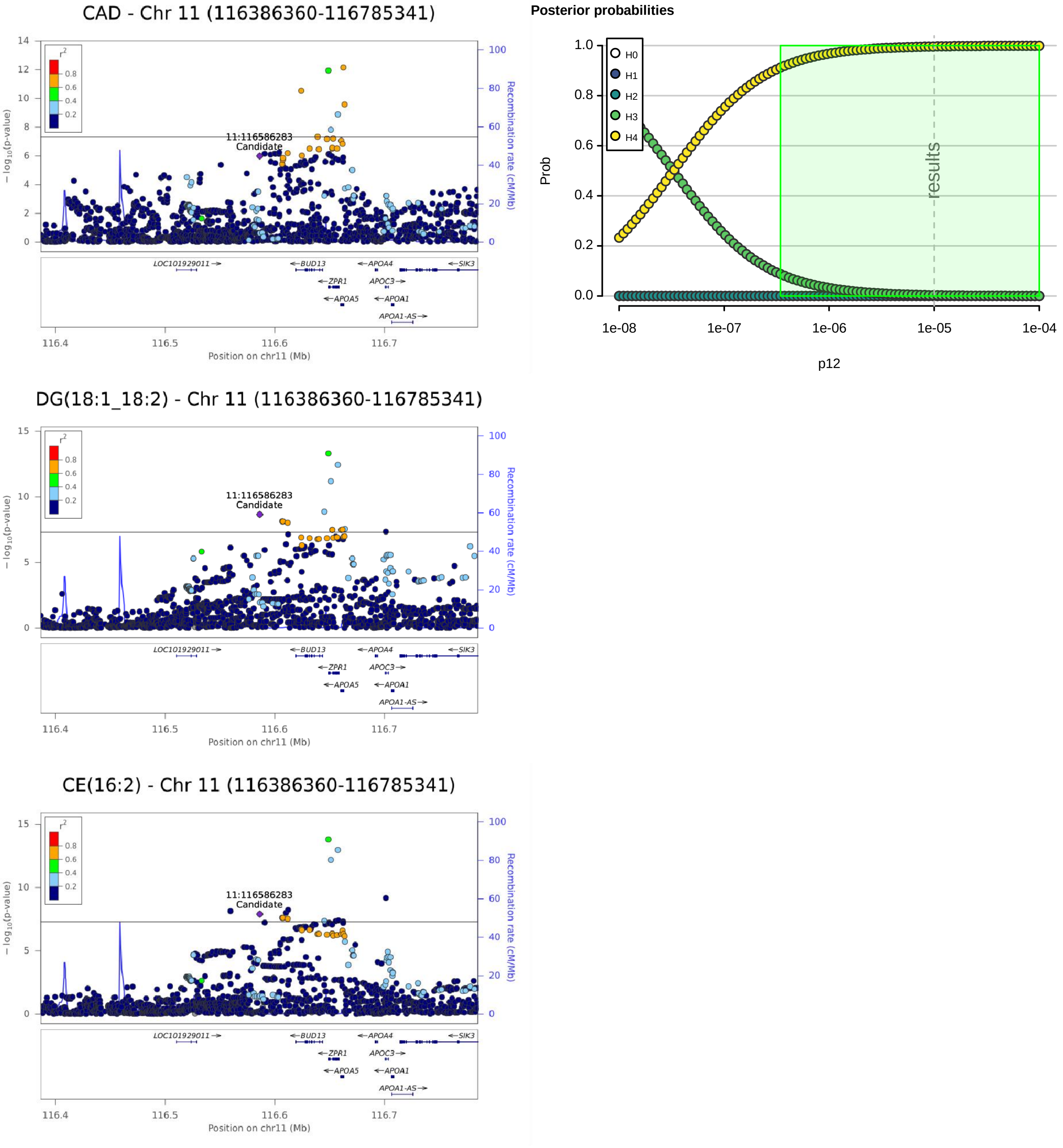

Supplementary Figure 20: Regional association plot of Chr11:116386360-116785341 and coronary artery disease and the top two lipid species that show evidence of colocalization (H3+H4 > 0.8; H4/H3 > 10), along with colocalization sensitivity analysis for the top colocalization, assessing the posterior probability of H0-H4 for different values of prior p_{12}.

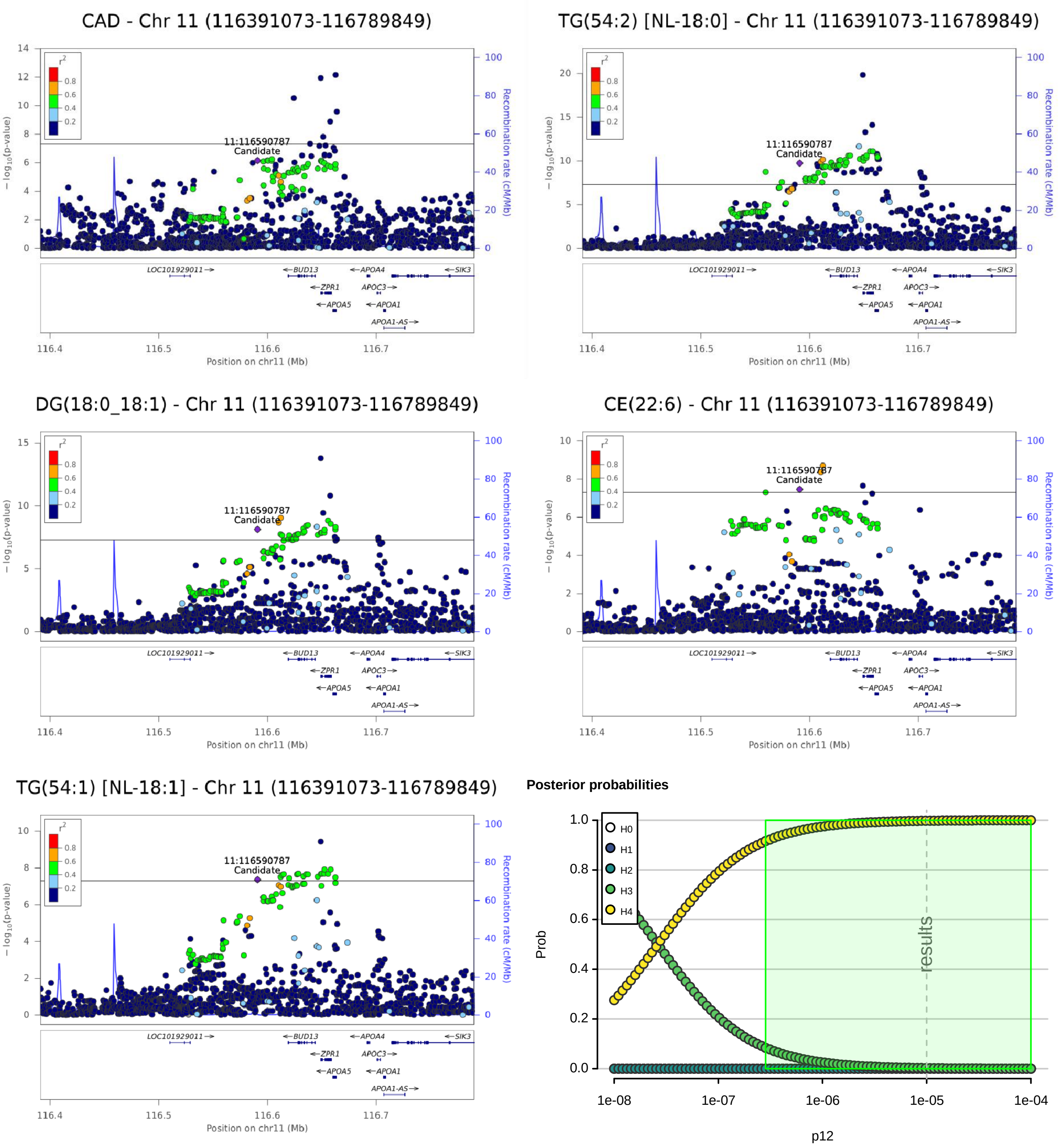

Supplementary Figure 21: Regional association plot of Chr11:116391073-116789849 and coronary artery disease and the top four lipid species that show evidence of colocalization (H3+H4 > 0.8; H4/H3 > 10), along with colocalization sensitivity analysis for the top colocalization, assessing the posterior probability of H0-H4 for different values of prior p_{12}.

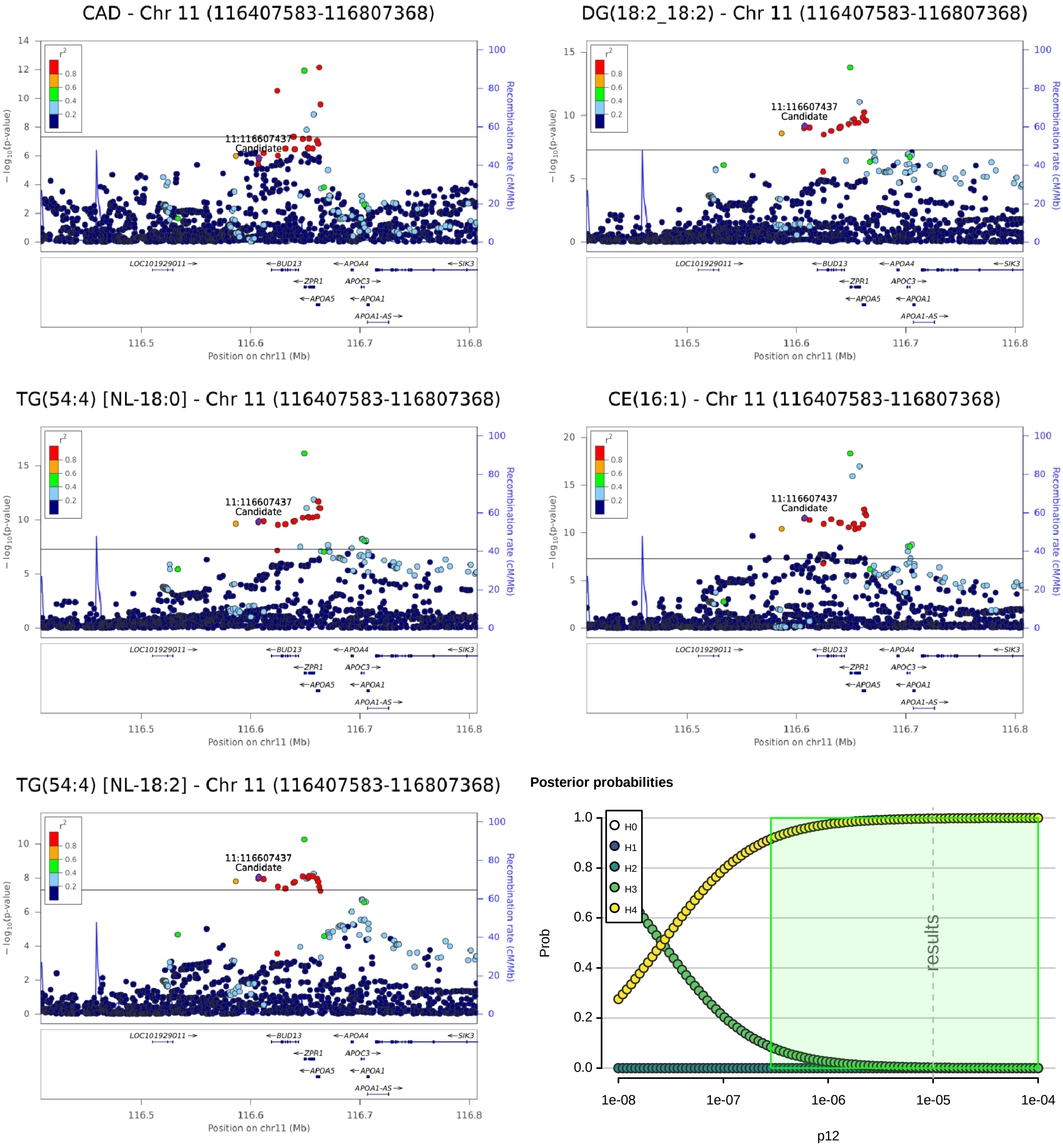

Supplementary Figure 22: Regional association plot of Chr11:116407583-116807368 and coronary artery disease and the top four lipid species that show evidence of colocalization (H3+H4 > 0.8; H4/H3 > 10), along with colocalization sensitivity analysis for the top colocalization, assessing the posterior probability of H0-H4 for different values of prior p_{12}.

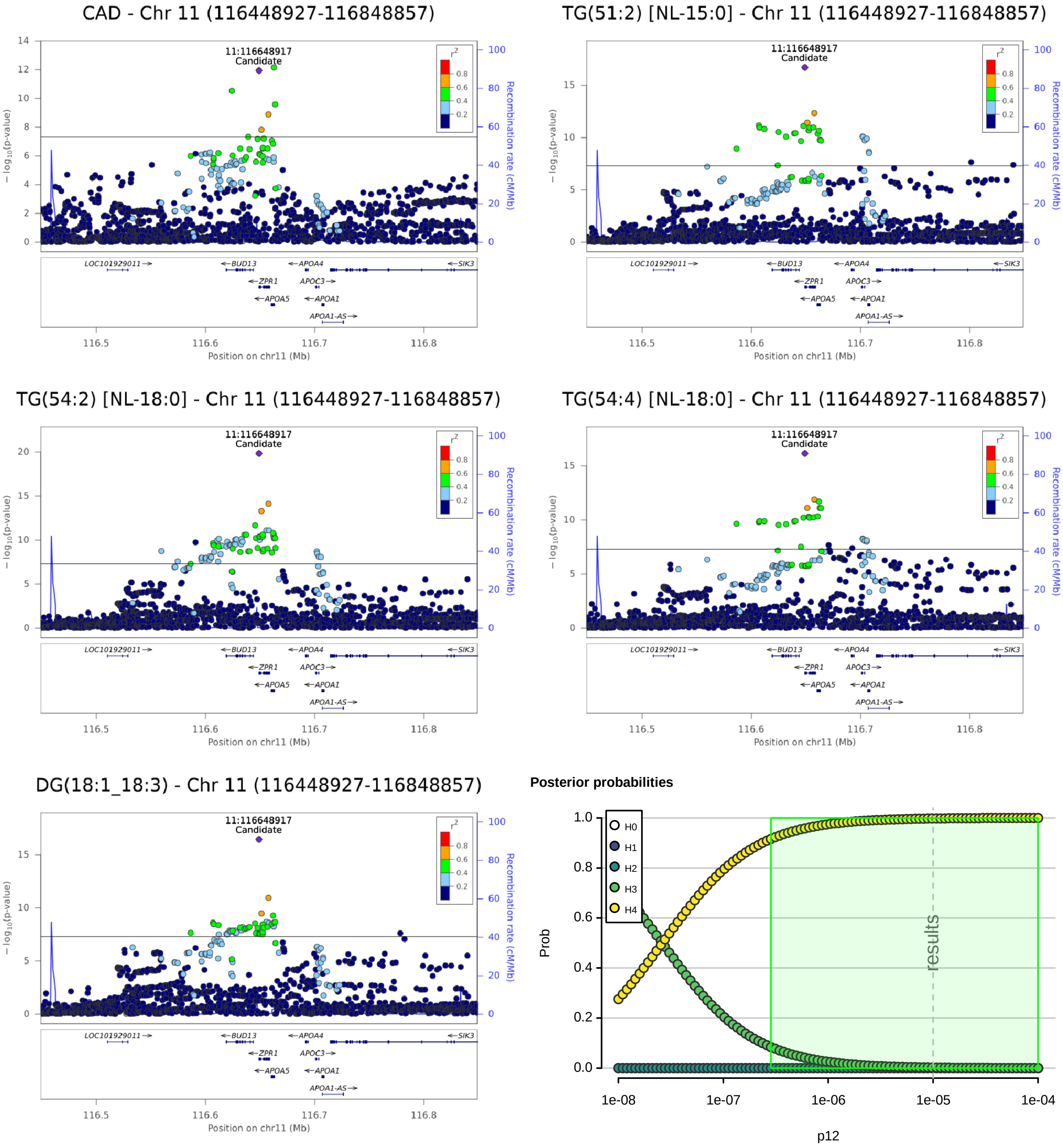

Supplementary Figure 23: Regional association plot of Chr11:116448927-116848857 and coronary artery disease and the top four lipid species that show evidence of colocalization (H3+H4 > 0.8; H4/H3 > 10), along with colocalization sensitivity analysis for the top colocalization, assessing the posterior probability of H0-H4 for different values of prior p_{12}.

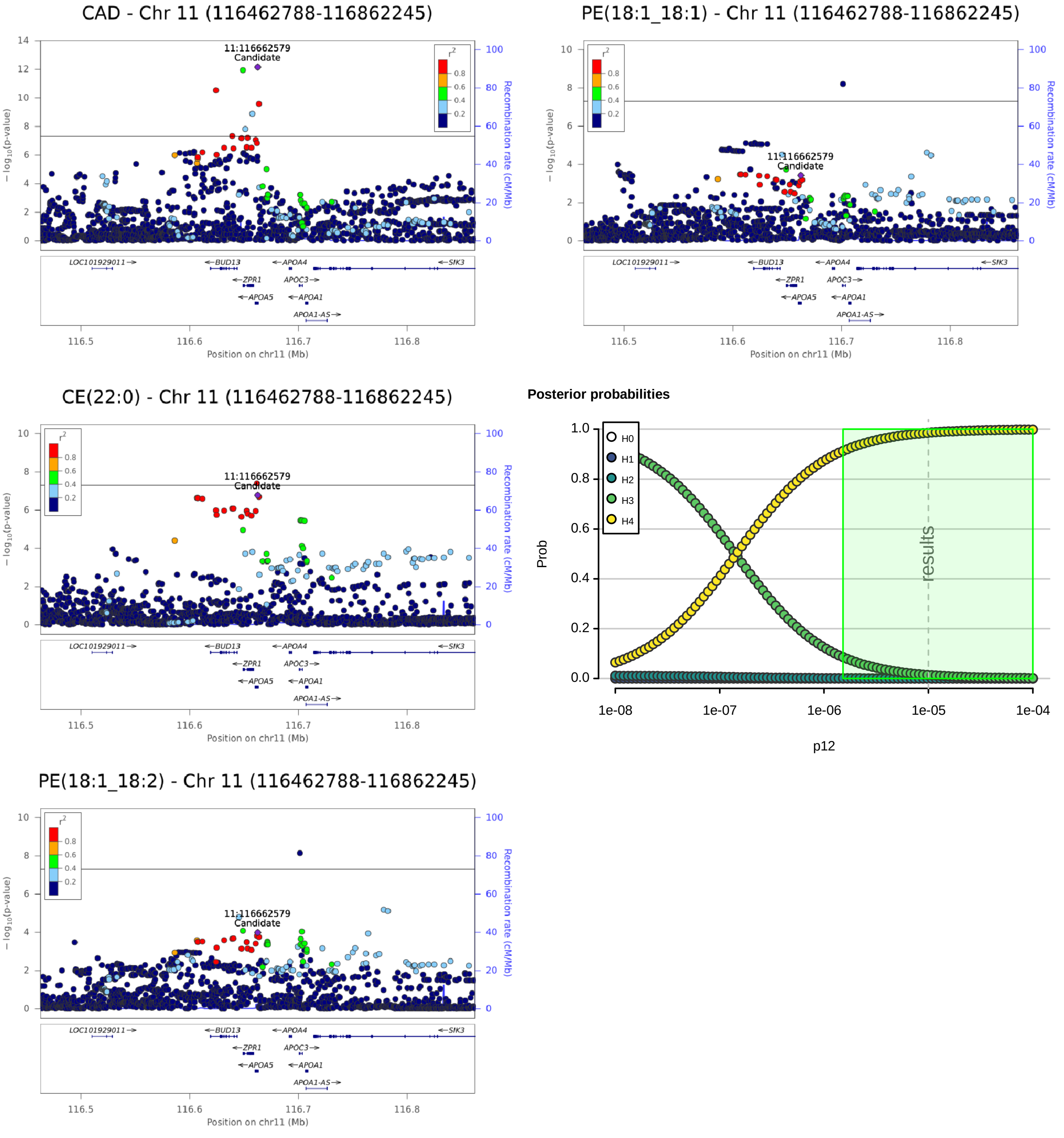

Supplementary Figure 24: Regional association plot of Chr11:116462788-116862245 and coronary artery disease and the top three lipid species that show evidence of colocalization (H3+H4 > 0.8; H4/H3 > 10), along with colocalization sensitivity analysis for the top colocalization, assessing the posterior probability of H0-H4 for different values of prior p_{12}.

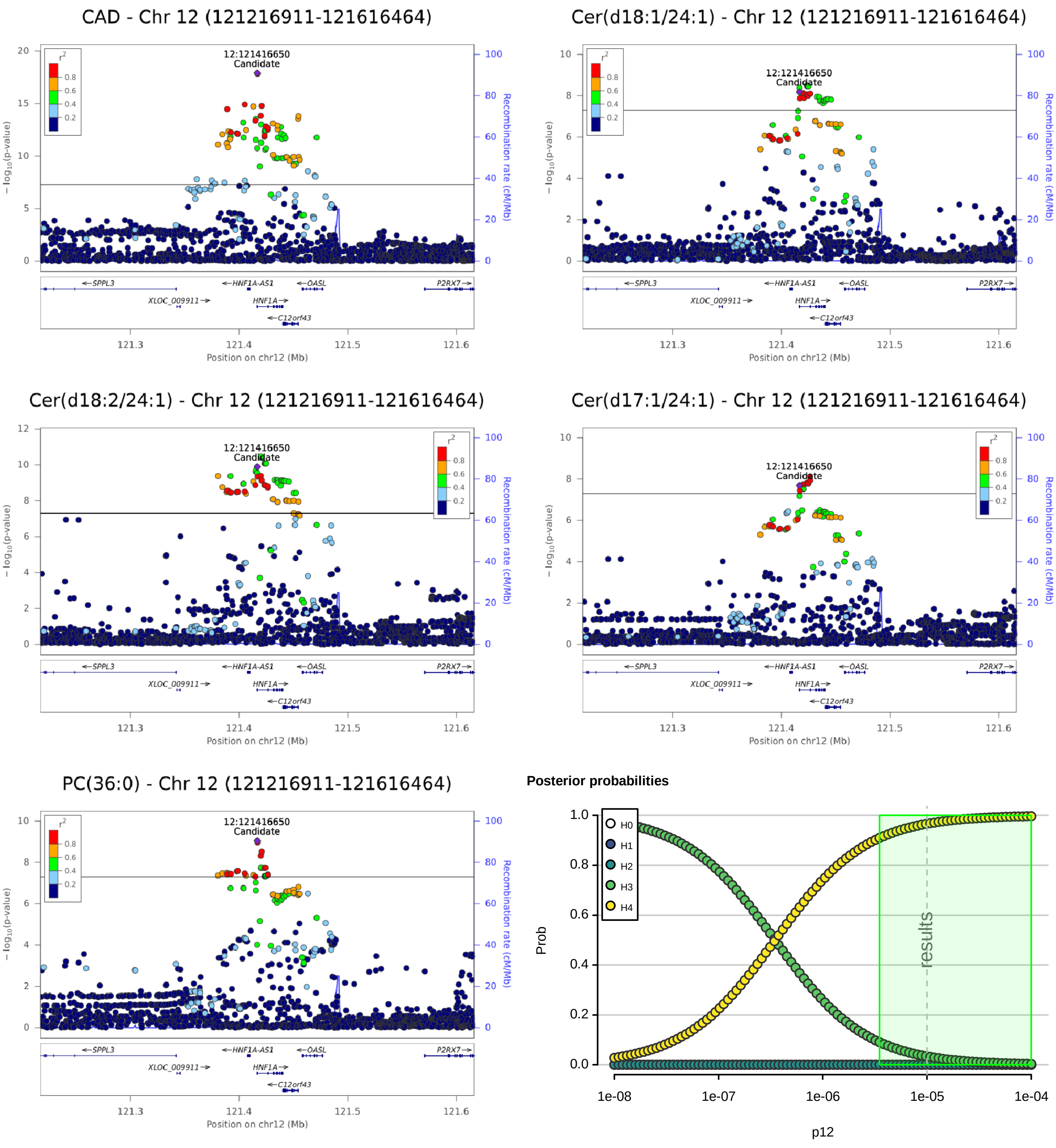

Supplementary Figure 25: Regional association plot of Chr12:121216911-121616464 and coronary artery disease and the top four lipid species that show evidence of colocalization (H3+H4 > 0.8; H4/H3 > 10), along with colocalization sensitivity analysis for the top colocalization, assessing the posterior probability of H0-H4 for different values of prior p_{12}.

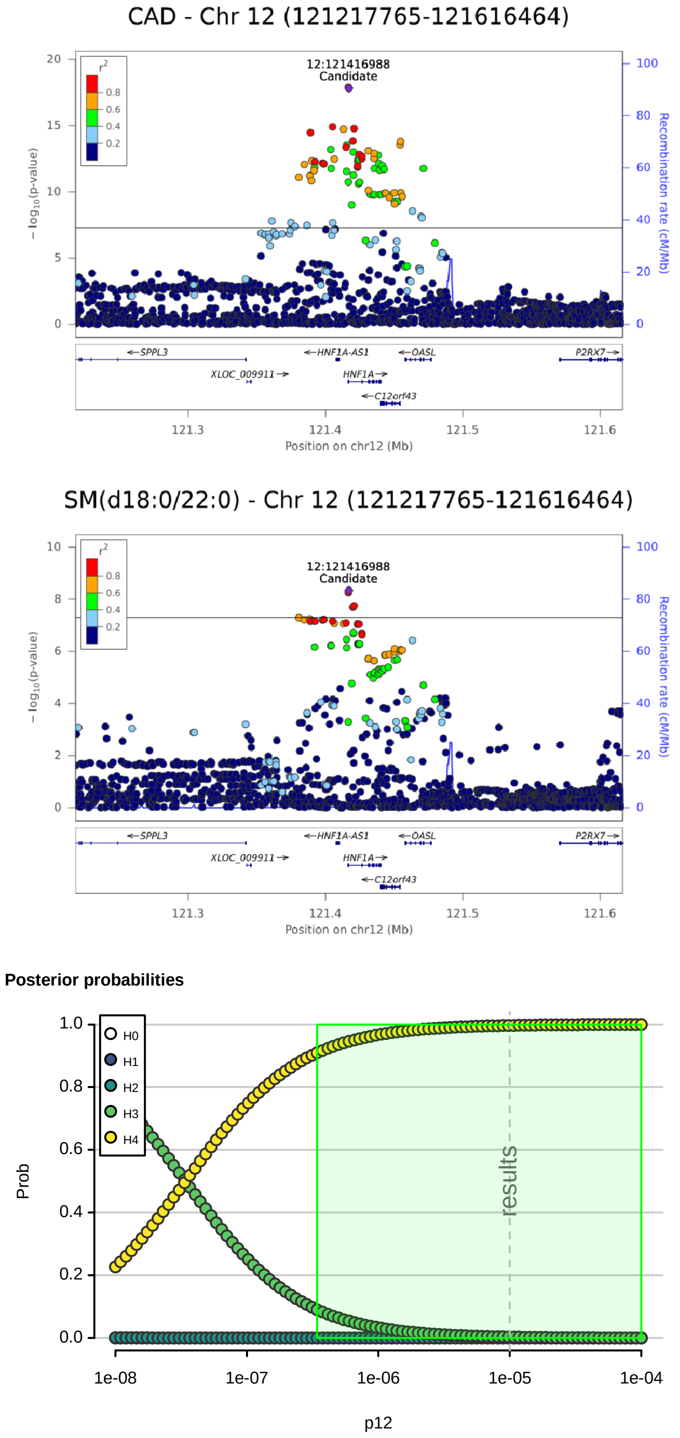

Supplementary Figure 26: Regional association plot of Chr12:121217765-121616464 and coronary artery disease and the top lipid species that shows evidence of colocalization (H3+H4 > 0.8; H4/H3 > 10), along with colocalization sensitivity analysis for the top colocalization, assessing the posterior probability of H0-H4 for different values of prior p_{12}.

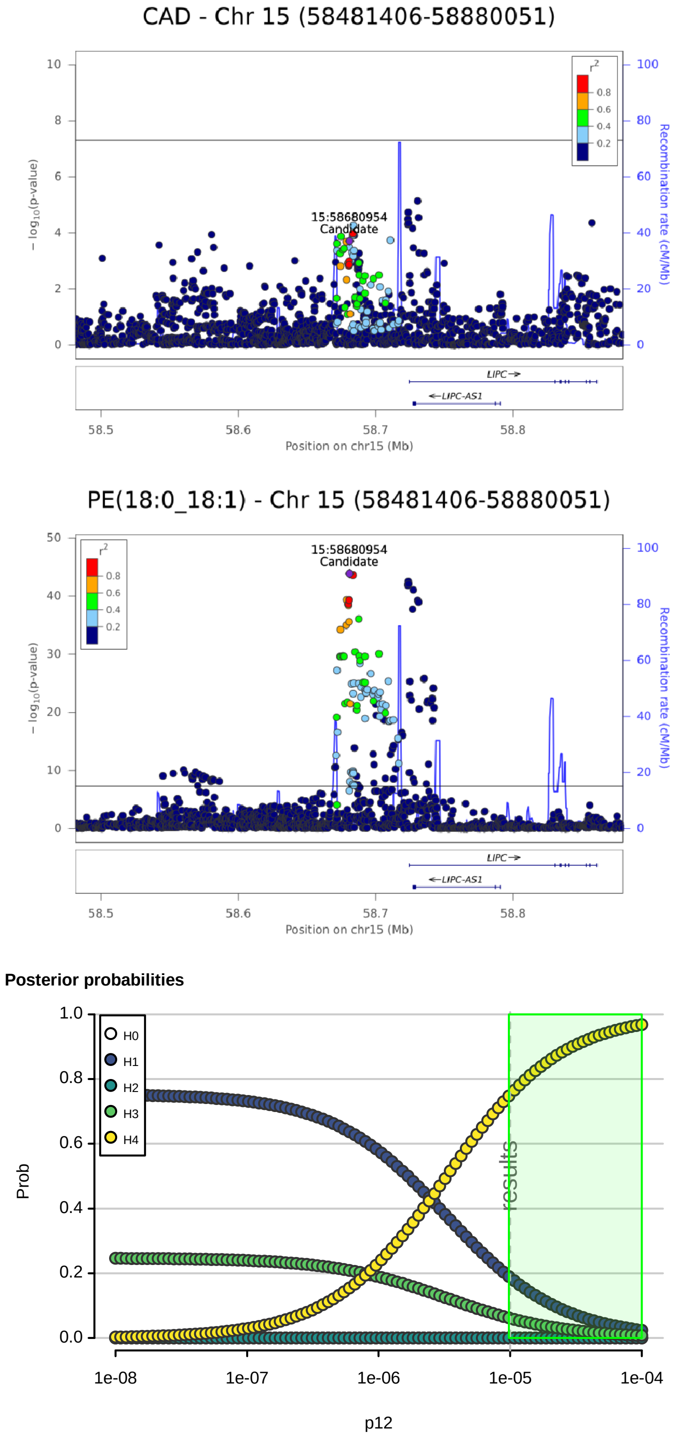

Supplementary Figure 27: Regional association plot of Chr15:58481406-58880051 and coronary artery disease and the top lipid species that shows evidence of colocalization (H3+H4 > 0.8; H4/H3 > 10), along with colocalization sensitivity analysis for the top colocalization, assessing the posterior probability of H0-H4 for different values of prior p_{12}.

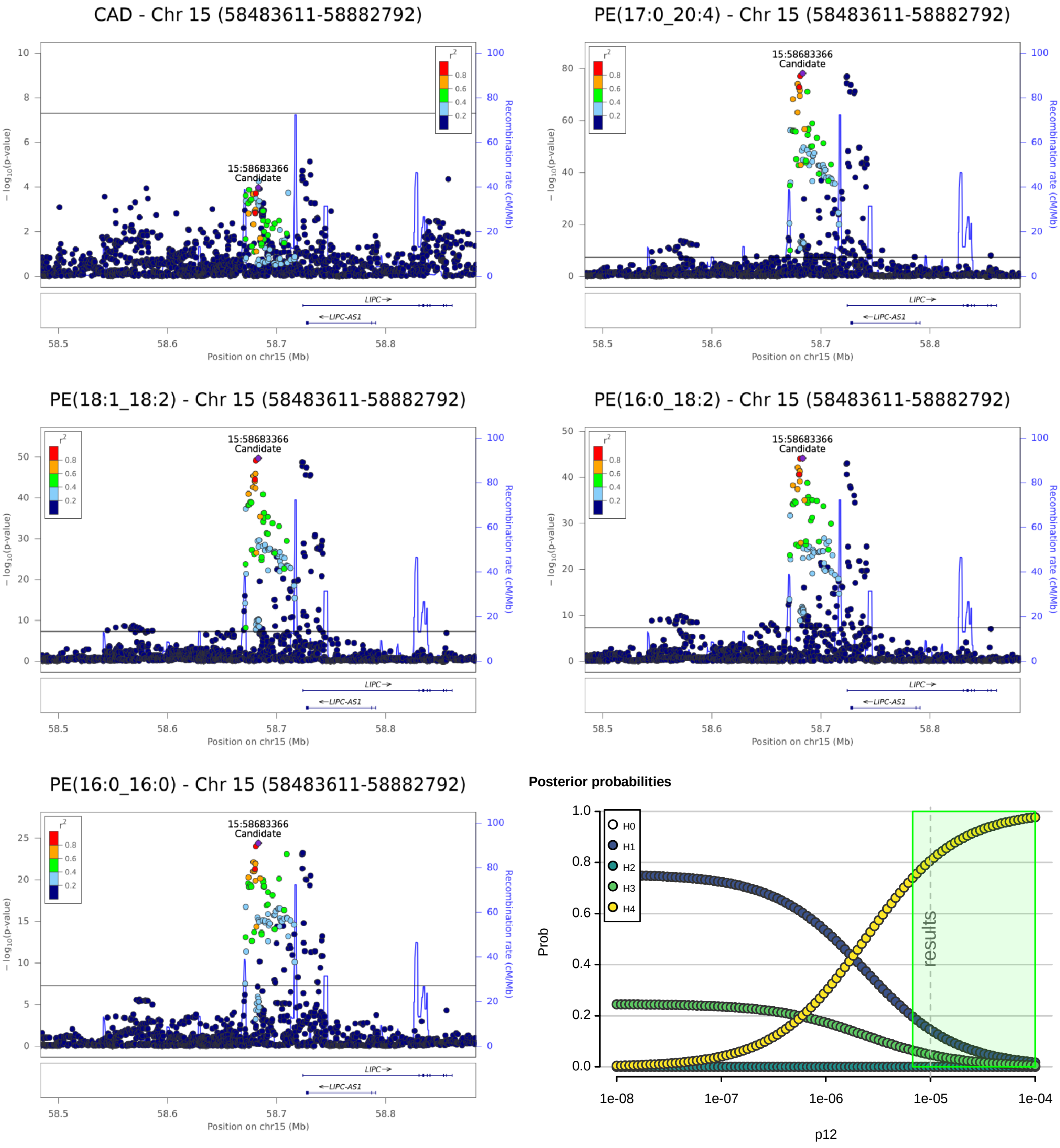

Supplementary Figure 28: Regional association plot of Chr15:58483611-58882792 and coronary artery disease and the top four lipid species that show evidence of colocalization (H3+H4 > 0.8; H4/H3 > 10), along with colocalization sensitivity analysis for the top colocalization, assessing the posterior probability of H0-H4 for different values of prior p_{12}.

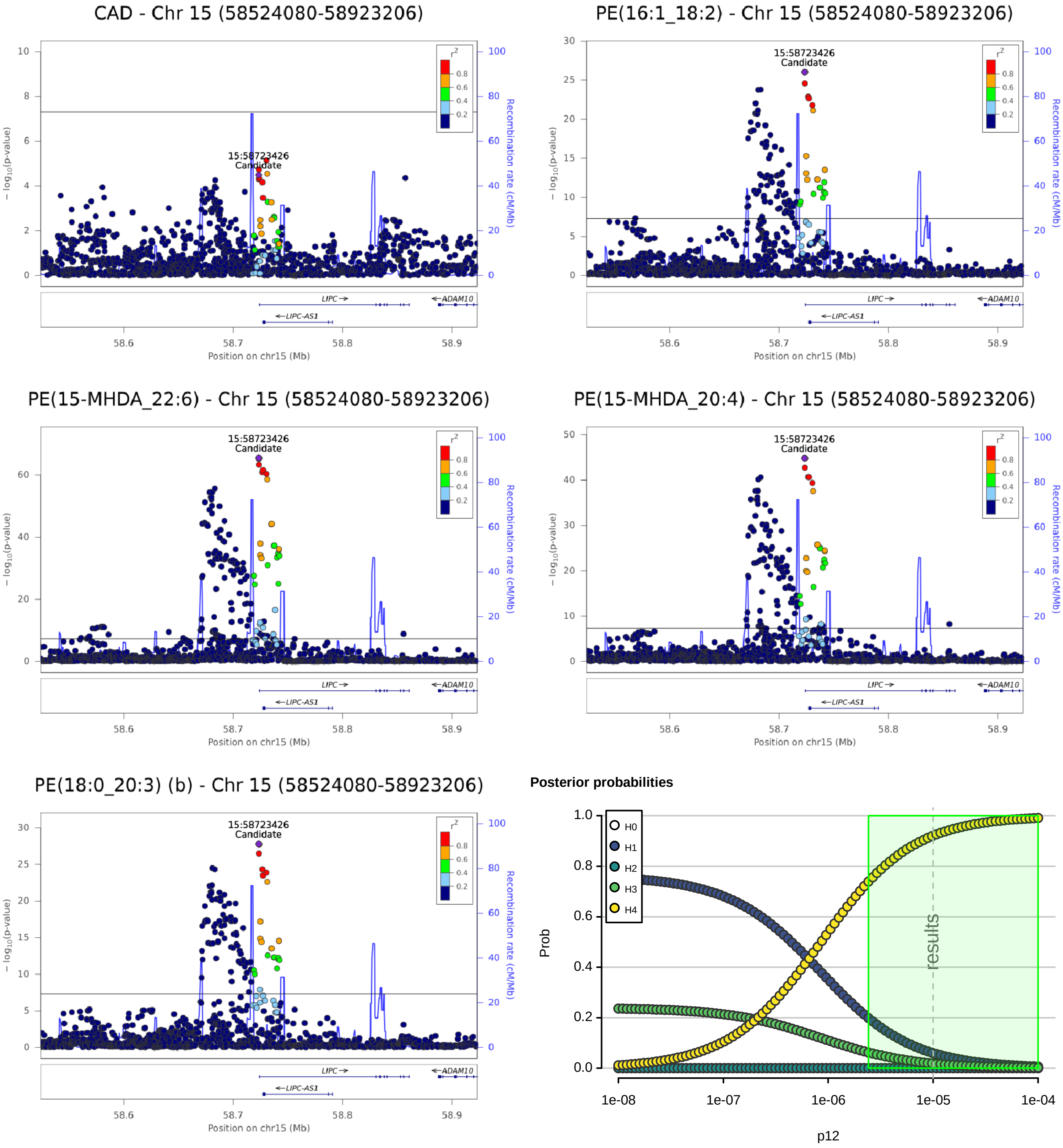

Supplementary Figure 29: Regional association plot of Chr15:58524080-58923206 and coronary artery disease and the top four lipid species that show evidence of colocalization (H3+H4 > 0.8; H4/H3 > 10), along with colocalization sensitivity analysis for the top colocalization, assessing the posterior probability of H0-H4 for different values of prior p_{12}.

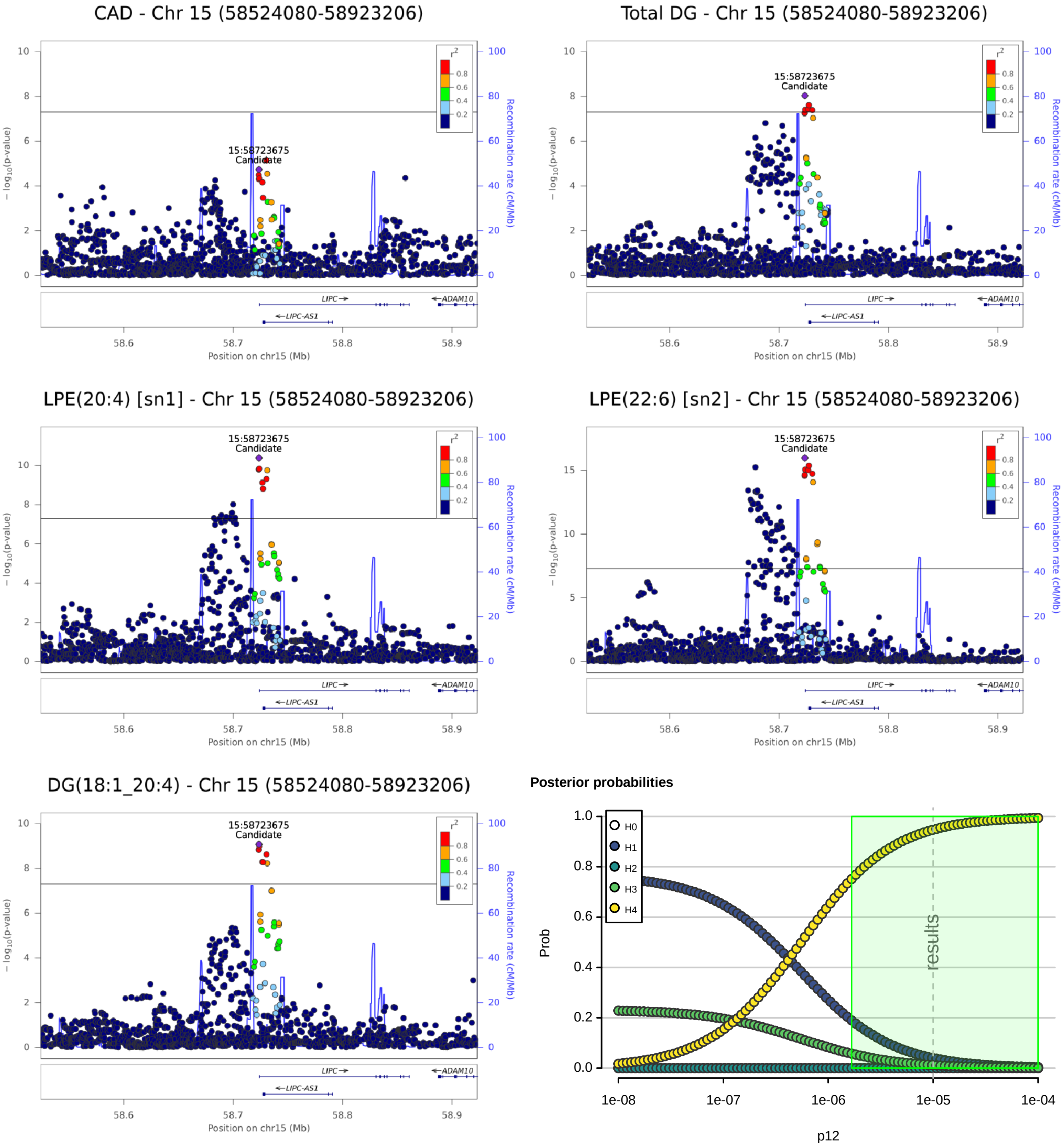

Supplementary Figure 30: Regional association plot of Chr15:58524080-58923206 and coronary artery disease and the top four lipid species that show evidence of colocalization (H3+H4 > 0.8; H4/H3 > 10), along with colocalization sensitivity analysis for the top colocalization, assessing the posterior probability of H0-H4 for different values of prior p_{12}.

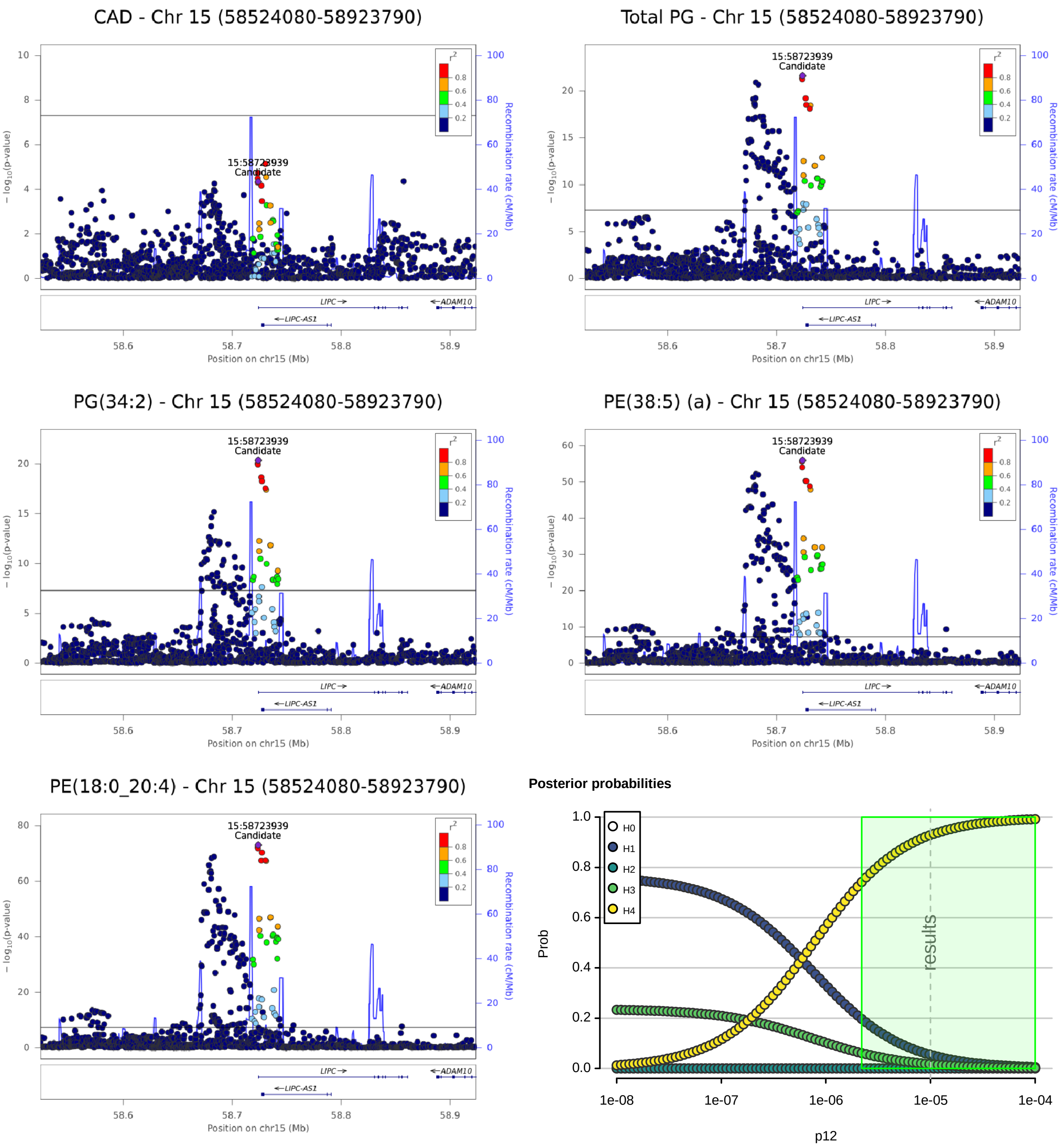

Supplementary Figure 31: Regional association plot of Chr15:58524080-58923790 and coronary artery disease and the top four lipid species that show evidence of colocalization (H3+H4 > 0.8; H4/H3 > 10), along with colocalization sensitivity analysis for the top colocalization, assessing the posterior probability of H0-H4 for different values of prior p_{12}.

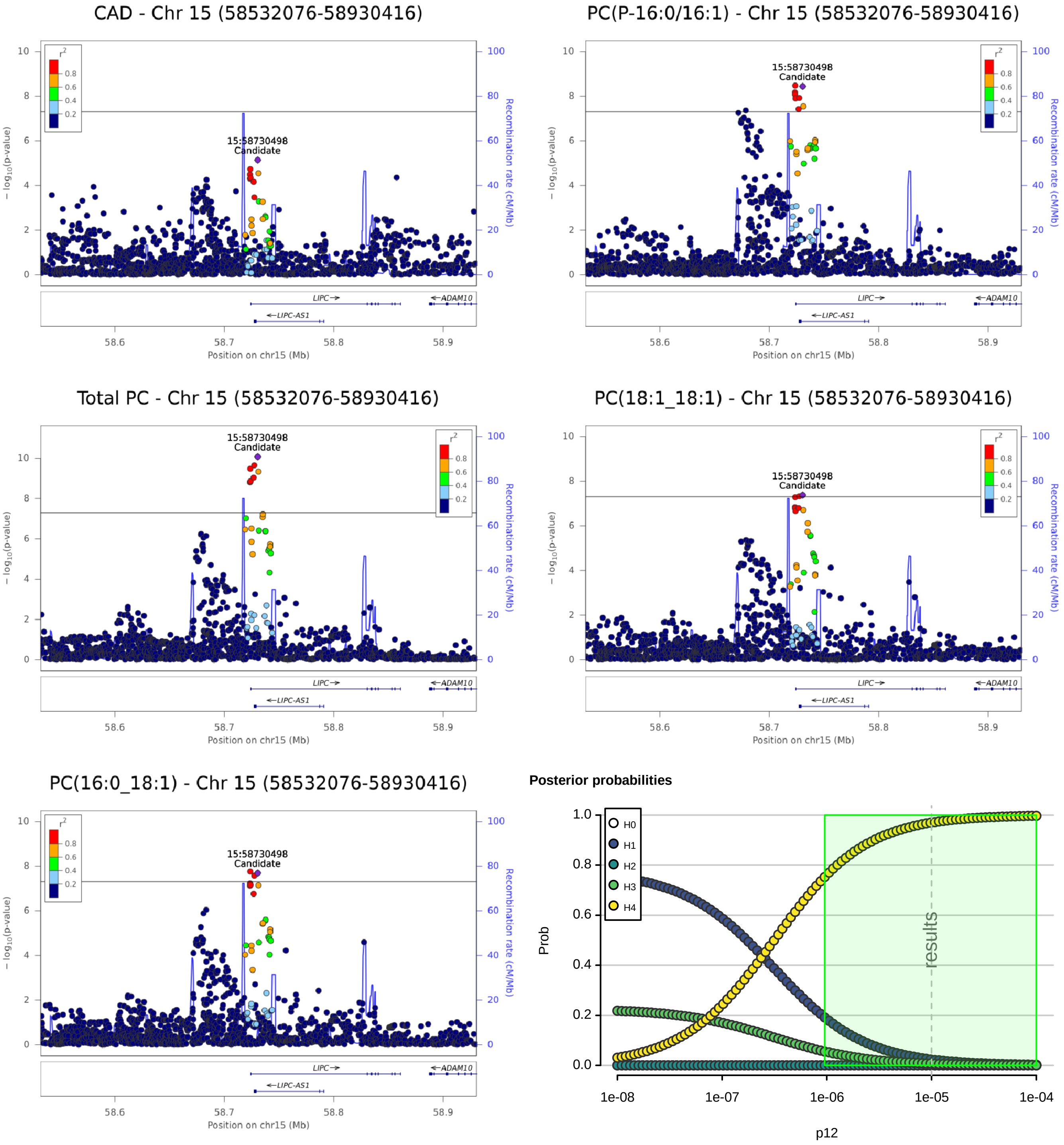

Supplementary Figure 32: Regional association plot of Chr15:58532076-58930416 and coronary artery disease and the top four lipid species that show evidence of colocalization (H3+H4 > 0.8; H4/H3 > 10), along with colocalization sensitivity analysis for the top colocalization, assessing the posterior probability of H0-H4 for different values of prior p_{12}.

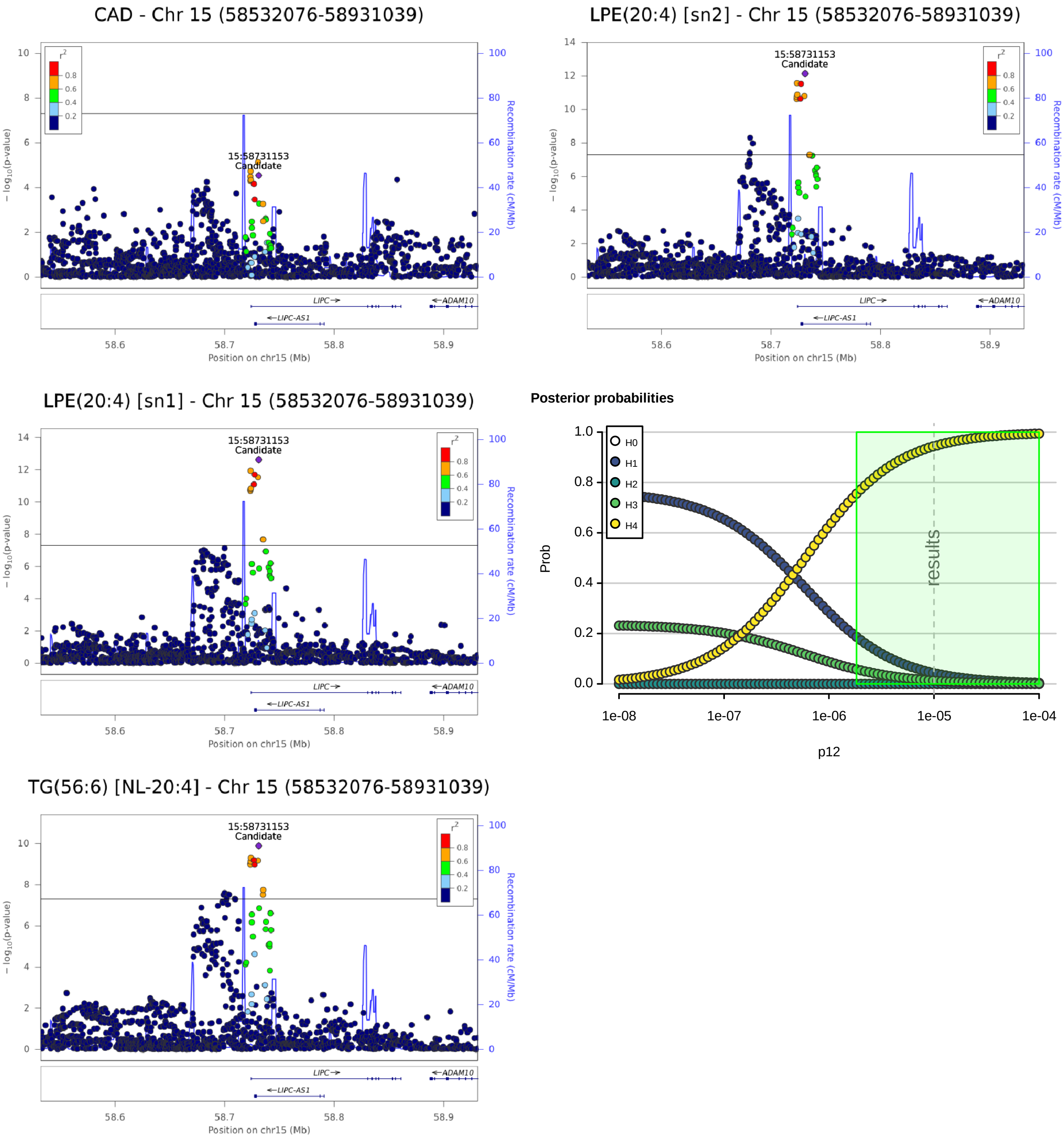

Supplementary Figure 33: Regional association plot of Chr15:58532076-58931039 and coronary artery disease and the top three lipid species that show evidence of colocalization (H3+H4 > 0.8; H4/H3 > 10), along with colocalization sensitivity analysis for the top colocalization, assessing the posterior probability of H0-H4 for different values of prior p_{12}.

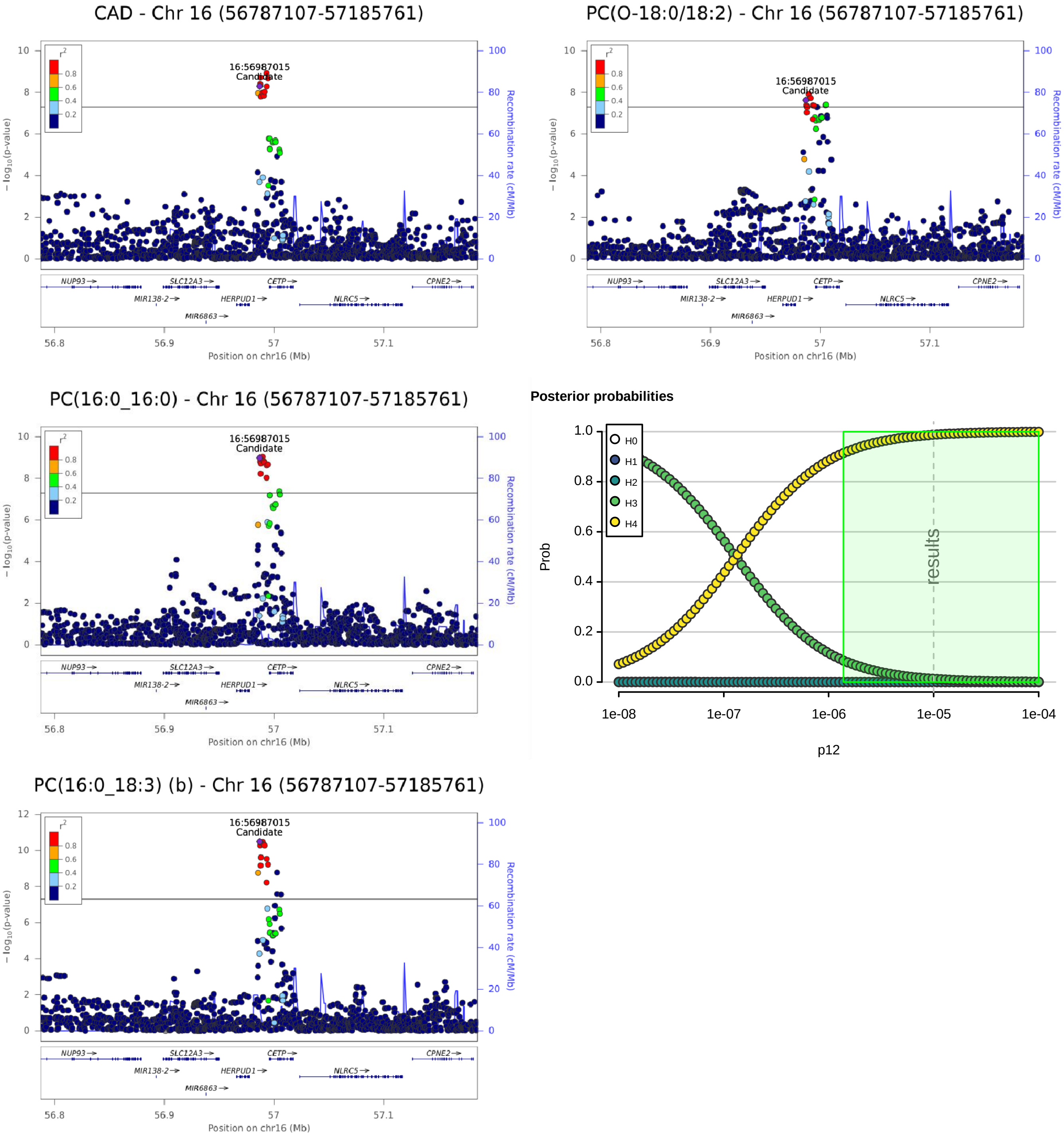

Supplementary Figure 34: Regional association plot of Chr16:56787107-57185761 and coronary artery disease and the top three lipid species that show evidence of colocalization (H3+H4 > 0.8; H4/H3 > 10), along with colocalization sensitivity analysis for the top colocalization, assessing the posterior probability of H0-H4 for different values of prior p_{12}.

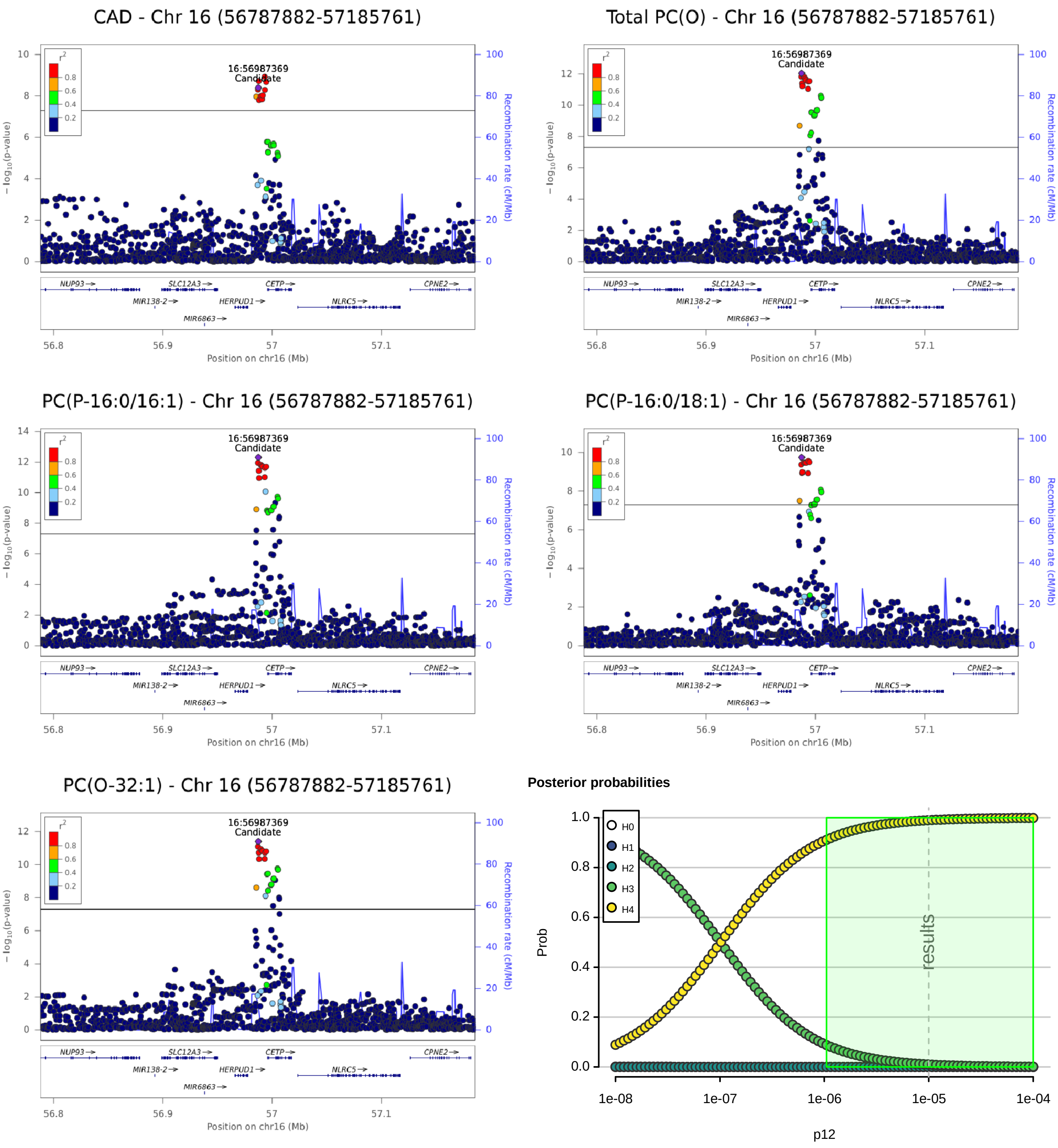

Supplementary Figure 35: Regional association plot of Chr16:56787882-57185761 and coronary artery disease and the top four lipid species that show evidence of colocalization (H3+H4 > 0.8; H4/H3 > 10), along with colocalization sensitivity analysis for the top colocalization, assessing the posterior probability of H0-H4 for different values of prior p_{12}.

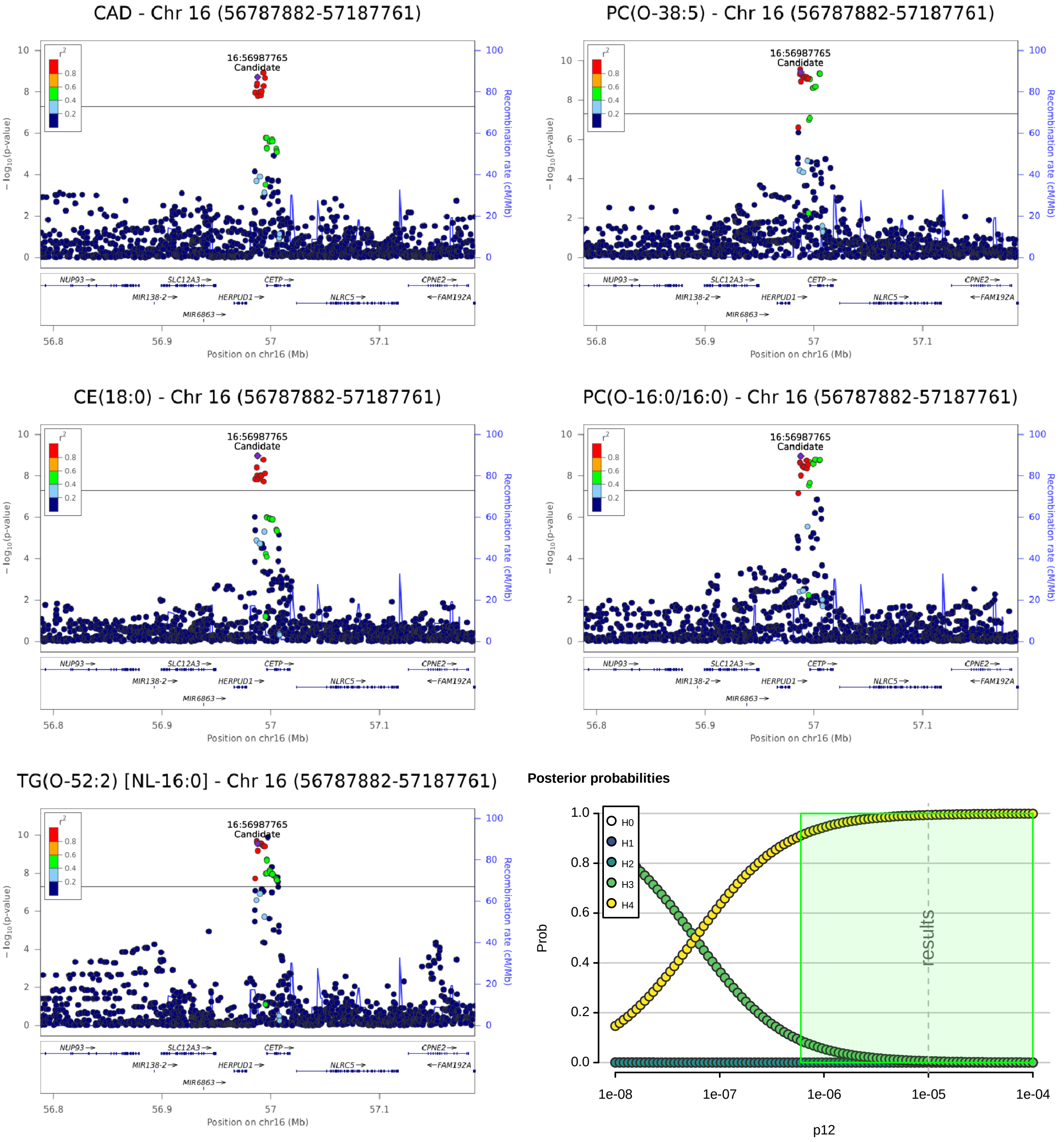

Supplementary Figure 36: Regional association plot of Chr16:56787882-57187761 and coronary artery disease and the top four lipid species that show evidence of colocalization (H3+H4 > 0.8; H4/H3 > 10), along with colocalization sensitivity analysis for the top colocalization, assessing the posterior probability of H0-H4 for different values of prior p_{12}.

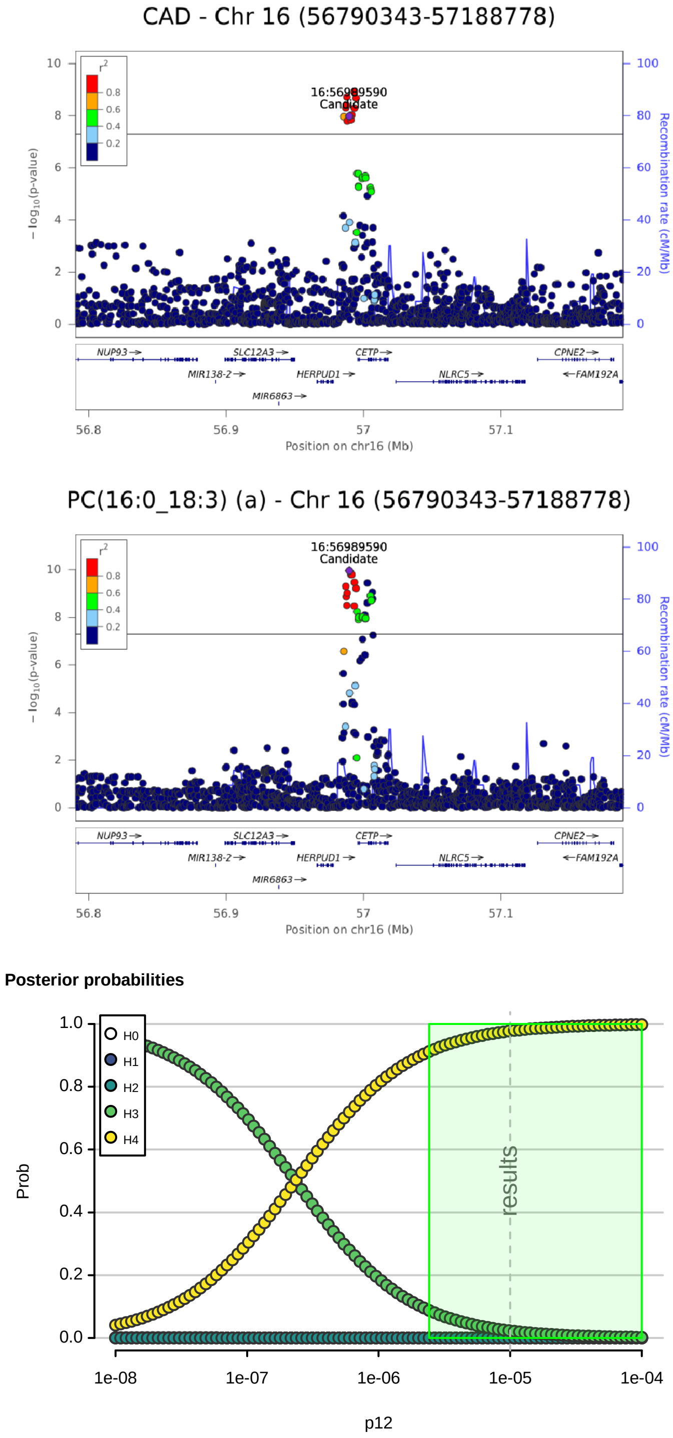

Supplementary Figure 37: Regional association plot of Chr16:56790343-57188778 and coronary artery disease and the top lipid species that shows evidence of colocalization (H3+H4 > 0.8; H4/H3 > 10), along with colocalization sensitivity analysis for the top colocalization, assessing the posterior probability of H0-H4 for different values of prior p_{12}.

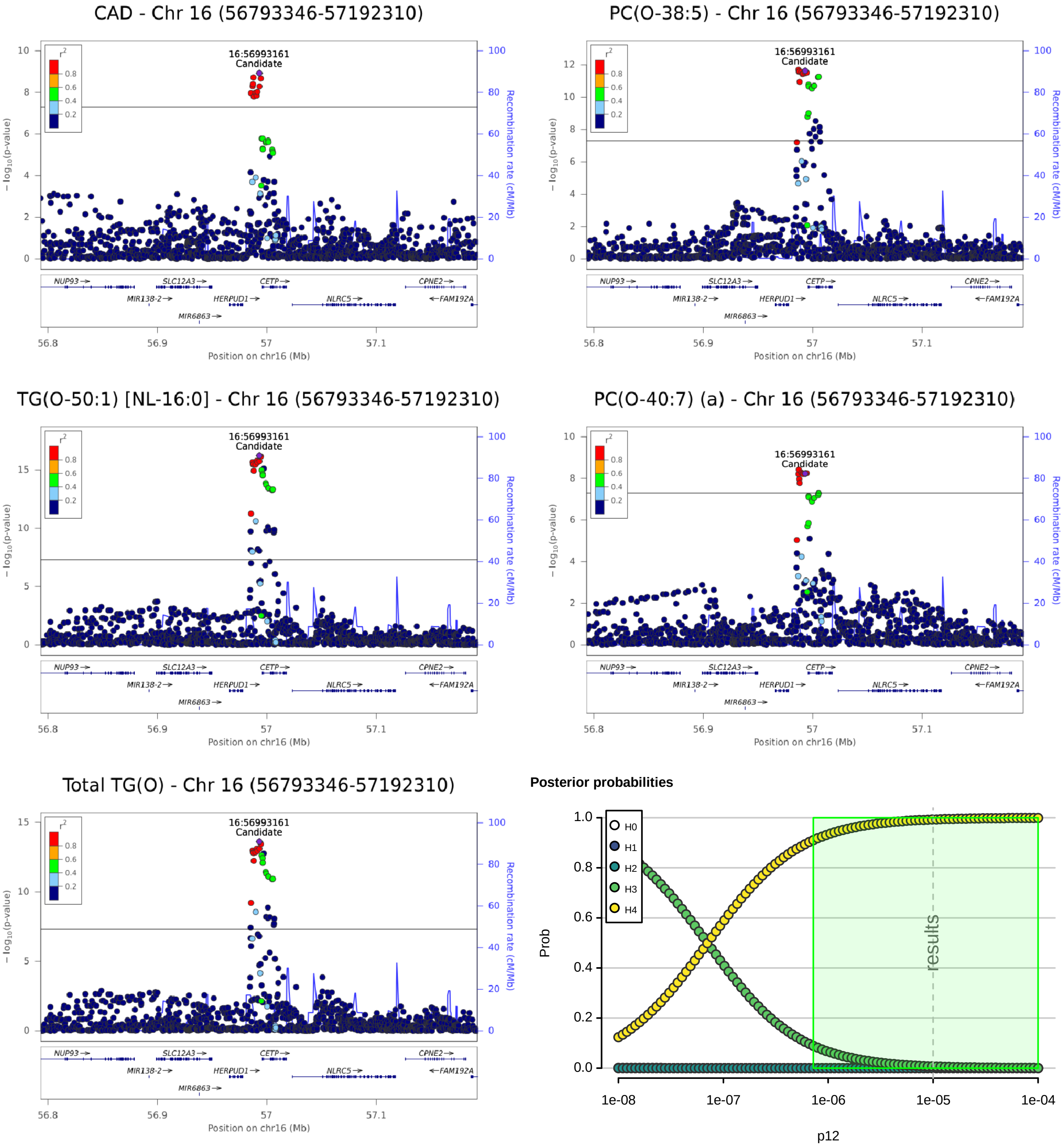

Supplementary Figure 38: Regional association plot of Chr16:56793346-57192310 and coronary artery disease and the top four lipid species that show evidence of colocalization (H3+H4 > 0.8; H4/H3 > 10), along with colocalization sensitivity analysis for the top colocalization, assessing the posterior probability of H0-H4 for different values of prior p_{12}.

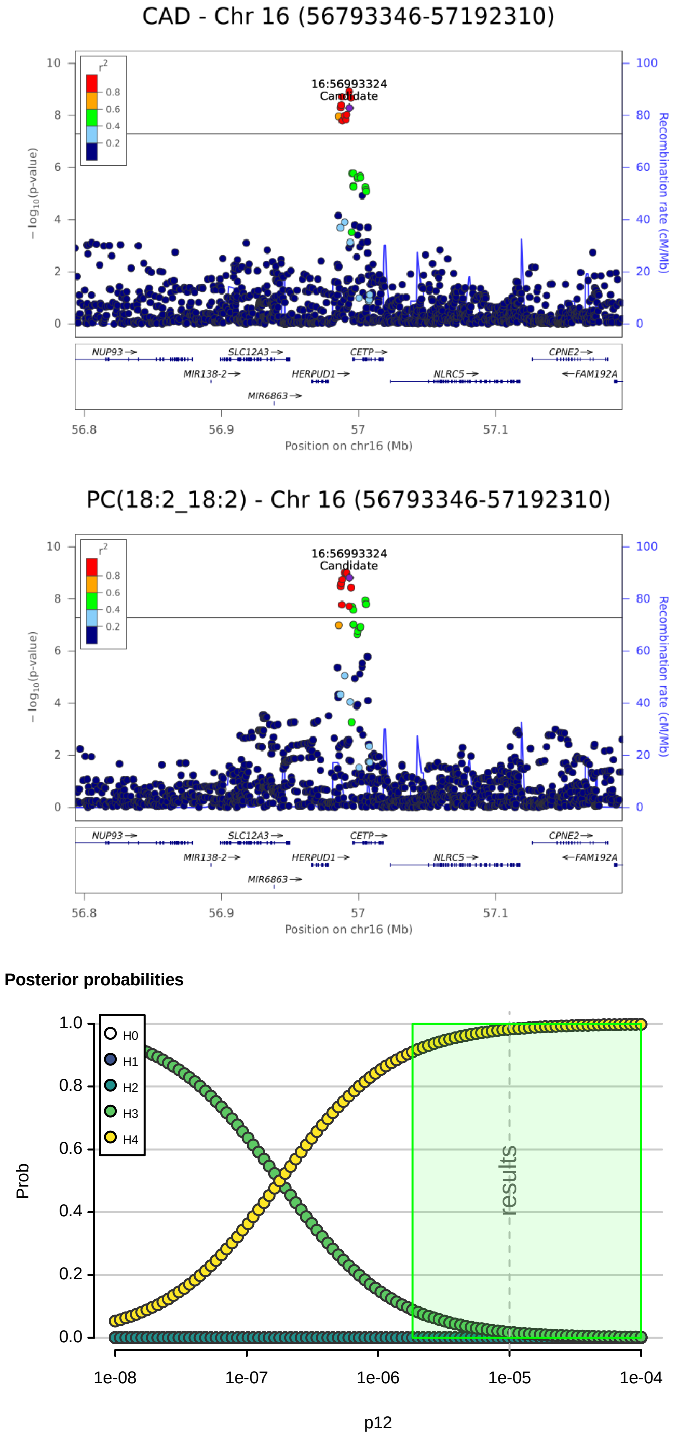

Supplementary Figure 39: Regional association plot of Chr16:56793346-57192310 and coronary artery disease and the top lipid species that shows evidence of colocalization (H3+H4 > 0.8; H4/H3 > 10), along with colocalization sensitivity analysis for the top colocalization, assessing the posterior probability of H0-H4 for different values of prior p_{12}.

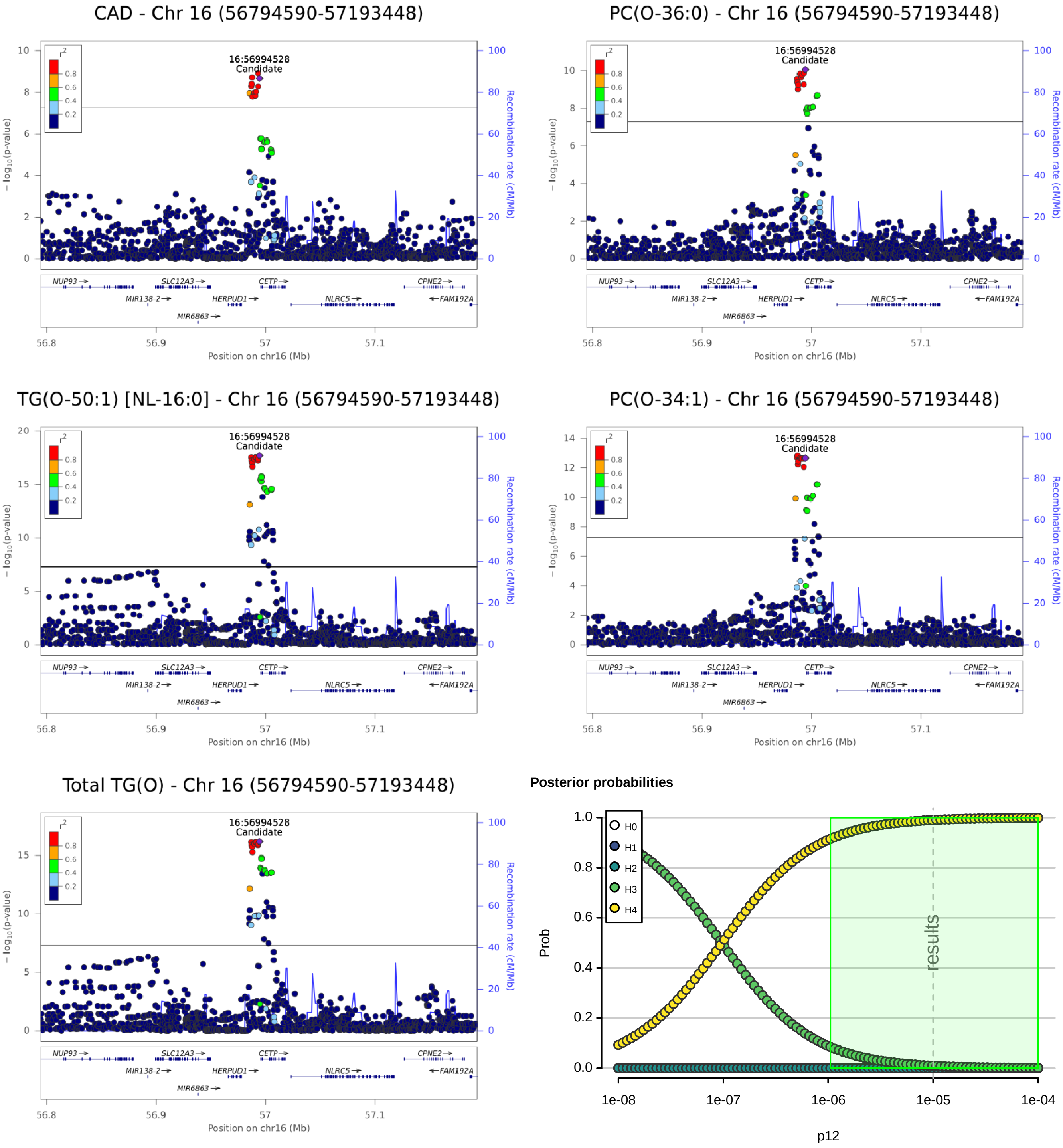

Supplementary Figure 40: Regional association plot of Chr16:56794590-57193448 and coronary artery disease and the top four lipid species that show evidence of colocalization (H3+H4 > 0.8; H4/H3 > 10), along with colocalization sensitivity analysis for the top colocalization, assessing the posterior probability of H0-H4 for different values of prior p_{12}.

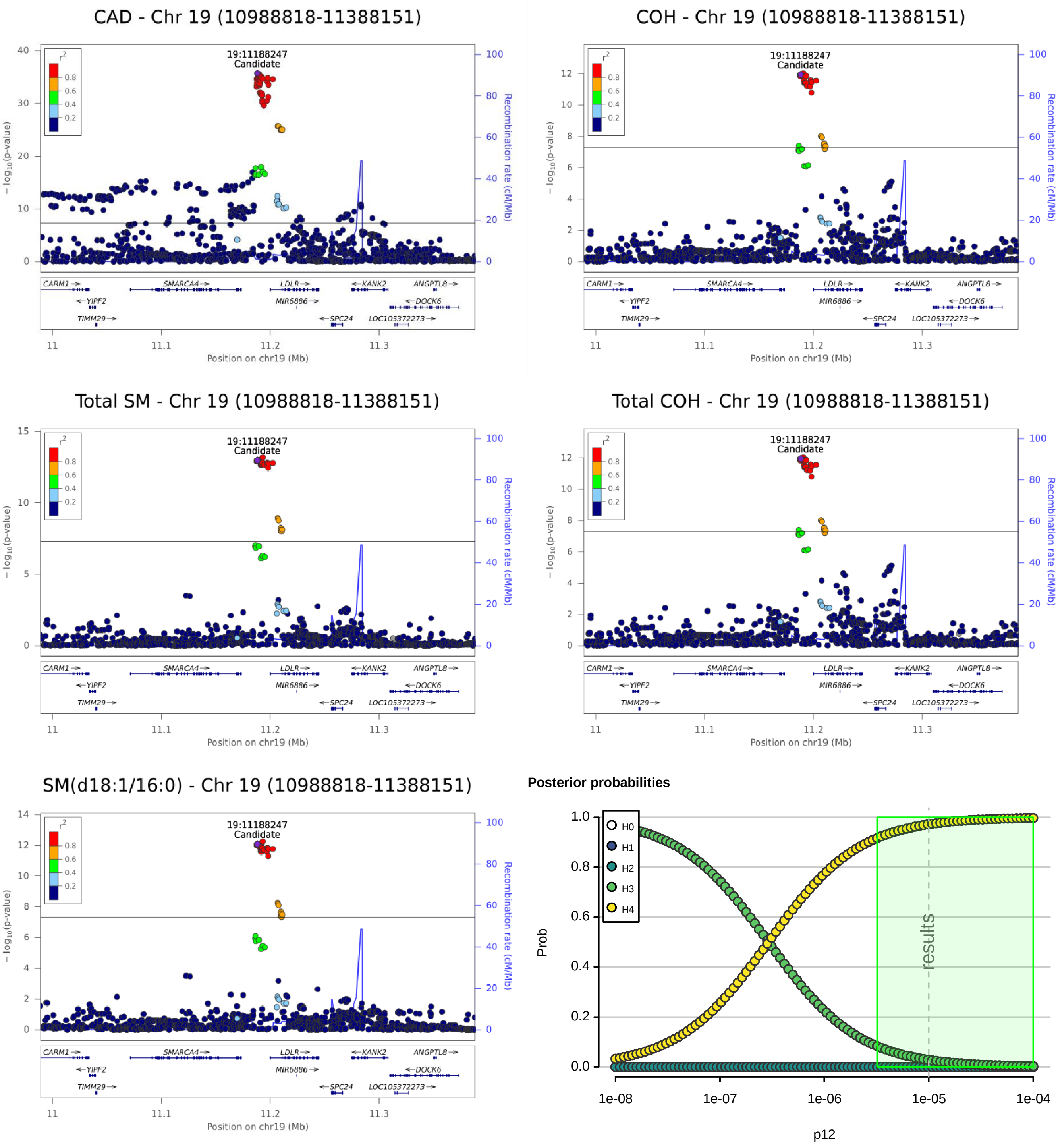

Supplementary Figure 41: Regional association plot of Chr19:10988818-11388151 and coronary artery disease and the top four lipid species that show evidence of colocalization (H3+H4 > 0.8; H4/H3 > 10), along with colocalization sensitivity analysis for the top colocalization, assessing the posterior probability of H0-H4 for different values of prior p_{12}.

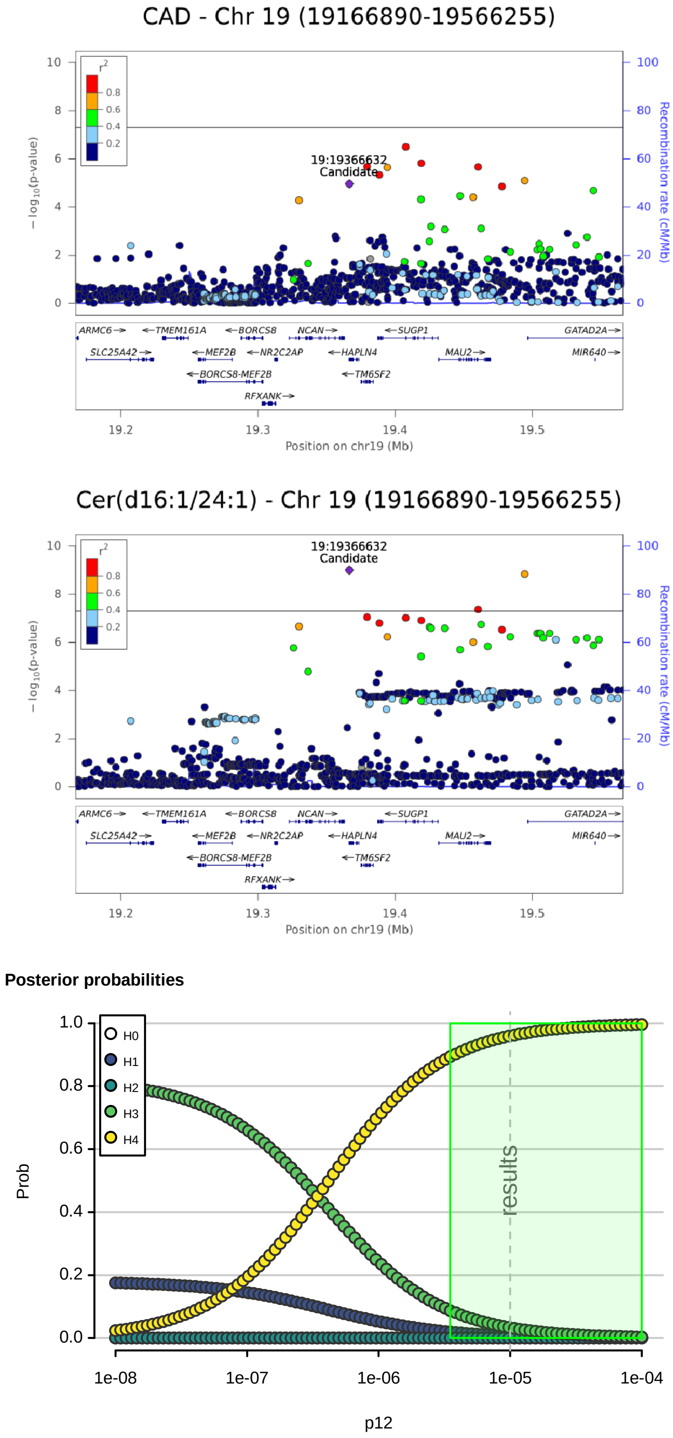

Supplementary Figure 42: Regional association plot of Chr19:19166890-19566255 and coronary artery disease and the top lipid species that shows evidence of colocalization (H3+H4 > 0.8; H4/H3 > 10), along with colocalization sensitivity analysis for the top colocalization, assessing the posterior probability of H0-H4 for different values of prior p_{12}.

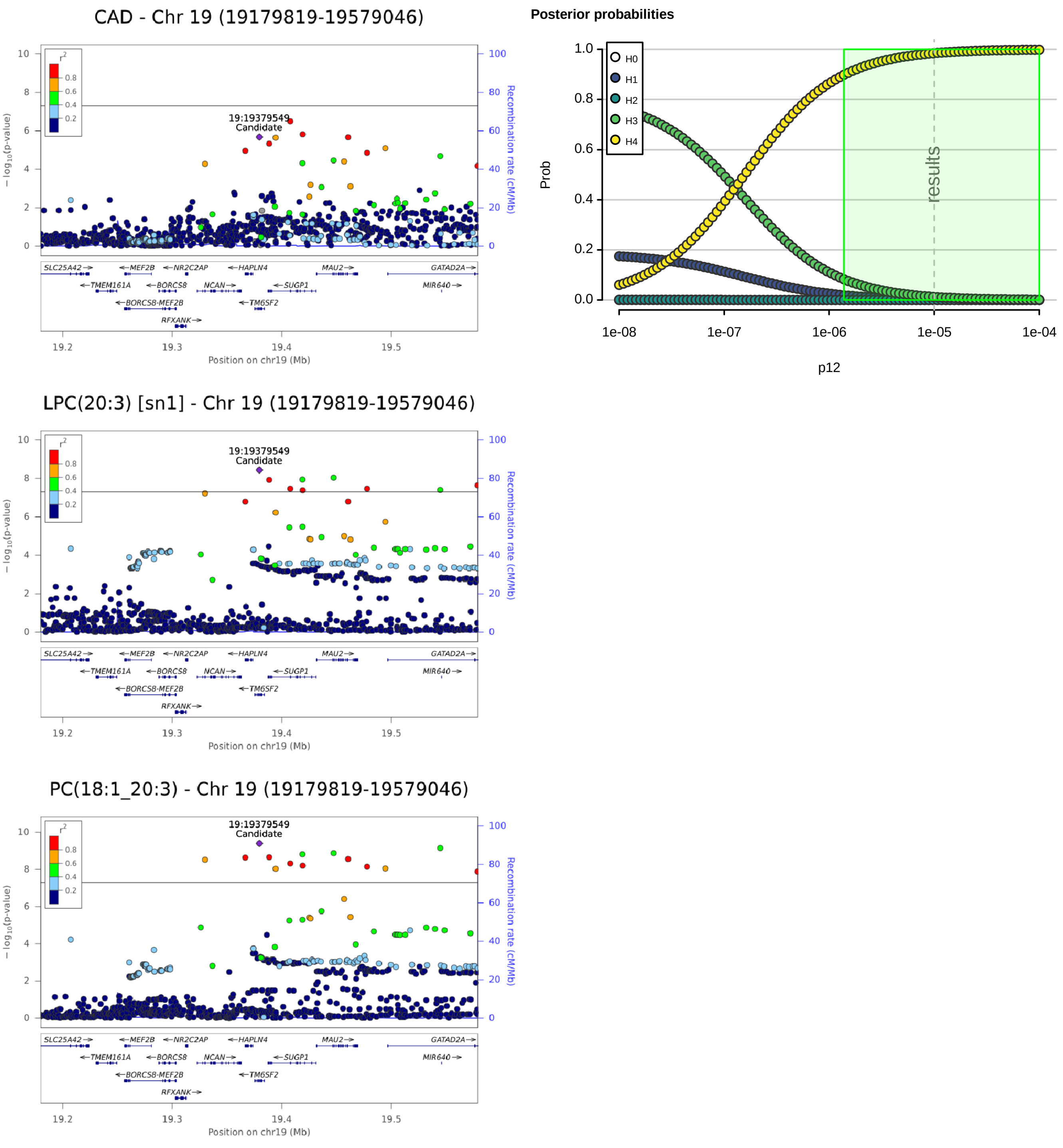

Supplementary Figure 43: Regional association plot of Chr19:19179819-19579046 and coronary artery disease and the top two lipid species that show evidence of colocalization (H3+H4 > 0.8; H4/H3 > 10), along with colocalization sensitivity analysis for the top colocalization, assessing the posterior probability of H0-H4 for different values of prior p_{12}.

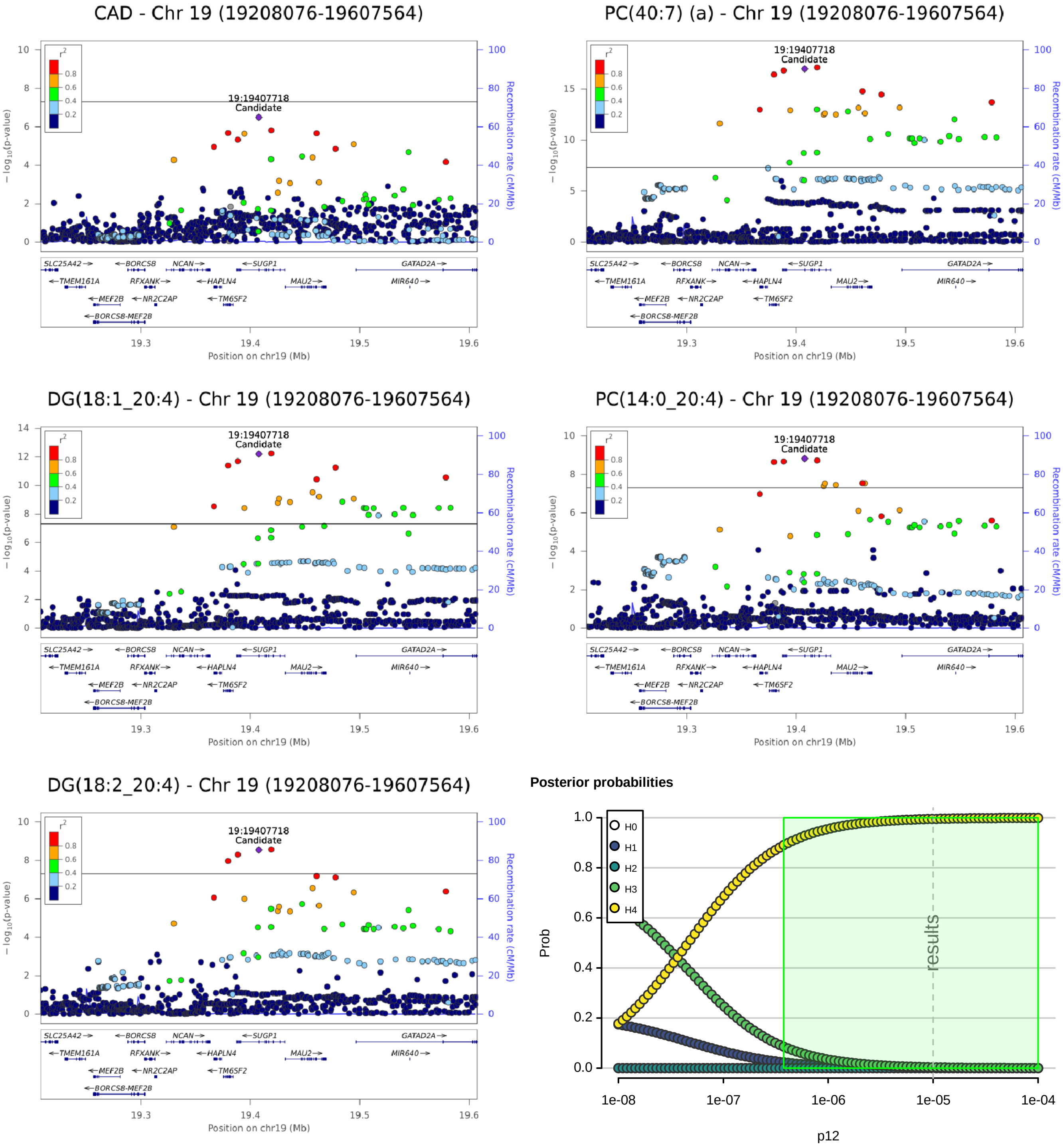

Supplementary Figure 44: Regional association plot of Chr19:19208076-19607564 and coronary artery disease and the top four lipid species that show evidence of colocalization (H3+H4 > 0.8; H4/H3 > 10), along with colocalization sensitivity analysis for the top colocalization, assessing the posterior probability of H0-H4 for different values of prior p_{12}.

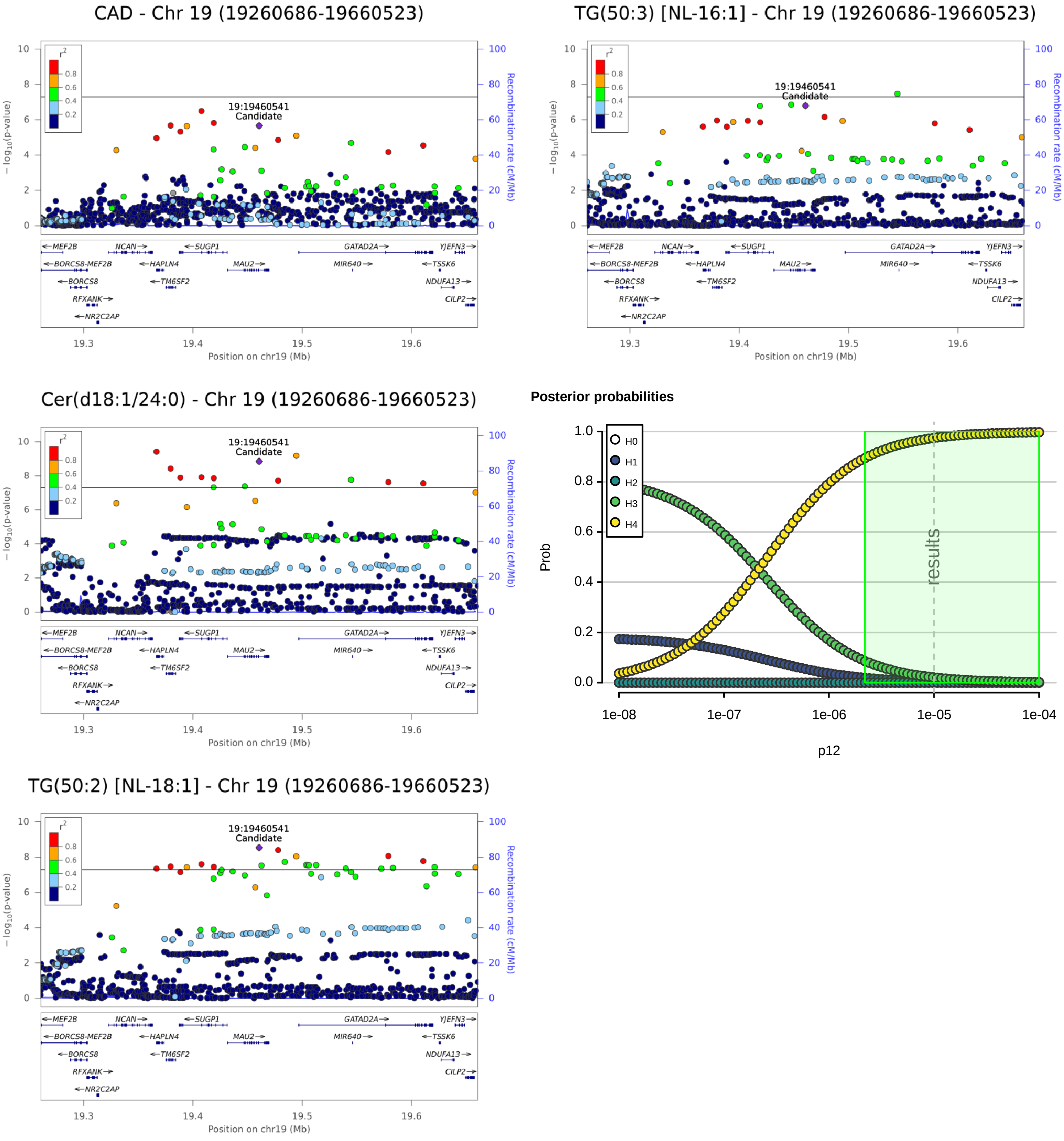

Supplementary Figure 45: Regional association plot of Chr19:19260686-19660523 and coronary artery disease and the top three lipid species that show evidence of colocalization (H3+H4 > 0.8; H4/H3 > 10), along with colocalization sensitivity analysis for the top colocalization, assessing the posterior probability of H0-H4 for different values of prior p_{12}.

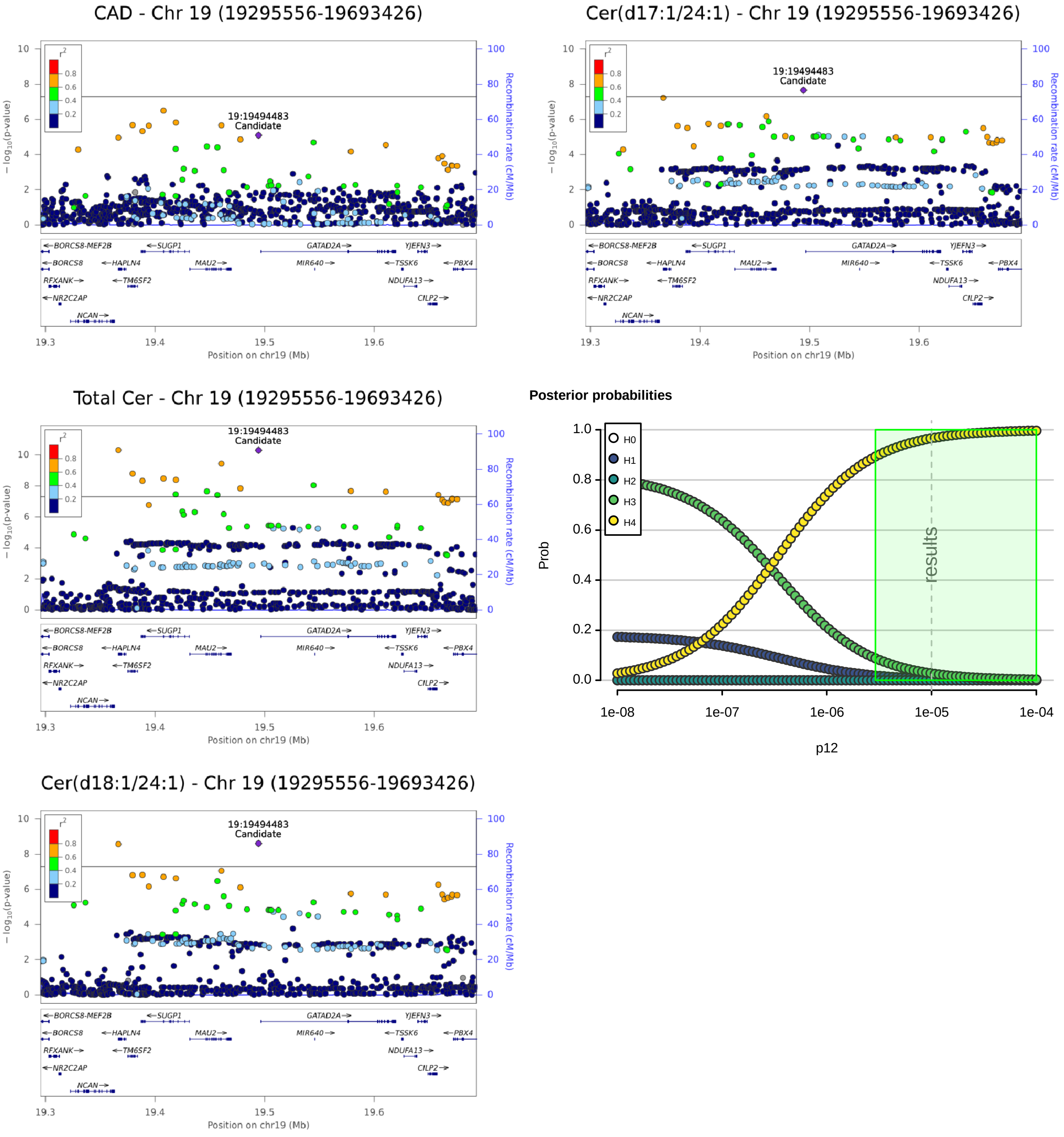

Supplementary Figure 46: Regional association plot of Chr19:19295556-19693426 and coronary artery disease and the top three lipid species that show evidence of colocalization (H3+H4 > 0.8; H4/H3 > 10), along with colocalization sensitivity analysis for the top colocalization, assessing the posterior probability of H0-H4 for different values of prior p_{12}.

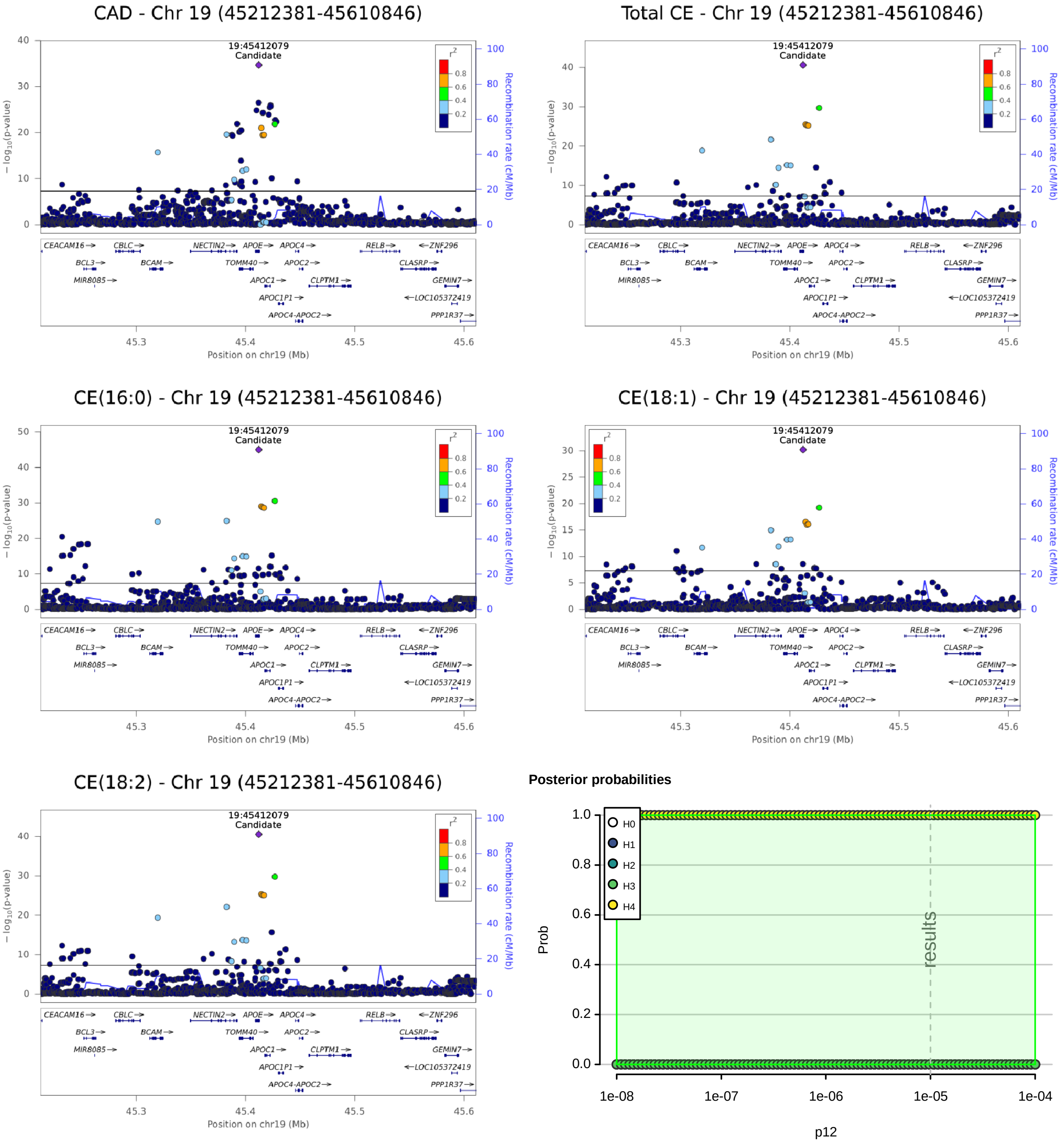

Supplementary Figure 47: Regional association plot of Chr19:45212381-45610846 and coronary artery disease and the top four lipid species that show evidence of colocalization (H3+H4 > 0.8; H4/H3 > 10), along with colocalization sensitivity analysis for the top colocalization, assessing the posterior probability of H0-H4 for different values of prior p_{12}.

Supplementary Note 2: Comparison of adjustment for lipid-lowering medication methods. Inclusion of medication usage as a binary covariate in the analysis of a quantitative trait (which is the target of said medication) can result in loss of power and bias[37]. The addition/multiplication of observed measures with a constant value has been used with success in genome-wide association studies of blood-based biomarkers in subjects taking lipid-lowering medication[38]. Due to the large number of ADNI participants taking lipid-lowering medication (49 %), we compare association statistics of genome-wide significant SNPs with lipid species corrected using two methods: a) the addition of a correction factor derived from the change between pre- and on-statin measurements in LIPID study participants[39], b) the inclusion of lipid-lowering medication as a covariate during adjustment and calculation of residuals.

As shown in Supplementary Figure 48, there was a very high correlation between beta coefficients estimated using both correction procedures. Despite the high usage of lipid-lowering medication in the ADNI cohort, the inclusion of lipid-lowering medication as a covariate did not result in loss of power, nor biased associations.

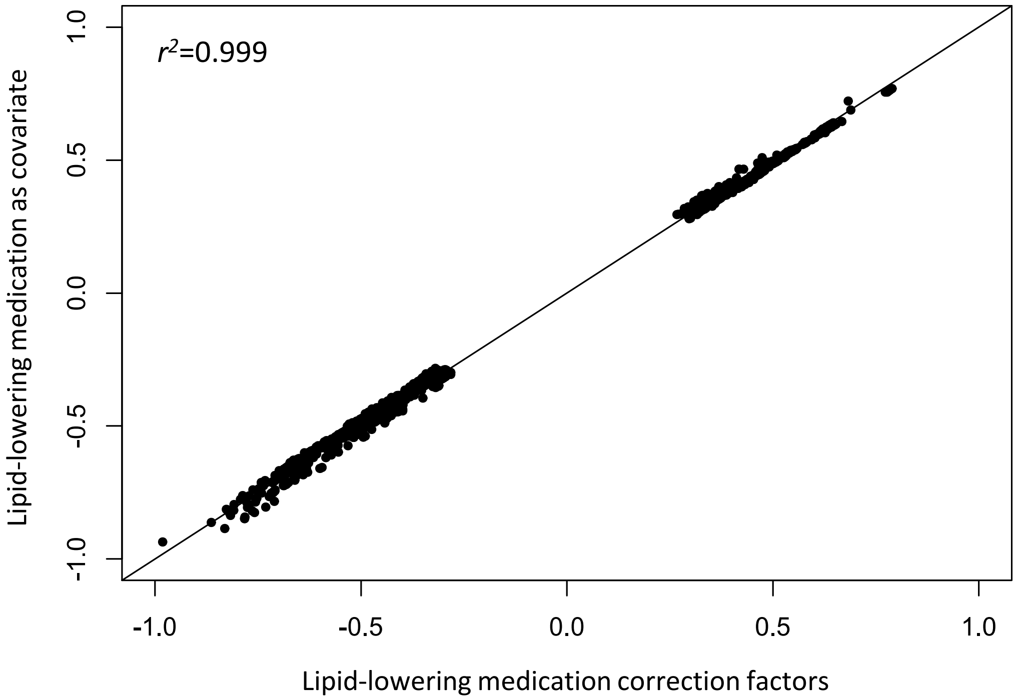

Supplementary Figure 48. Correlation between genome-wide significant beta coefficients following adjustment for lipid-lowering medication using two methods. Lipidomic measurements were adjusted for lipid-lowering medication through calculation of correction factors (x-axis), or by inclusion of a binary variable indicating lipid-lowering medication usage (y-axis).

Supplementary Note 3: Comparison of estimated lipidomic effect sizes. To assess the impact of adjusted for clinical lipid traits or to assess the difference between adjusted and mtCOJO GWAS results, we calculated *t*-statistics for the difference in beta-coefficients.

$$t = \frac{\beta_{STD} - \beta_{ADJ}}{\sqrt{SE(\beta_{STD})^2 + SE(\beta_{ADJ})^2}}$$

Where β_{ADJ} and β_{STD} are the estimated genetic effects from models with and without adjustment for clinical lipid traits, respectively. SE(β) is the estimated standard error of the estimates.

Supplementary References

1. Burkhardt, R. *et al.* Integration of Genome-Wide SNP Data and Gene-Expression Profiles Reveals Six Novel Loci and Regulatory Mechanisms for Amino Acids and Acylcarnitines in Whole Blood. *PLoS Genet* **11**, e1005510 (2015).
2. Chai, J.F. *et al.* Associations with metabolites in Chinese suggest new metabolic roles in Alzheimer's and Parkinson's diseases. *Hum Mol Genet* **29**, 189-201 (2020).
3. Chasman, D.I. *et al.* Forty-three loci associated with plasma lipoprotein size, concentration, and cholesterol content in genome-wide analysis. *PLoS Genet* **5**, e1000730 (2009).
4. Davis, J.P. *et al.* Common, low-frequency, and rare genetic variants associated with lipoprotein subclasses and triglyceride measures in Finnish men from the METSIM study. *PLoS Genet* **13**, e1007079 (2017).
5. Demirkan, A. *et al.* Genome-wide association study identifies novel loci associated with circulating phospho- and sphingolipid concentrations. *PLoS Genet* **8**, e1002490 (2012).
6. Draisma, H.H.M. *et al.* Genome-wide association study identifies novel genetic variants contributing to variation in blood metabolite levels. *Nat Commun* **6**, 7208 (2015).
7. Feofanova, E.V. *et al.* Sequence-Based Analysis of Lipid-Related Metabolites in a Multiethnic Study. *Genetics* **209**, 607-616 (2018).
8. Gieger, C. *et al.* Genetics meets metabolomics: a genome-wide association study of metabolite profiles in human serum. *PLoS Genet* **4**, e1000282 (2008).
9. Harshfield, E.L. *et al.* Genome-wide analysis of blood lipid metabolites in over 5,000 South Asians reveals biological insights at cardiometabolic disease loci. *medRxiv*, 2020.10.16.20213520 (2020).
10. Hartiala, J.A. *et al.* Genome-wide association study and targeted metabolomics identifies sex-specific association of CPS1 with coronary artery disease. *Nat Commun* **7**, 10558 (2016).
11. Hicks, A.A. *et al.* Genetic determinants of circulating sphingolipid concentrations in European populations. *PLoS Genet* **5**, e1000672 (2009).
12. Hong, M.G. *et al.* A genome-wide assessment of variability in human serum metabolism. *Hum Mutat* **34**, 515-24 (2013).
13. Hu, Y. *et al.* Discovery and fine-mapping of loci associated with MUFAs through trans-ethnic meta-analysis in Chinese and European populations. *J Lipid Res* **58**, 974-981 (2017).
14. Illig, T. *et al.* A genome-wide perspective of genetic variation in human metabolism. *Nat Genet* **42**, 137-41 (2010).
15. Inouye, M. *et al.* Novel Loci for metabolic networks and multi-tissue expression studies reveal genes for atherosclerosis. *PLoS Genet* **8**, e1002907 (2012).
16. Kalsbeek, A. *et al.* A genome-wide association study of red-blood cell fatty acids and ratios incorporating dietary covariates: Framingham Heart Study Offspring Cohort. *PLoS One* **13**, e0194882 (2018).
17. Kettunen, J. *et al.* Genome-wide association study identifies multiple loci influencing human serum metabolite levels. *Nat Genet* **44**, 269-76 (2012).
18. Kettunen, J. *et al.* Genome-wide study for circulating metabolites identifies 62 loci and reveals novel systemic effects of LPA. *Nat Commun* **7**, 11122 (2016).
19. Krumsiek, J. *et al.* Mining the unknown: a systems approach to metabolite identification combining genetic and metabolic information. *PLoS Genet* **8**, e1003005 (2012).
20. Long, T. *et al.* Whole-genome sequencing identifies common-to-rare variants associated with human blood metabolites. *Nat Genet* **49**, 568-578 (2017).
21. Lotta, L.A. *et al.* Genetic Predisposition to an Impaired Metabolism of the Branched-Chain Amino Acids and Risk of Type 2 Diabetes: A Mendelian Randomisation Analysis. *PLoS Med* **13**, e1002179 (2016).
22. Lotta, L.A. *et al.* A cross-platform approach identifies genetic regulators of human metabolism and health. *Nat Genet* **53**, 54-64 (2021).

23. Mittelstrass, K. *et al.* Discovery of sexual dimorphisms in metabolic and genetic biomarkers. *PLoS Genet* **7**, e1002215 (2011).
24. Nicholson, G. *et al.* A genome-wide metabolic QTL analysis in Europeans implicates two loci shaped by recent positive selection. *PLoS Genet* **7**, e1002270 (2011).
25. Raffler, J. *et al.* Genome-Wide Association Study with Targeted and Non-targeted NMR Metabolomics Identifies 15 Novel Loci of Urinary Human Metabolic Individuality. *PLoS Genet* **11**, e1005487 (2015).
26. Rhee, E.P. *et al.* A genome-wide association study of the human metabolome in a community-based cohort. *Cell Metab* **18**, 130-43 (2013).
27. Shin, S.Y. *et al.* An atlas of genetic influences on human blood metabolites. *Nat Genet* **46**, 543-550 (2014).
28. Arnold, M., Raffler, J., Pfeufer, A., Suhre, K. & Kastenmüller, G. SNiPA: an interactive, genetic variant-centered annotation browser. *Bioinformatics* **31**, 1334-6 (2015).
29. Suhre, K. *et al.* Human metabolic individuality in biomedical and pharmaceutical research. *Nature* **477**, 54-60 (2011).
30. Tabassum, R. *et al.* Genetic architecture of human plasma lipidome and its link to cardiovascular disease. *Nat Commun* **10**, 4329 (2019).
31. Teslovich, T.M. *et al.* Identification of seven novel loci associated with amino acid levels using single-variant and gene-based tests in 8545 Finnish men from the METSIM study. *Hum Mol Genet* **27**, 1664-1674 (2018).
32. Tukiainen, T. *et al.* Detailed metabolic and genetic characterization reveals new associations for 30 known lipid loci. *Hum Mol Genet* **21**, 1444-55 (2012).
33. Xie, W. *et al.* Genetic variants associated with glycine metabolism and their role in insulin sensitivity and type 2 diabetes. *Diabetes* **62**, 2141-50 (2013).
34. Yet, I. *et al.* Genetic Influences on Metabolite Levels: A Comparison across Metabolomic Platforms. *PLoS One* **11**, e0153672 (2016).
35. Yu, B. *et al.* Whole genome sequence analysis of serum amino acid levels. *Genome Biol* **17**, 237 (2016).
36. van der Harst, P. & Verweij, N. Identification of 64 Novel Genetic Loci Provides an Expanded View on the Genetic Architecture of Coronary Artery Disease. *Circ Res* **122**, 433-443 (2018).
37. Tobin, M.D., Sheehan, N.A., Scurrah, K.J. & Burton, P.R. Adjusting for treatment effects in studies of quantitative traits: antihypertensive therapy and systolic blood pressure. *Stat Med* **24**, 2911-35 (2005).
38. Sinnott-Armstrong, N. *et al.* Genetics of 35 blood and urine biomarkers in the UK Biobank. *Nature Genetics* **53**, 185-194 (2021).
39. Jayawardana, K.S. *et al.* Changes in plasma lipids predict pravastatin efficacy in secondary prevention. *JCI Insight* **4** (2019).
