# Supplementary material for: Comprehensive genetic analysis of the human lipidome identifies loci associated with lipid homeostasis with links to coronary artery disease
Source: Nat Commun. 2022 Jun 6;13:3124. doi: 10.1038/s41467-022-30875-7 (PMC9170690; doi:10.1038/s41467-022-30875-7)
Supplement: Supplementary file 2 — Description of Additional Supplementary Files [file 41467_2022_30875_MOESM2_ESM.pdf]

Description of Additional Supplementary Files

File Name: Supplementary Data 1
Description: Details of lipidomic analyses in the Busselton Health Study

File Name: Supplementary Data 2
Description: Details of lipid species and measurements in the Busselton Health Study

File Name: Supplementary Data 3
Description: Association of genetic variants against serum lipid classes in the Busselton Health Study

File Name: Supplementary Data 4
Description: Association of genetic variants against serum lipid species in the Busselton Health Study

File Name: Supplementary Data 5
Description: Annotation of genome-wide significant SNPs

File Name: Supplementary Data 6
Description: Identification of independent lipid loci

File Name: Supplementary Data 7
Description: Results of conditional analysis and secondary signals in GWAS

File Name: Supplementary Data 8
Description: Validation of independent genome-wide significant associations in replication cohorts

File Name: Supplementary Data 9
Description: Discovery meta-analysis in ADNI/AIBL/BHS

File Name: Supplementary Data 10
Description: Identification of candidate causal genes using genetic prioritization and knowledge-based approaches

File Name: Supplementary Data 11
Description: Genetic-based prioritization of candidate genes

File Name: Supplementary Data 12
Description: Biological knowledge-based prioritization of candidate genes

File Name: Supplementary Data 13
Description: Overlap of lead SNPs with previous lipid/metabolite associations

File Name: Supplementary Data 14
Description: Overlap of associations in the GWAS catalog and CardiogramplusC4D

File Name: Supplementary Data 15
Description: Relationship of serum lipid species with CVD, including phenotypic associations, genetic correlations and association with PRS

File Name: Supplementary Data 16
Description: Colocalisation analysis and shared causal variants for coronary artery disease

File Name: Supplementary Data 17
Description: Association of lipid loci with coronary atherosclerosis in the UK Biobank
