# Supplementary material for: Comprehensive genetic analysis of the human lipidome identifies loci associated with lipid homeostasis with links to coronary artery disease
Source: Nat Commun. 2022 Jun 6;13:3124. doi: 10.1038/s41467-022-30875-7 (PMC9170690; doi:10.1038/s41467-022-30875-7)
Supplement: Supplementary file 20 — Reporting Summary [file 41467_2022_30875_MOESM20_ESM.pdf]

nature portfolio

Corresponding author(s): Professors Peter Meikle and Eric Moses

Last updated by author(s): Aug 9, 2021

Reporting Summary

Nature Portfolio wishes to improve the reproducibility of the work that we publish. This form provides structure for consistency and transparency in reporting. For further information on Nature Portfolio policies, see our Editorial Policies and the Editorial Policy Checklist.

Statistics

For all statistical analyses, confirm that the following items are present in the figure legend, table legend, main text, or Methods section.

n/a	Confirmed	
☐	☒	The exact sample size (*n*) for each experimental group/condition, given as a discrete number and unit of measurement
☒	☐	A statement on whether measurements were taken from distinct samples or whether the same sample was measured repeatedly
☐	☒	The statistical test(s) used AND whether they are one- or two-sided<br>*Only common tests should be described solely by name; describe more complex techniques in the Methods section.*
☐	☒	A description of all covariates tested
☐	☒	A description of any assumptions or corrections, such as tests of normality and adjustment for multiple comparisons
☐	☒	A full description of the statistical parameters including central tendency (e.g. means) or other basic estimates (e.g. regression coefficient) AND variation (e.g. standard deviation) or associated estimates of uncertainty (e.g. confidence intervals)
☐	☒	For null hypothesis testing, the test statistic (e.g. *F*, *t*, *r*) with confidence intervals, effect sizes, degrees of freedom and *P* value noted<br>*Give P values as exact values whenever suitable.*
☒	☐	For Bayesian analysis, information on the choice of priors and Markov chain Monte Carlo settings
☒	☐	For hierarchical and complex designs, identification of the appropriate level for tests and full reporting of outcomes
☐	☒	Estimates of effect sizes (e.g. Cohen's *d*, Pearson's *r*), indicating how they were calculated

Our web collection on statistics for biologists contains articles on many of the points above.

Software and code

Policy information about availability of computer code

Data collection	Data consisted of genetic, lipidomic and epidemiological data. Genetic data was in the form of imputed genotypes (imputed to the Haplotype Reference Consortium using the Michigan Imputation Server). Lipidomic profiling was performed using liquid chromatography coupled electrospray ionization-tandem mass spectrometry and analysed using MassHunter Quant B08 (Agilent Technologies). Details of all data collection are described within the Methods section of the manuscript.
Data analysis	Data analysis was performed as described in the Methods section of the manuscript. Quality control of the genetic data was performed using Plink (version 1.9 and version 2), and imputation was performed using the Michigan Imputation Server (version 1.2.4). Genome-wide association analyses were performed using GEMMA (version 0.98) in the discovery cohort, Plink for the two validation cohorts, and adjustment for clinical lipids was performed using GCTA (version 1.93.2). Meta-analyses were performed using METAL. Genetic correlations were calculated using Linkage Disequilibrium Score Regression, and co-localization analyses were performed in R using the COLOC package. The R package PheWAS was used to convert electronic health records into PheCodes.

For manuscripts utilizing custom algorithms or software that are central to the research but not yet described in published literature, software must be made available to editors and reviewers. We strongly encourage code deposition in a community repository (e.g. GitHub). See the Nature Portfolio guidelines for submitting code & software for further information.

Data

Policy information about availability of data

All manuscripts must include a data availability statement. This statement should provide the following information, where applicable:

- Accession codes, unique identifiers, or web links for publicly available datasets
- A description of any restrictions on data availability
- For clinical datasets or third party data, please ensure that the statement adheres to our policy

Complete summary statistics of all lipid species and classes are available via the NHGRI-EBI GWAS catalog (https://www.ebi.ac.uk/gwas), GCP ID: GCP000197; study accession nos. GCST90023981–GCST90025848. In addition, summary-level statistics are available at our data portal (https://metabolomics.baker.edu.au/).

Individual-level data for the BHS are accessible through applications to the Busselton Population Medical Research Institute (http://bpmri.org.au/research/database-access.html). Individual-level data for the ADNI and AIBL studies are available through applications to the LONI Image and Data Archive (http://adni.loni.usc.edu/data-samples/access-data/). Individual-level data for AIBL are also available through applications to the AIBL management committee (https://aibl.csiro.au/research/support/).

Publically available datasets used within the study are available via UK Biobank (http://www.ukbiobank.ac.uk/register-apply/), HRC (http://www.haplotype-reference-consortium.org/home), 1000 Genomes (https://www.internationalgenome.org/), SNiPA (https://snipa.helmholtz-muenchen.de/snipa3/), GTEx (https://gtexportal.org/home/), and eQTLGen (https://www.eqtlgen.org/).

Field-specific reporting

Please select the one below that is the best fit for your research. If you are not sure, read the appropriate sections before making your selection.

☒ Life sciences ☐ Behavioural & social sciences ☐ Ecological, evolutionary & environmental sciences

For a reference copy of the document with all sections, see nature.com/documents/nr-reporting-summary-flat.pdf

Life sciences study design

All studies must disclose on these points even when the disclosure is negative.

Sample size	We have performed one of the largest lipidomic GWAS of 596 lipid species in 4,492 people, and performed validation in 1,565 people. We utilised all people within the discovery cohort for whom genome-wide SNP data, extensive longitudinal phenotype data, and blood serum were available. Sample size for the validation cohorts (ADNI and AIBL) was obtained based on the availability of samples.
Data exclusions	People were excluded if they had >3% of SNP data missing, reported sex did not match genotyped sex, were missing phenotype data, or >5 standard deviations above/below mean heterozygosity. Individuals with non-European ancestry were also excluded.
Replication	We performed a validation study of genetic variants and the lipidome in two independent cohorts of 670 and 895 people. We performed analyses separately in these two cohorts, and meta-analysed the two studies using a fixed-effect meta-analysis. Of the 2,137 significant SNP-lipid associations identified in the discovery cohort, identified 1,474 (69.2%) reached nominal significance (P<0.05), and 644 (30.1%) reached Bonferroni-corrected significance in the validation cohort meta-analysis.
Randomization	All samples were randomised prior to lipidomic analyses. Genotyping in all studies was performed independently of the lipidomic and other outcome measures.
Blinding	All samples were deidentified. Genotyping and lipidomic profiling were performed independently, with no prior knowledge of genotype or lipid species measurements.

Reporting for specific materials, systems and methods

We require information from authors about some types of materials, experimental systems and methods used in many studies. Here, indicate whether each material, system or method listed is relevant to your study. If you are not sure if a list item applies to your research, read the appropriate section before selecting a response.

Materials & experimental systems

n/a	Involved in the study
☒	☐ Antibodies
☒	☐ Eukaryotic cell lines
☒	☐ Palaeontology and archaeology
☒	☐ Animals and other organisms
☐	☒ Human research participants
☒	☐ Clinical data
☒	☐ Dual use research of concern

Methods

n/a	Involved in the study
☒	☐ ChIP-seq
☒	☐ Flow cytometry
☒	☐ MRI-based neuroimaging

Human research participants

Policy information about studies involving human research participants

Population characteristics

Population characteristics are described in Supplementary Table 1 of the manuscript. Basic characteristics of the three cohorts with genome-wide imputed data and lipidomic data are below:

1. Busselton Health Study (n=4492), 56% female, mean age of 50.8 (SD:17.4). 2.4% taking lipid-lowering medications. 17.6 with lifetime CAD events, with 12.3% occurring post-collection.
2. Alzheimer's Disease Neuroimaging Initiative (n=670), 60% female, mean age of 75.3 (SD:6.7). 49% taking lipid-lowering medications.
3. Australian Imaging, Biomarker & Lifestyle Study of Ageing (n=895), 43% female, mean age of 74.7 (SD:7.4). 22.1% taking lipid-lowering medications.

Recruitment

Our study did not directly involve the recruitment of participants. Participants in the discovery cohort were all participants of the 1994/95 survey of the long-running epidemiological study, the Busselton Health Study. In 1994/95, all participants in the previous waves of the Busselton Health Study were invited to donate a blood sample and complete assessments. Recruitment for the ADNI and AIBL cohorts has been described extensively in earlier papers (AIBL: Ellis et al. 2009; ADNI: Weiner et al. 2010).

Ethics oversight

Our study did not directly involve the recruitment of participants. Informed consent was obtained from all participants and the 1994/95 Busselton Health Study was approved by the University of Western Australia Human Research Ethics Committee (UWA HREC). The lipidomics and genetic analysis was approved by UWA HREC (RA/4/1/7894) and the Western Australian Department of Health HREC (RGS03656). For the lipidomics analysis, the AIBL study was deemed low risk (The Alfred Ethics Committee; Project 183/19), and the ADNI study was deemed RESEARCH NOT INVOLVING HUMAN SUBJECTS (Duke Institute review board; ID:Pro00053208).

Note that full information on the approval of the study protocol must also be provided in the manuscript.
